# A Material Platform Based on Dissociative CO_2_‑Derived *N,O-*Acetals for Tunable Degradation of 3D Printable Materials

**DOI:** 10.1021/jacs.5c07767

**Published:** 2025-08-07

**Authors:** Marco Caliari, Jacopo Teotonico, Mikel Irigoyen, Anje Mujika, Tommaso Isolabella, Daniele Mantione, Lourdes Irusta, Bruno Grignard, Fernando Vidal, Christophe Detrembleur, Haritz Sardon

**Affiliations:** † POLYMAT and Department of Polymers and Advanced Materials: Physics, Chemistry and Technology, Faculty of Chemistry, 160665 University of the Basque Country UPV/EHU, Paseo Manuel de Lardizábal, 3, 20018 Donostia-San Sebastián, Spain; ‡ Center for Education and Research on Macromolecules (CERM), CESAM Research Unit, 26658 University of Liege, Sart-Tilman B6a, 4000 Liege, Belgium; § Physics Department, University of Genoa & INFN, Division of Genoa, 16146 Genoa, Italy; ∥ Ikerbasque, Basque Foundation for Science, Plaza Euskadi 5, 48009 Bilb ao, Spain; ⊥ FRITCO_2_T Platform, University of Liege, Sart-Tilman B6a, 4000 Liege, Belgium; # WEL Research Institute, Avenue Pasteur 6, 1300 Wavre, Belgium

## Abstract

Modern-day thermosetting polymers should be designed with circular economy principles in mind, considering both their recyclability and end-of-life options. Covalent adaptable networks (CANs) have the potential to address the environmental challenges we face today as, in spite of being thermosets, they can be reprocessed by conventional thermoprocessing methods and are thus recyclable. While in the last years intensive efforts have been devoted to the preparation of CANs using sustainable sources, less attention has been paid to their end-of-life options in case they escape from plastic sorting. Herein, we report the development of a new type of dynamic bond, the *N*,*O*-acetal bond based on the coupling between CO_2_-based oxazolidone moieties and abundant, potentially biobased polyols. Computational and kinetic studies revealed that this bond underwent rapid dissociative exchange and, crucially, was also susceptible to hydrolytic degradation. We then prepared a range of thermoset materials endowed by double end-of-life features, i.e., CAN behavior and hydrolytic degradation. This was achieved by radical thiol–ene photo-cross-linking of a diallyl monomer bearing the *N*,*O*-acetal moiety with another alkene-functionalized monomer that did not bear this dynamic bond. CANs with tunable mechanical properties and hydrolytic degradation features were easily obtained by modulating the monomer compositions. The fast-photocuring of the *N*,*O*-functionalized monomer was then exploited for producing three-dimensional (3D) printed objects, offering the potential for on-demand hydrolytic behavior.

## Graphical abstract

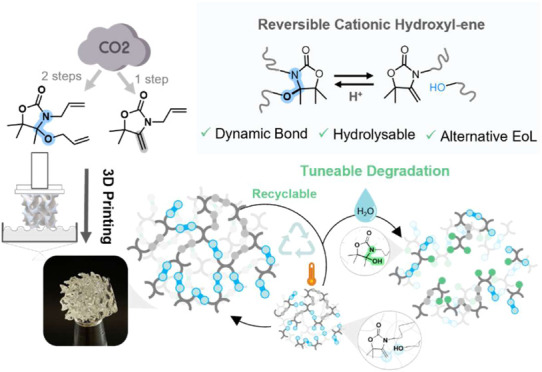

## Introduction

Worldwide plastic production is still heavily linked to the petrochemical industry. While they use only about 6% of the global oil production,^1^ plastics heavily impact the environment and human health by breaking down into microplastics and leaching additives in the environment.^2,3^ Their nonrenewable origin, together with their limited end-of-life options, make plastic sustainability a centerpiece of today’s research in polymeric materials. Mitigation strategies for the nonrenewable origin of plastics include the use of biobased feedstock, recirculated plastic wastes, or captured CO_2_.^4–8^ For instance, biomass-derived alternatives to traditional petrochemical-based plastics have been on the rise.^9^ On the other hand, CO_2_-based strategies^10–12^ have been highlighted as a key step toward a circular plastic economy.^13,14^ Indeed, CO_2_ uptake in plastics has already seen some commercial success for the production of polycarbonate polyols in certain polyurethane applications.^15–18^

Another approach to enhance the sustainability of polymeric materials is the design of circular approaches to facilitate the reutilization and recycling of polymeric materials.^19,20^ Thermosets are in dire need of such revolutionizing technologies as their highly cross-linked structures do not allow their reprocessability via mechanical means restricting their reuse and recyclability, and thus end up being landfilled or incinerated. Alternatively, the introduction of dynamic covalent bonds in polymer networks, also named “covalent adaptable networks” or CANs,^21–23^ has been proposed as one strategy to break this vicious cycle. Specifically, CANs achieve both high mechanical performances and chemical resistance of cross-linked thermosets as well as the ability to be reprocessed by heat-based techniques such as extrusion or injection molding, generally limited to thermoplastics.^24^

Recently, the introduction of captured CO_2_ as a renewable feedstock for the development of CAN thermosets has emerged as an enticing step toward more sustainable plastics.^25,26^ An especially interesting class of CO_2_-derived polymers with the possibility to introduce CANs is nonisocyanate polyurethanes or NIPUs.^10,27–31^ Recently, a new class of NIPUs has been developed by the step-growth polymerization of CO_2_-derived oxazolidones bearing exovinylene groups with thiols. These materials have the added benefit to possess unprecedented dynamic bonds in the form of reversible *N,S-*acetals *via* reaction of the C=C of the oxazolidone moieties with thiols, a feature that was exploited to reprocess thermosets.^25^ Their fast, efficient exchange dynamics enabled unparalleled recyclability and processability by multiple industrially relevant techniques such as extrusion, injection molding, and compression molding. However, the use of sulfur-based reagents as well as the high environmental stability of sulfur-based acetals toward hydrolysis, could result in bioaccumulation of these new plastics in case they escape from plastic sorting, as it is most common in today’s recycling facilities.^32–34^

Hence, the development of degradable scaffolds that ideally turn into harmless chemical fragments is an essential piece for the sustainability of any plastic material.^11,35^ While sulfur-based acetals (*S*,*S*-) are known to display high stability toward hydrolysis,^36–38^ oxygen-based acetals (*O*,*O*-) and nitrogen-based hemiaminals (*N*,*O*-) and aminals (*N*,*N*-) are more prone to this type of chemical degradation into monomer or oligomeric units under acidic conditions.^39,40^ In fact, *O*,*O*-acetals have been previously used to enable the degradation of polyolefins to enhance their sustainability.^41,42^ Furthermore, their degradation was controlled by the addition of a protic species, enabling on-demand degradation. We envision that the preparation of unique *N*,*O*-acetals could enable the formation of dynamic bonds with degradability built into their performance^43–45^

In this work, we propose a new type of dynamic, cleavable bond based on *N,O-*acetals embedded in the polymer repeat-unit heterocycle (Figure 1). First, we demonstrate that this type of moiety is easily installed on a functional oxazolidone precursor at room temperature in minutes under acidic catalysis. Second, mechanistic insights from computational and kinetic studies indicate that this bond is dissociative with quick exchange dynamics. With this knowledge, a diallyl monomer containing a cleavable *N,O-*acetal moiety was prepared and photocured, leading to recyclable CAN materials that can be degraded in water (Figure 1). We controlled the hydrolytic degradation rates by mixing a diene monomer that would add hydrolytically stable thioether moieties in the network (Figure 1a). We exploit the quick curing dynamics to print this material by vat photopolymerization for the production of 3D scaffolds with on-demand hydrolytic degradation (Figure 1c). This study highlights the potential of this new degradable bond to expand the applicability of oxazolidone-based NIPU CANs.

**Figure 1:**
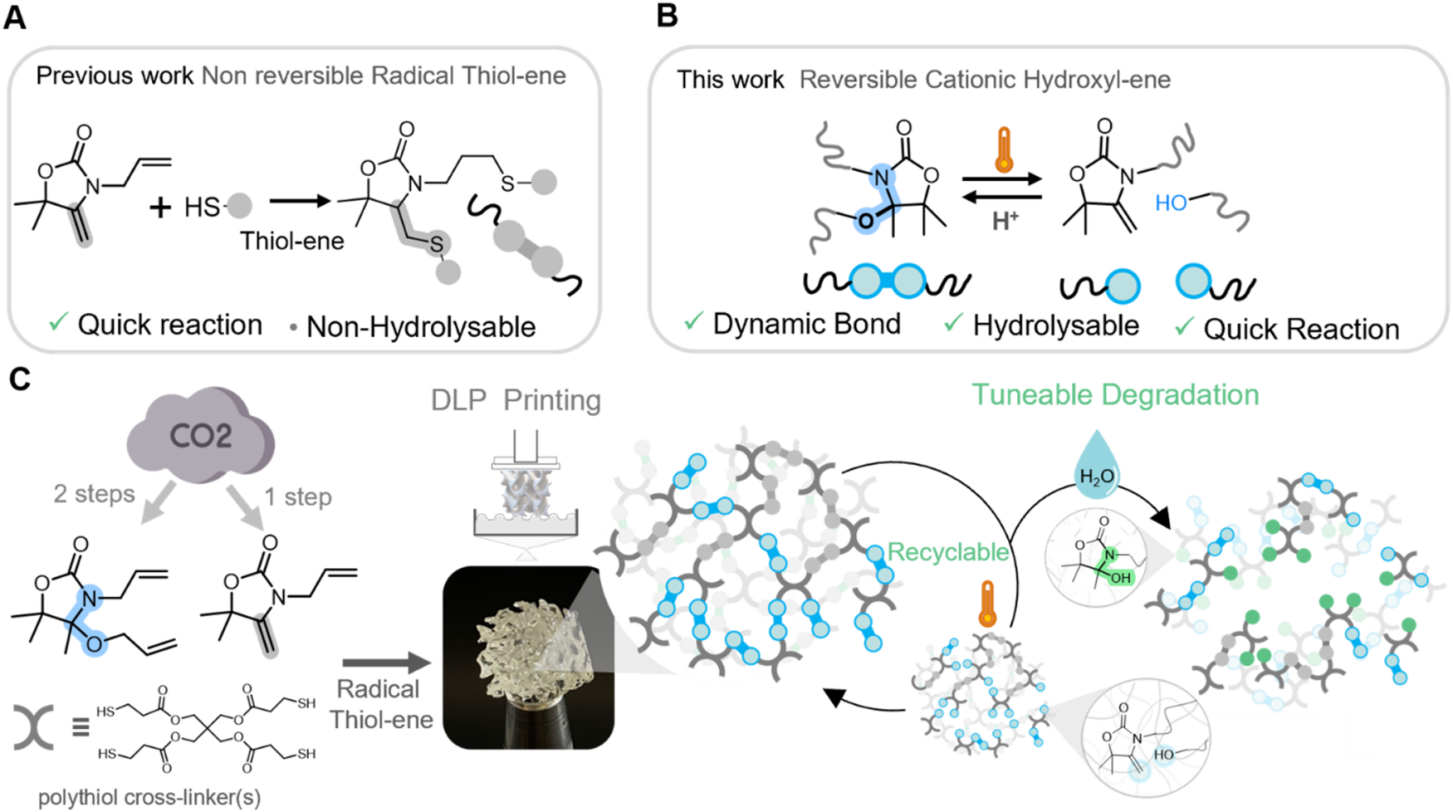
(A) Schematic of the thiol–ene reaction on **AllOx** giving origin to two thioether bonds; (B) schematic of the dynamic *N,O-*acetal chemistry developed in this work; and (C) general scheme of the study in which a diallyl monomer containing *N,O-*acetal together with an allyl-bearing alkylidene oxazolidone monomer could be 3D printed into polyoxazolidone dissociative thermoset bearing varying amounts of hydrolytically degradable bonds. The network could be recycled thanks to the reversible hydroxyl-ene reaction and degraded through hydrolysis, and the rate of hydrolysis could be adjusted by varying the content in the hydrolyzable moiety.

## Results and Discussion

### Small-Molecule Studies

As we have shown previously, alkylidene oxazolidones react efficiently with thiols under acidic catalysis to form *N,S-*acetals by addition of the thiol to the C=C double bond in a reversible manner.^25,46–49^ Inspired by the possibility of using other common, cheap nucleophiles, we investigated the potential of alcohols to form *N,O-*acetals analogously. The abundance of alcohols and polyols from biorenewable origin offers an additional motivation for the potential application of this reactivity.^50^ As a first proof-of-concept, we simply dissolved a model alkylidene oxazolidone (3-allyl-5,5-dimethyl-4-methyleneoxazolidin-2-one, **AllOx**) in dry deuterated methanol. As shown in Figure S1, the two components did not react without a catalyst. However, we observed an instantaneous, exothermic reaction of **AllOx** upon addition of 1 mol % methanesulfonic acid (MSA) as the catalyst. Under these conditions, the reaction was almost quantitative after 15 min of reaction, with conversion reaching 95% as determined by ^1^H NMR spectroscopy (see Figure S1 for details).

Encouraged by this result, we explored the potential of this reaction by selecting a series of alcohols with increasing steric hindrance, from primary to tertiary, comprising phenolic and benzylic moieties. All reactions were carried out in bulk under equimolar conditions (1:1 [**AllOx**]/[R–OH]) in an argon-filled glovebox to avoid possible side reactions with water, as we previously observed that alkylidene oxazolidones hydrated into the corresponding hydroxyoxazolidone under acidic conditions (Figures S2 and S3). When reacted with alcohols, we observed a plateau in the conversion of **AllOx** in less than 1 min in all cases, with a conversion that was strongly influenced by the steric hindrance and nucleophilicity of the alcohol (Figures S4–S11). Most nucleophilic and least sterically hindered primary alcohols displayed the highest conversions, albeit always under 50% (namely, 37, 45.5, and 46.7% for benzyl-, hexyl-, and methyl-alcohol, respectively). Further increases in steric hindrance and lower nucleophilicity brought down the conversion even further, well under 15% (0, 3.9, 8.2, and 14% for *tert*-butyl, phenyl-, 4-methyl-2-pentyl-, and phenyl-ethyl alcohol).

The reaction was pushed to the formation of *N,O-*acetal by adding an excess of alcohol, as demonstrated by >80% conversion with 25 equiv of methanol (Figure S12). Hence, the potential of this strategy for the production of novel oxazolidone scaffolds incorporating novel *N,O-*acetals was explored by synthesizing and characterizing (^1^H NMR, ^13^C NMR, and HRMS) five different model compounds (Figures 2a,b and S14–S18). These molecules were obtained in low to moderate yields (15–60%, additional details in the Supporting Information) after standard organic workup and column chromatography separation without noticeable degradation, serving as an example of a novel library of compounds that could have potential application in pharma, agriculture, and organic synthesis.^51–54^

The equilibrium constant of the reaction was probed by analyzing the kinetics of reaction at temperatures ranging from 25 to 55 °C and using the model **AllOx**/hexanol system in 1:1 ratio (bulk) with 1 mol % MSA (Figures S20–S24). We observed that higher temperatures resulted in lower C=C conversions at equilibrium. Indeed, a Van’t Hoff plot of the equilibrium constant, *K*_eq_, obtained at different temperatures provided thermodynamic parameters of Δ*H* = −10.7 kJ·mol^–1^ and Δ*S* = −8.9 J·K^–1^ mol^–1^ (Figure 2c), consistent with an exergonic process.

**Figure 2:**
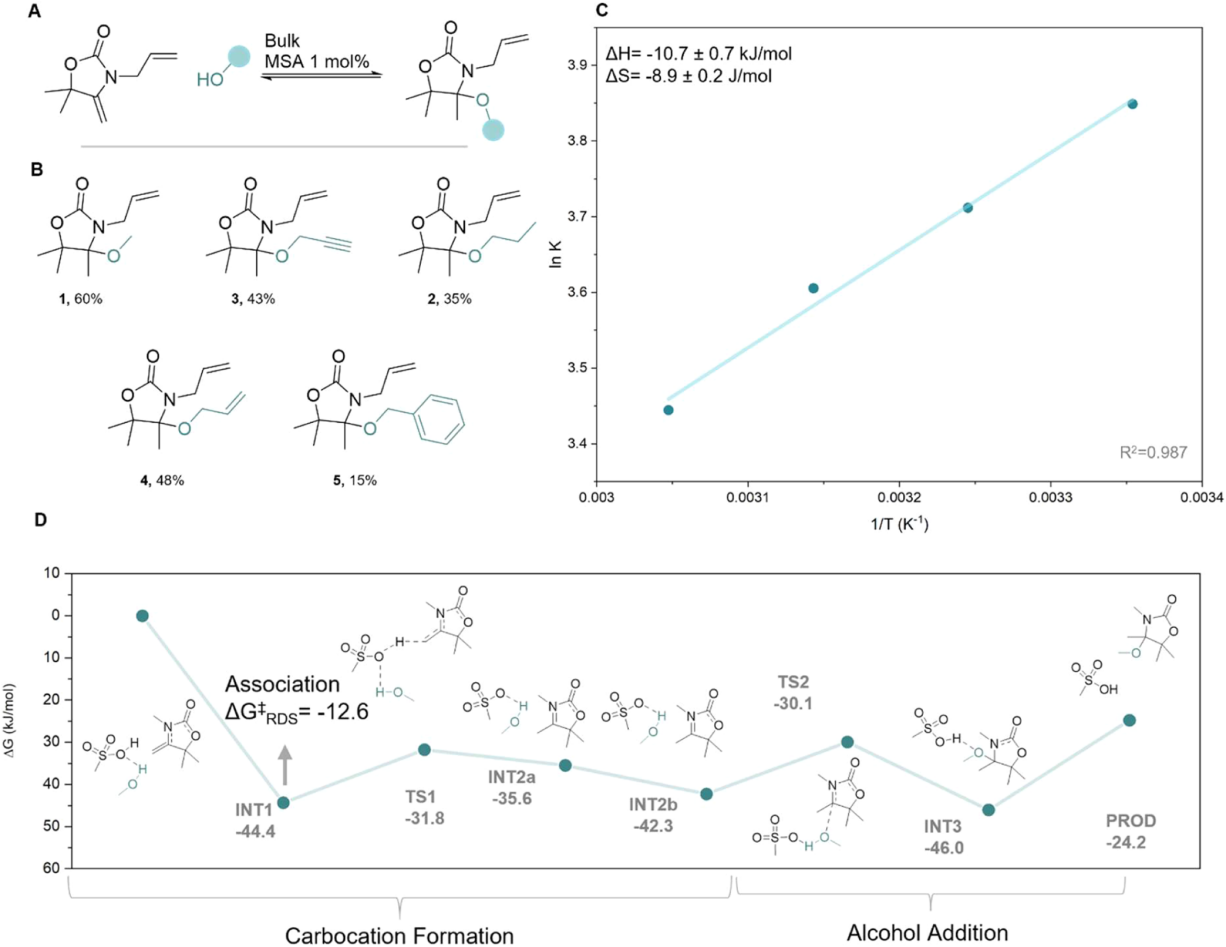
(A) General reaction scheme between **AllOx** and an alcoholic partner (1:1 molar ratio); (B) scope of *N,O-*acetal derivatives with isolated yields; (C) Van’t Hoff graph extracted from kinetics carried out at different temperatures (bulk, equimolar ratio) with corresponding Δ*H* (−10.7 kJ/mol) and Δ*S* (−8.9 J/mol) of reaction; and (D) Gibbs free energy profile of the reaction pathway for the formation of the *N,O-*acetal moiety between a model oxazolidone and a model alcohol (methanol) catalyzed by MSA. Vertical arrows show the energy barriers for rate-determining steps.

In order to further understand the reaction dynamics, we performed density functional theory^55,56^ calculations on the model reaction between **AllOx** and methanol using the long-range corrected ωB97XD^57^ functional with the 6–31+G­(d)^58^ basis set for geometry optimizations and the 6–311++G­(2df,2p)^59^ basis set for the electronic refinement. All calculations were carried out using the Gaussian16 package.^60^ A conductor-like polarizable continuum solvation model (ε = 4.7113, chloroform) was employed, and the substituents were replaced by methyl groups mimicking the aliphatic chains of the synthesized model compounds to enable faster calculations (Figures 2d and S25). Similar to previously reported mechanisms,^25^ the starting reactant complex is stabilized by hydrogen bonding and the interaction between the acidic MSA and the electron-rich alkene (Δ*G* = −44.4 kJ/mol). The delocalization of the charge through the oxazolidone ring further stabilizes the ring. Next, the rate-determining step (RDS) has a very low activation barrier of 12.6 kJ/mol, pointing toward the reason behind the quick reaction dynamics in all experimental conditions explored. In this step, the acidic proton of the MSA is donated to the oxazolidone ring, forming a delocalized carbocation. The then-formed intermediate **2a** is characterized by the formation of a new π bond between the nitrogen and the carbocation, leading to a stabilization of the species (−42.3 kJ/mol) and a shortening of the N–C bond (from 1.320 to 1.292 Å, Table S1). A minor reorganization leads to intermediate **2b**, and then the carbocation is attacked by the nucleophilic hydroxyl with an energy barrier of 12.1 kJ/mol leading to the formation of transition state 2 and breaking the π bond, signaled by the lengthening of the N–C bond (1.292–1.318 Å, Table S1). In a concerted step, the hydrogen of the hydroxyl is transferred to the catalyst, regenerating it (intermediate **3**, −46.0 kJ/mol). The little stabilization of this intermediate with respect to the other reaction steps explains the reaction’s tendency to plateau at 46.7% conversion as the product is not strongly stabilized (−24.8 kJ/mol) in contrast to the starting reactants. This lower stabilization also explains the lower efficiency of this reaction compared to its thiol counterpart (Δ*G*_*N,O*‑acetal_ = −24.8 kJ/mol, Δ*G*_*N,S*‑acetal_ = −73.2 kJ/mol, Figure S26 for further explanation).^25^ In summary, DFT calculations explain the behavior of the reaction. Low stabilization energy and small Δ*G*_RDS_^⧧^ led to quick reaction with low conversions, as observed experimentally.

The thermal reversibility of the process was investigated by *in situ* NMR spectroscopy, in which a solution of *N,O-*acetal **4** in dry DMSO-*d*_6_ was equilibrated at various temperatures (25–100 °C for 15 min) and subsequently their ^1^H NMR spectra were recorded. While no reaction was observed in a neutral environment or in the presence of a base (1 mol % triethylamine, TEA, Figure S27), the addition of acidic MSA (1 mol %) triggered the dissociation of the model compound into free **AllOx** and allyl alcohol, even at room temperature (4% of dissociation). Elevating the temperature increased the dissociation to reach 86% at 100 °C (Figures S28–S30), further corroborating the role of protic species in the mechanism of association/dissociation of these *N,O-*acetals.^61,62^

To gain further insight into this mechanism, we probed the reaction kinetic barrier of exchange between alcohols by determining the activation energy. Thus, a 0.2 M solution of **4** and a 10-fold excess of MeOH (Figure 3a) were mixed with MSA (1 mol %) in dry CDCl_3_ and the exchange reaction was monitored. Unfortunately, this resulted in a reaction that was too quick to be monitored (completed below 1 min), even with 0.1 mol % MSA. Hence, we employed a weaker acid (trifluoracetic acid, TFA) as we previously saw that it showed reduced reaction rates (Figure S31). The extent of the alcohol exchange at equilibrium was found to be strongly influenced by temperature, increasing from 8 to 40% on going from 25 to 55 °C (Figures 3b and S32–36), which led to a calculated activation energy (*E*_a_) of 38.87 ± 0.4 kJ/mol (Figure 3b and Table S2). This result confirmed that the covalent exchange dynamics were quick in the presence of protic species, with a lower *E*_a_ than previously reported *N,S-*acetal compounds (64.85 kJ/mol).^25^

**Figure 3:**
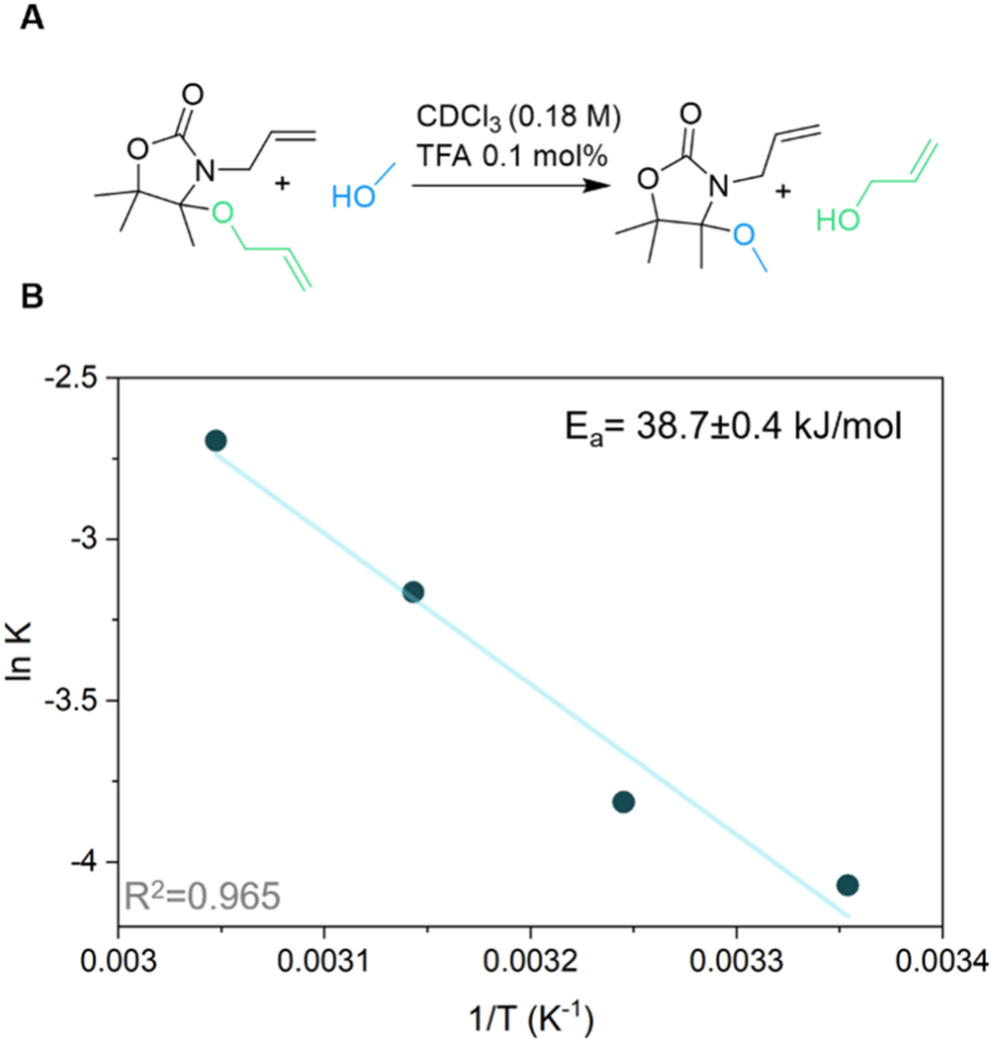
(A) Schematic of the model exchange reaction. Reaction conditions: [**4**]/[methanol] = 1/10, CDCl_3_ (0.18M), 0.1 mol % TFA; (B) Arrhenius plot extracted from exchange kinetics carried out at temperatures between 25 and 55 °C.

### Preparation of Photocurable Materials

We decided to incorporate the *N,O-*acetal functionality in a polymeric matrix, since this was expected to endow the network with both recyclability and degradability. Monomer **4** was selected for the preparation of the material as it already possesses two photocurable allyl bonds that can participate in step-growth polymerization via radical thiol–ene reaction with polythiols.^63^ In order to do so, **4** was mixed with a tetrafunctional thiol cross-linker; pentaerythritol tetrakis­(3-mercaptopropionate), **S4**. Finally, we chose phenylbis­(2,4,6-trimethylbenzoyl)­phosphine oxide (BAPO) as the radical photoinitiator (0.5 wt %), and thin films of the liquid, clear mixture were casted and irradiated with 390 nm light (20 mW·cm^–2^) for 60 s.

Surprisingly, a transparent and soft material with a sticky surface was obtained after irradiation rather than as a self-standing object (Figure 4a). Analysis of this material by Fourier transform infrared (FT-IR) revealed that, in addition to the consumption of the allyl C=C (1644 cm^–1^), an intense −OH stretching band (Figure 4b) together with a shoulder (1682 cm^–1^) of the carbonyl stretching peak (1729 cm^–1^) appeared, indicative of the presence of free exovinylene double bonds (Figure S13).^49^ These results indicated that the reverse reaction (elimination of the alcohol from the oxazolidone ring) was being catalyzed under these photoreaction conditions. To gain a better insight on this reaction, a mixture of **4** and a monofunctional thiol similar to our cross-linker (methyl-3-mercaptopropionate) was reacted and the reaction was followed by ^1^H NMR spectroscopy. After 60 s at room temperature without any irradiation, 12 mol % **AllOx** was liberated, as indicated by the presence of new resonances at 5.21 ppm (dd, C=CH_2_) and 1.46 pm (singlet −CH_3_) (Figure S37a). We attributed this reactivity to the high sensitivity of *N,O-*acetal to acidic species, including weakly acidic thiols, in promoting the release of the alcohol unit and the formation of the exovinylic C=C. As a further proof of the role of acidic species in catalyzing the liberation of AllOx from **4**, the use of basic triethylamine (TEA) as an additive indeed prevented any side reaction between **4** and methyl-3-mercaptopropionate and provided quick reaction kinetics (Figures S37b and S38). Furthermore, we could confirm that no *N,S*-acetal adducts were formed in the reaction conditions as no singlet at 1.57 ppm (−CH_3_ *N,S*-acetal) nor multiplet at 1.91 ppm (−CH_2_– *N,S*-acetal) were observed (Figure S39)

**Figure 4:**
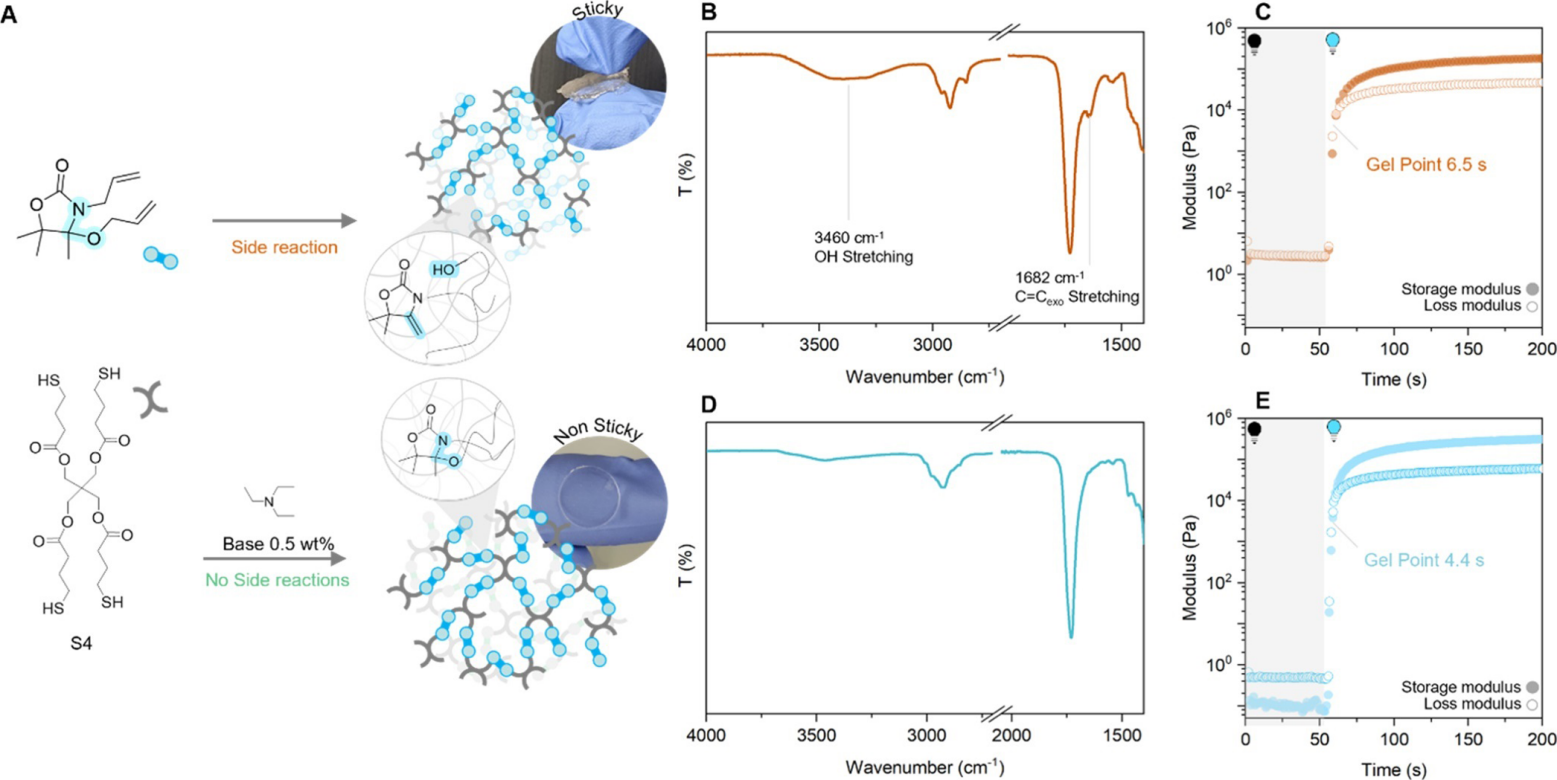
(A) Schematic of the preparation of materials containing *N,O-*acetal moieties with and without TEA; (B) IR spectra of the material **P­(S4,100)** without addition of a TEA (BAPO 0.5 wt %, 390 nm, 20 mW/cm^2^, 60 s); (C) photorheology results for **P­(S4,100)** without TEA; (D) IR spectra of the material **P­(S4,100)** using TEA (TEA 0.5 wt %, BAPO 0.5 wt %, 390 nm, 20 mW/cm^2^, 60 s); and (E) photorheology results for **P­(S4,100)** with TEA (TEA, 0.5 wt %).

Satisfyingly, using 0.5 wt % TEA as an additive enabled the photocuring of the resin in a rapid manner, resulting in a fully cross-linked transparent, self-standing film after only 60 s of irradiation. The FT-IR spectra showed complete consumption of the double bond resonance (1644 cm^–1^, Figure 4d; 3083 cm^–1^, Figure S40) together with a markedly lower intensity of the −OH resonance (3460 cm^–1^, Figure 4d). The successful preparation of a fully cross-linked matrix was further supported by high gel contents in THF (95 ± 2%) and low swelling degrees (135 ± 2%, Table S3, entry 12). Photorheology showed a fast gel point (4.4 s), and real-time FT-IR (Figure S40) highlighted quick curing with the double bond band (1644, 3083 cm^–1^) being fully consumed after 20 s (390 nm, 20 mW/cm^2^). Photorheology further supported the occurrence of side reactions in the absence of the basic additive with a final modulus that was found to be lower than the resin prepared in the presence of the base additive (0.19 MPa without basic additive, 0.33 MPa using TEA, Figure 4c,e). Hence, from this point, all materials were prepared using TEA (0.5 wt %) as the additive.

**Figure 5:**
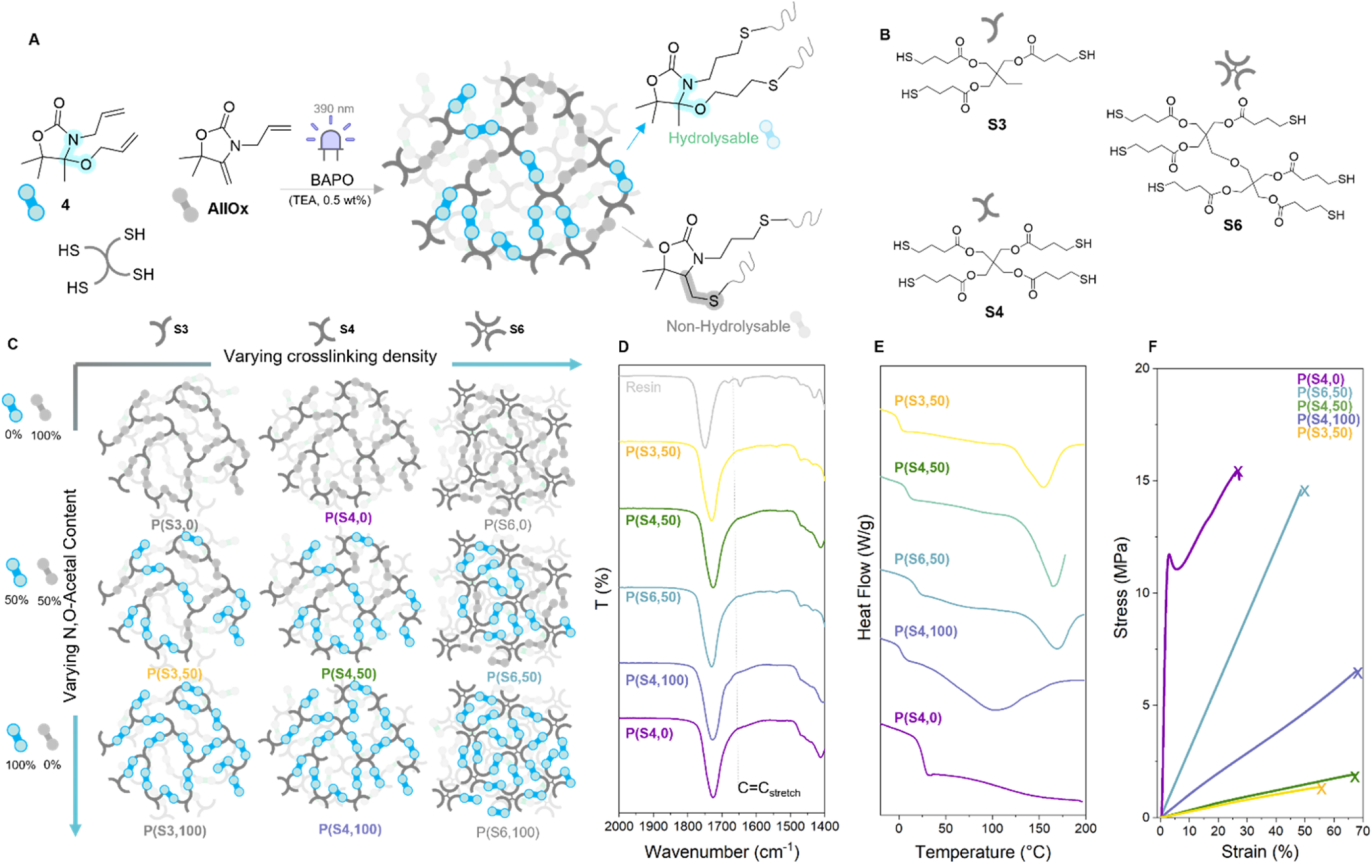
(A) Schematic of the preparation of material mixing **4** and AllOx to insert varying amounts of cleavable bonds in the network structure (BAPO 0.5 wt %, TEA 0.5 wt %, 390 nm, 20 mW/cm^2^, 60 s); (B) structures of the thiol cross-linkers used to vary the cross-linking density of the materials; (C) schematic of the preparation of materials with varying cross-linking densities and varying contents of *N,O-*acetal moieties; (D) IR spectra of the resin and material showing effective curing; (E) DSC traces of materials prepared in this study; and (F) stress–strain curves of the materials prepared in this study (Young’s modulus, elongation at break, and stress at break data are summarized in Table S3).

### Versatility of the *N,O*-Acetal Chemistry

With a reliable photo-cross-linking platform, we tuned the properties of the materials by changing the concentration of cleavable functionalities within the polymer network. Given the ability of **AllOx** to participate efficiently in the radical thiol–ene reaction,^64^ we mixed **AllOx** and **4** in a 1:1 ratio. By doing so, we could prepare thermosets featuring a combination of hydrolyzable (*N,O-*acetal) and nonhydrolyzable (thioether linkages) bonds, resulting in a convenient platform to study the influence of the structural connectivity on the material’s properties and hydrolytic degradation (Figure 5a). For comparison, mixtures of only **AllOx** and thiol cross-linkers were also prepared, providing only nonhydrolyzable thiol-ether cross-links. To further tune the properties, materials with varying cross-linking densities were prepared (Figure 5b,c). In order to do so, **4** was mixed with a multifunctional thiol cross-linker trifunctional trimethylolpropane tris­(3-mercaptopropionate), **S3**, tetrafunctional pentaerythritol tetrakis­(3-mercaptopropionate), **S4**, or hexafunctional dipentaerythritol hexakis­(3-mercaptopropionate), **S6** (Figure 5). Hence, the materials were labeled **P­(X,Y)**, where **X** denotes the thiol cross-linker (S3, S4, or S6) and **Y** represents mol % **4** in the mixture of **4** and **AllOx** (0, 50, or 100%) (Figure 5c). Finally, we chose phenylbis­(2,4,6-trimethylbenzoyl)­phosphine oxide (BAPO) as the radical photoinitiator (0.5 wt %), and thin films of the blends were casted and irradiated with 390 nm light (20 mW·cm^–2^) for 60 s. All materials were prepared using TEA (0.5 wt %) as an additive to avoid side reactions during photocuring.

Gratifyingly, all materials showed efficient curing with a steep reduction of the double bond stretching resonance at 1644 cm^–1^ (Figure 5d). Thermal characterization of the materials by differential scanning calorimetry (DSC) revealed two important characteristics. First, a clear trend of raising *T*_g_ with increased cross-linking density, as demonstrated by increasing the thiol functionality on going from **P­(S3,50)**, **P­(S4,50)**, to **P­(S6,50)** and attaining *T*_g_’s of −0.2, 2, and 18 °C respectively (Figure 5d and Table S3, entries 5, 7, 8). The same trend was followed by materials using 100% **4** (**P­(S3,100)** *T*_g_ = 1.4 °C, **P­(S4,100)** *T*_g_ = 8.8 °C, **P­(S6,100)** *T*_g_ = 19.5 °C, Table S3, entries 11–13). Second, an endothermic peak was observed for all networks containing **4** attributed to the cleavage of the *N,O-*acetal linkages. While no protic species were deliberately added that could have catalyzed the network dissociation reaction, the protic character of the photodegradation products of BAPO (phosphonic acid-like fragments) could not be ruled out.^65^ To support this possibility, we mixed **4** with the photodegradation products of BAPO (dissolved in DCM and irradiated for 30 min at 390 nm, 20 mW/cm^2^). After 15 min of reaction, we observed the characteristic signals of **AllOx**, proving that indeed the cleavage of the *N,O-*acetal linkages was catalyzed by the photodegradation products of BAPO (Figure S41).

Further support of the ability to depolymerize the materials came from dynamic mechanical analysis (DMA), variable temperature in situ FT-IR traces, and reprocessing studies. Comparing the traces of two networks, **P­(S4,50)** and **P­(S4,100)**, during a heating ramp of a DMA test, we observed a lower onset of the depolymerization reaction in the network with the highest number of reversible cross-links (from 139 to 117 °C, respectively, Figure S42), in accordance with DSC analyses (Table S3). The depolymerization could also be observed in situ in the FT-IR traces of both **P­(S4, 100)** and **P­(S4,50)** on heating the sample from 25 to 150 °C with the increase in the band of the exovinylene C=C double bond (1682 cm^–1^, Figure S43a,b), consistently with the rupture of the *N,O-*acetal bond that released **AllOx**. Notable, the sample containing only 50% of *N*,*O*-acetal bonds embedded in the network showed a lower intensity of this resonance, coherently with its lower content in dynamic bonds. The ability to reprocess **P­(S4,100)** was further confirmed by hot-pressing and molding various fragments. Upon applying 1 ton of pressure at 90 °C for 5 min, a transparent film was obtained with similar FT-IR spectra and *T*_g_ (7.9 °C) to the virgin material (*T*_g_ = 8.8 °C, Figure S44). Its storage moduli lowered from *E*′_virgin_ at −10 °C = 2549 MPa to *E*′_reprocessed_ at −10 °C = 1926 MPa, showing that the material only partially recovered its mechanical properties after reprocessing.

Next, we studied the mechanical properties of the materials. As expected, increasing the cross-linking density increased the Young’s modulus, ranging from 3.0 ± 0.2 MPa (**P­(S3,50)**) to 27.4 ± 1.5 MPa (**P­(S6,50)**, Figure 5f and Table S3, entries 5, 8). Together with this marked stiffening, the stress at break rose as well, from 1.3 to 11.4 MPa. On the other hand, the elongation at break remained within a similar order of magnitude, at 45.5 ± 1.5 and 47.2 ± 2.3% for **P­(S3,50)** and **P­(S6,50)**, respectively. While a clear trend was seen when varying the cross-linking density, materials derived from mixing **AllOx** and **4** gave starkly different materials. **P­(S4,0)** showed a clear yield point at 4.5% elongation with a modulus of 737 MPa followed by plastic deformation until 25% elongation and a stress at break of 11.9 MPa (Figure 5f and Table S3, entry 2). The material made by mixing **AllOx** and **4** in a 1:1 ratio was much softer, with a modulus of 2.8 MPa and a stress at break of 1.77 MPa. Curiously, **P­(S4,100)** was stronger, with a 3-fold increase in modulus (10.4 MPa) as well as stress at break (5.97 MPa). This was attributed by the fact that allyl bonds react much faster in the thiol–ene reaction when compared to the electron-rich exovinylene bond.^66–68^ Thus, when photocuring, the allyl bond reacted quicker, raising the viscosity and hindering the thiol–ene reaction on the exovinylene double bond. We hypothesize that this leads to a network with more defects.

### Hydrolytic Degradation: From Model Compounds to Materials

Next, we hypothesized that the structural differences between *N*,*S*- and *N,O-*acetals derived from oxazolidones, which give markedly different exchange dynamics in the bulk when catalyzed by acids (*vide supra*), would also result in distinct reactivities toward hydrolysis. Indeed, as with many other acid-cleavable bonds (imines, hydrazones, acetals, ketals, orthoesters, etc.),^40,43^ the choice of the heteroatom and substituent plays a key role in the pH sensitivity to hydrolytic cleavage, and thus in the hydrolysis rate constants.^40^ When installed in a polymer network of the right topology, hydrolysis of the *N*,*S*- or *N,O-*acetal bond would then offer a chemical handle for the disassembly of the polymer structure, leading to its possible (bio)­degradation.

To assess the hydrolysis of our newly synthesized model *N,O-*acetals, we investigated the reaction of **4** in H_2_O (0.2 M) under neutral or acidic conditions (1 mol % MSA), and we compared it with the one of the *N,S-*acetal counterpart (Figure 6a). As expected, the reaction rate and degree of hydrolysis were markedly different between the two compounds (Figure S45). Specifically, in neutral environment, 24% hydrolysis of **4** was reached after 4 days, while it only required 30 min to hydrolyze quantitatively in acidic media (Figures S45–S47). On the other hand, *N,S-*acetal model **6** displayed much higher resistance to hydrolysis, with no hydrolysis products observed after 4 days under neutral conditions, and reaching only 24% in acidic media (1 mol % MSA, Figures S45, S48–S49). In comparison, a classic *O*,*O*-acetal (1,1-dimethoxyethane) degraded to methanol and acetaldehyde much more readily than the *N*,*O*- or *N,S-*acetal (albeit through a slightly different set of reaction intermediates), reaching full or near-full conversions in less than 1 or 24 h, in acidic or neutral conditions, respectively (Figures S50–S52). These results suggested the potential of *N,O-*acetals based on oxazolidone building blocks to control the degradation of polymer materials.^40^ To clarify the mechanism of hydrolysis, we modeled the reaction of the *N,O*-acetal methanol adduct with water in the presence of MSA by DFT. The reaction was modeled in a conductor-like polarizable continuum solvation model (ε = 4.7113, chloroform). It was found to be composed of two transition states and one intermediate. The RDS, being the formation of TS1 (Δ*G*_RDS_ = 24.6 kJ/mol), was the exit of the alcoholic group to form a carbocation that was strongly stabilized by H bonding of water, methanol, and MSA (−11.1 kJ/mol, INT1) (Figures S53, S54 and Table S4). Subsequent addition of water (Δ*G*_association_ = 18.9 kJ/mol) gave the hydrated product, with a stabilization of −8.8 kJ/mol highlighting the 2-step nature of the mechanism. Furthermore, the higher stabilization of the hydrated product motivated the efficient hydrolysis of the *N,O*-acetal adduct.

**Figure 6:**
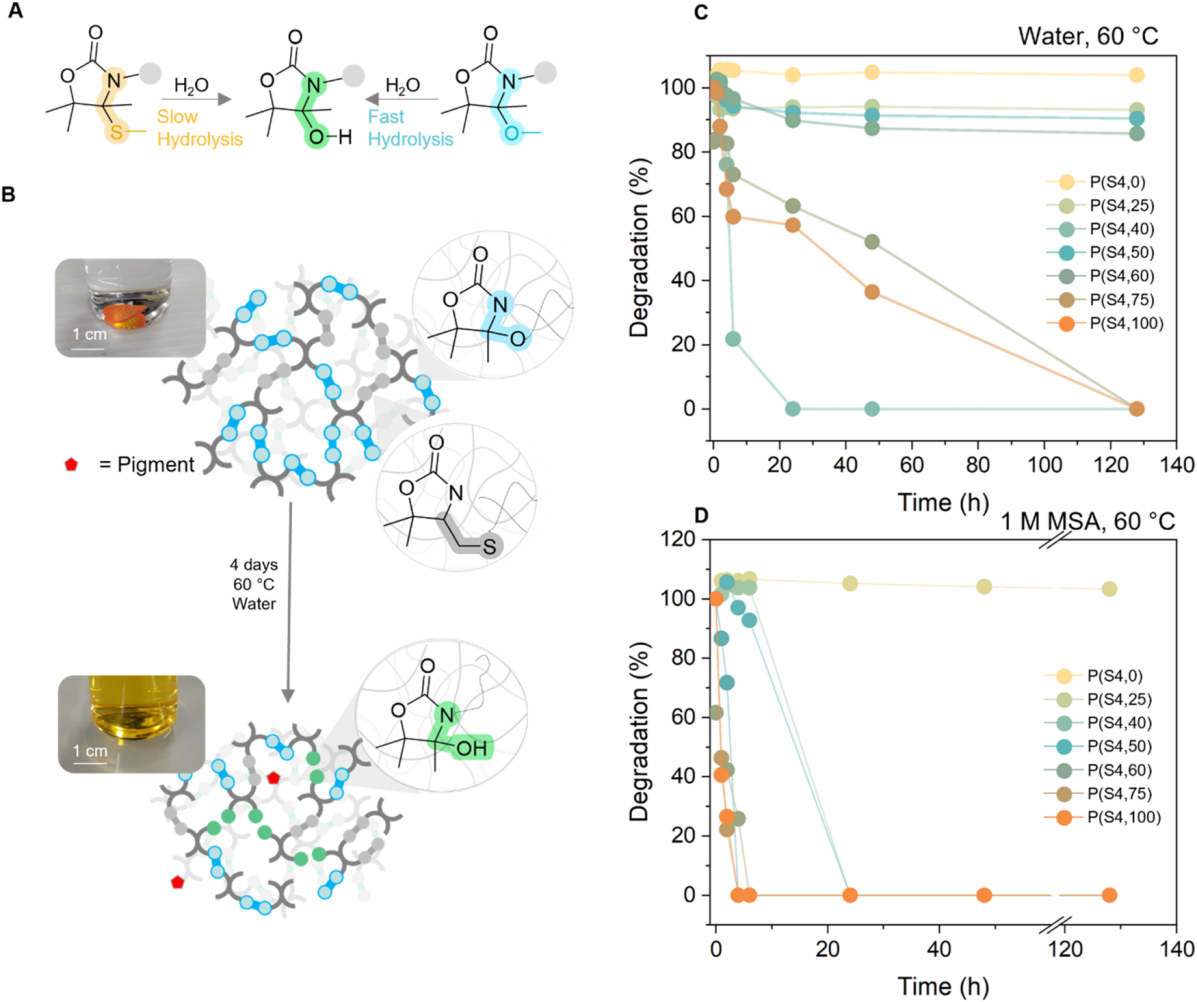
(A) Schematic of the hydrolytic degradation of *N,S-*acetal and *N,O-*acetal moiety; (B) schematic of the hydrolytic degradation of a material containing both *N,O-*acetal moieties and thioether bonds. Degradation of materials with 0 to 100% content in *N,O-*acetal linkages (**P­(S4,0)** to **P­(S4,100)**) under neutral conditions (water, 60 °C) (C) and acidic conditions (1 M MSA) at 60 °C (D).

We decided to investigate the hydrolytic degradation rate of our thermoset materials in water in the presence of MSA (1.0 M MSA; Figure 6b). To increase the range of materials with tunable degradability, we prepared four new materials with 25, 40, 60, and 75% of cleavable bonds (Table S3). Together with the previous materials prepared with 0, 50, and 100% cleavable bonds, a large set of cross-linked materials with controlled degradability was thus available for this study. As can be seen in Figure 6b,c, the material composed of solely thioether functionalities did not degrade at 25 and 60 °C, in neutral, or in acidic media (Figure S55a,b). When exposed to higher temperatures (100 °C), the material was degraded in acidic environment, possibly by hydrolysis of the ester functionalities present in the tetrathiol cross-linker (Figure S55c,d). On the contrary, about 15 and 85% of the material containing exclusively *N,O-*acetal bonds degraded at 25 °C in neutral and acidic environments after 4 days, respectively. Higher temperature (60 °C) led to an acceleration of the process, with complete degradation in minutes in an acidic environment and complete degradation in 4 days in a neutral environment (Figure 6c,d). At 100 °C, the material was degraded below 5 min in both acidic and neutral environments (Figure S55c,d). While the material comprising 25:75 and 40:60 *N,O-*acetal to thioether ratios behaved as expected, with faster degradation than the material with 0% *N,O-*acetals and slower than the material with 100% *N*,*O*-acetals, the materials with 50, 60, and 75% *N,O-*acetals were found to degrade faster than the material with 100% *N,O-*acetals (Figures 6c,d S55, and S56). This was attributed to the lower cross-linking density of this material as discussed above. The ability to control the speed and efficiency of the material’s degradation shows the potential for these materials to be used as cargo delivery and their hydrolytic degradation.

### 3D Printing

Taking advantage of the fast-photocuring behavior of the resins, we studied the printability of **P­(S4,100)**. We analyzed the photocuring behavior of the resin using a Jacobs curve, a commonly used technique in the vat photopolymerization field to estimate the degree of light penetration and critical energy of a given resin.^69^ The resin behaved well, with low critical energy and good light penetration (Figure 7a), being able to form a 50 μm layer in 2.5 s of exposure. 2.5D structures bearing grooves of 500, 250, and 100 μm could be prepared by irradiating each layer for 2.5 s. The printed lines exhibited sharp definition and achieved a resolution with less than 10% deviation from the expected feature size (Figures 7b,c and S57). A 3D printed gyroid (Figure 7d,e,f) cube could be vat-3D printed using **P­(S4,100)** as the formulation resin, proving the printability of this kind of material. While the XY resolution was found to be satisfactory, the Z resolution should be optimized by using commonly available light absorbers.^70,71^ **P­(S4,50)** behaved as well in 3D printing, albeit with lower resolution (Figures S57 and S58a,b) than **P­(S4,100)**. Furthermore, it showed similar degree of penetration and critical energy to **P­(S4,100)** (Figure S58c,d,e), demonstrating the potential to print structures with tunable degradation.

**Figure 7:**
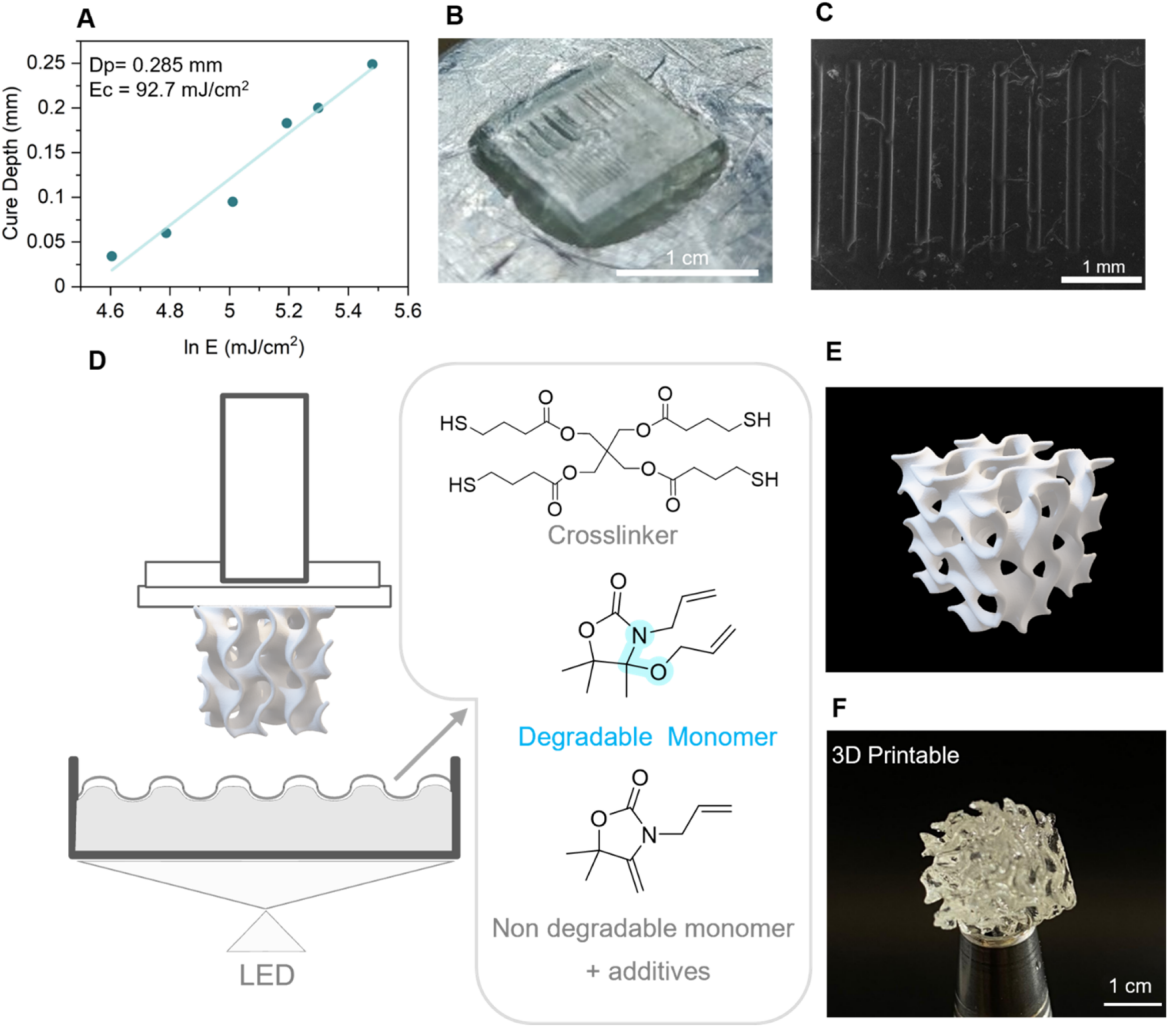
(A) Jacobs curve for P­(S4,100) (390 nm, 20 mW/cm^2^) (D) schematic of the DLP printer; (B) 3D printed structure for resolution optimization (390 nm, 20 mW/cm^2^); (C) SEM images of 100 μm lines; (d) schematic of the 3D printer and resin formulation; (E) 3D structure of a gyroid cube; (F) 3D printed gyroid cube manufactured using a MAX UV asiga DLP printer (390 nm, 20 mW/cm^2^).

## Conclusions

In this study, we presented a new type of CO_2_-derived CAN that enabled the introduction of hydrolytic degradation in oxazolidone-based materials and their manufacturing through 3D printing. We first characterized the kinetics of bond formation via ^1^H NMR and found quick reactivity, with the reaction plateauing in under 1 min although with low to medium conversions (below 50%). The conversion was found to be mainly influenced by the steric hindrance of the alcoholic partner and was increased by adding excess alcohol. DFT supported the experimental results, highlighting a low stabilization of the product compared to the starting materials (−50.2 kJ/mol) and small Δ*G*_RDS_^⧧^ (−12.6 kJ/mol), attesting for the fast kinetics. The dynamic behavior of the bond was then clarified to be dissociative with an activation energy of 38.9 kJ/mol. With this knowledge in mind, the *N,O-*acetal bond was installed in a series of materials with fast-photocuring (curing time below 60 s), enabling their 3D printing. We studied their thermal and mechanical properties, finding *T*_g_ ranging from −0.2 to 19.5 °C and Young’s Moduli from 2.8 to 27.4 MPa. Exploiting the dissociative character of the *N*,*O*-acetal bond, we recycled the material, with retention of similar IR spectra and *T*_g_ value (*T*_g,virgin_ = 8.8 °C, *T*_g,reprocessed_ = 7.9 °C). However, we observed a slight loss in mechanical properties (*E*′_virgin_ = 2549 MPa, E′_reprocessed_ = 1926 MPa). A key property of *N,O-*acetal, when compared to previously reported CO_2_-derived CANs, is its hydrolytic degradability feature. We studied this behavior using both small molecules and DFT modeling. We could tune the degradation rate in cross-linked materials by changing the ratio of the hydrolyzable moiety in their composition, promising applications in cargo delivery and degradable polymers. Importantly, this chemistry also shows high potential for the incorporation of polyols derived from biorefineries, aligning with growing interest in renewable feedstocks and offering an attractive route toward more sustainable and circular material design. Further work is now focusing on improving the recyclability and accessibility of this type of materials.

## Supplementary Material



## Data Availability

All data are available in the main text or the Supporting Information.

## Notes

Materials, instrumentation, synthetic procedures, ^1^H and ^13^C NMR spectra, HRMS-ESI measurements, DFT computational details and 3D structures, kinetic experimental procedures and plots, IR spectra, DSC thermograms, DMA analysis, tensile test characterization, gel content measurements, rheological data including photorheology, 3D printing conditions including the Jacobs curve, and accelerated degradation studies (PDF)
