# Supplementary material for: A Material Platform Based on Dissociative CO2‑Derived N,O-Acetals for Tunable Degradation of 3D Printable Materials
Source: J Am Chem Soc. 2025 Aug 7;147(33):30095–106. doi: 10.1021/jacs.5c07767 (PMC12371883; doi:10.1021/jacs.5c07767)
Supplement: Supplementary file 1 [file ja5c07767_si_001.pdf]

Supporting Information

A Material Platform based on Dissociative CO_2-derived *N,O*-acetal*s* for Tuneable Degradation of 3D Printable Materials

Marco Caliari,[a,b] *Jacopo Teotonico,*[a] *Mikel Irigoyen,*[a] *Anje Mujika,*[a] *Tommaso Isolabella,*[c] *Daniele Mantione,*[a,d] *Lourdes Irusta,*[a] *Bruno Grignard,*[b,e] *Fernando Vidal,*[a*] *Christophe Detrembleur*[b,f*] *and Haritz Sardon*[a*].

[a]POLYMAT and Department of Polymers and Advanced Materials: Physics, Chemistry and Technology, Faculty of Chemistry, University of the Basque Country UPV/EHU, Paseo Manuel de Lardizábal, 3, 20018 Donostia-San Sebastián, Spain. [b] Center for Education and Research on Macromolecules (CERM), CESAM Research Unit, University of Liege, Sart-Tilman B6a, 4000 Liege, Belgium. [c]INFN and Department of Physics, University of Genoa, via Dodecaneso 33, 16146 Genoa [d]Ikerbasque, Basque Foundation for Science, Plaza Euskadi 5, 48009, Bilbao. [e] FRITCO$_2$T Platform, University of Liege, Sart-Tilman B6a, 4000 Liege, Belgium.[f] WEL Research Institute, Avenue Pasteur 6, 1300 Wavre, Belgium.

S1. Materials and Methods

S1.1 Materials

2-Methyl-3-butyn-2-ol (Sigma, 98%), 4-methyl-2-pentanol (>99%, Sigma), Allylamine (Sigma, 98%), Copper Iodide (Sigma, 98%), Methyl 3-mercaptopropionate (Sigma, 98%), Phenylbis(2,4,6-trimethylbenzoyl) phosphine oxide (BAPO, Ciba), Methanol (HPLC grade, Sigma), Allyl Alcohol (anhydrous, >99%, Sigma), Benzyl Alcohol (Anhydrous, >99%, Sigma), 1-Propanol (Anhydrous, >99%, Sigma), 1-Hexanol (Anhydrous, >99%, Sigma), Methanesulfonic acid (Sigma, >99%), Trimethylolpropane tris(3-mercaptopropionate) (S3, Sigma, ≥95%), Pentaerythritol tetrakis(3-mercaptopropionate) (S4, Sigma, ≥95%), Dipentaerythritol Hexakis(3-mercaptopropionate) (S6, TCI, >93%), and Sulfuric Acid (Sigma, >95%), Triethylamine (TEA, Sigma, 99.5%), 1,8-Diazabiciclo[5.4.0]undec-7-eno (DBU, Sigma, 98%) were used without further purification

S1.2 Methods

Nuclear magnetic resonance (NMR) spectroscopy. ^{1}H- and ^{13}C-NMR analyses were performed on a Bruker Avance 300 MHz spectrometer at 25 °C in the Fourier transform mode using $CDCl_3$ or DMSO-d_6 as solvents**.** High temperature 1H NMR experiments were carried out on a Bruker Avance 500 MHz.

HRMS (ESI) data were acquired in SCAN mode, using a mass range 50–1000 u in resolution mode (FWHM ≈ 20,000) and a scan time of 0.1 s. The source temperature was set to 120 °C and the desolvation temperature to 350 °C. The capillary voltage was 0.7 kV and the cone voltage 15 V. Nitrogen was used as the desolvation and cone gas at flow rates of 600 L/h and 10 L/h, respectively. Before analysis, the mass spectrometer was calibrated with a sodium formate solution. A leucine-enkephalin solution was used for the lock mass correction, monitoring the ions at mass-to-charge ratio (m/z) 556.2771 and 278.1141. All of the acquired spectra were automatically corrected during acquisition based on the lock mass. The samples were dissolved in the corresponding solvent at a concentration of 1 mg/ml and diluted to 20 µg/mL for the analysis.

Gel content (GC). Films prepared as described in the experimental section were cut in rectangular shapes (~120 mg) and weighted (m_1) before immersing 48 h in tetrahydrofuran. After weighing, the swelled films (m_2) were dried in a vacuum oven at 60 °C for 24 h. The

films were weighed once again (m_3). Swelling (SI) and Gel content (GC) were calculated with equation 2 and 3, respectively:

$$SI = 100 * {}^{m_2}/_{m_1} (2)$$

$$GC = 100 * {}^{m_3}/_{m_1} (3)$$

Tensile tests were carried out on dogbone samples (see Preparation of Films below) (ASTM D638 TYPE V) in an Instron 5569 tensile tester (Instron, Norwood, MA, USA). Young's modulus, Tensile strength (σ_t), Yield strength (σ_y), and strain at break (ε_b) were determined using Bluehill software from the load-displacement curves at a crosshead speed of 10 mm/min. A minimum of three tensile specimens were tested for each reported value**.**

Fourier Transform Infrared Spectroscopy (FT-IR) spectra were recorded on a Nicolet iS20 Spectrometer using Attenuated Total Reflection (ATR) at a resolution of 4 cm^{-1} and a total of 32 interferograms. The same instrument was used to acquire real/time FTIR data. Spectra were acquired every 5 seconds by averaging 4 spectra. The spectra at high temperature were obtained on a Nicolet 6700FT-IR spectrophotometer equipped with a specap variable temperature transmission cell. Spectra were recorded in the range of 4000 and 400 cm^{-1} with a spectrum resolution of 4 cm^{-1}, and a total of 64 interferograms.

Photorheology was performed on an ARG2. A LED UV-curing accessory centered at 365 nm (20 mW/cm^2) was used together with an acrylic transparent bottom plate and a single use aluminum plate (20 mm). The measurement was performed in the linear viscoelastic regimen with frequency of 1 Hz with a gap of 400 µm. To determine the viscosity of the formulated resins, a 1° 40 mm cone geometry was used. The shear rate window was from 1 to 100 s^{-1}.

Jacobs Curve were obtained by placing a drop of resin on a glass coverslip and irradiated directly from below at different exposure times with a 2 mm circular beam using the spot timer of the ASIGA MAX-UV DLP 3D printer centered at 385 nm, giving an irradiance of ~20 mW/cm2 . The thickness of the photocured resin was then measured with an Evanem Dloett electronic micrometer and the depth of cure was plotted against the logarithm of the light intensity for irradiation time.[1]

DLP Printing A commercial Asiga Max-UV DLP 3D printer with a LED source centered at 385 nm (20 mW/cm^2) was used to 3D print the resin.The 3D objects were sliced on the Asiga composer with 6.5 s burn-in layer, 2.5 s exposure time and 50 µm layer thickness at an intensity of 20 mW/cm2. The printed objects were washed thoroughly with Isopropanol to remove unreacted monomers and postcured using a low-pressure mercury vapor lamp (2 mW/cm^2, centered around 365 nm). 3D models were obtained from Thingiverse (https://www.thingiverse.com/). XY resolution was characterised by 3D printing structures possessing features of known size and imaging them using SEM. ImageJ was used to measure the features and the ratio between the expected size and the observed one was plotted using Origin.

Differential Scanning Calorimetry was performed on a TA Instruments DSC 250 calibrated with indium, and the curves were analyzed using the Trios software. A ramp of 10 °C/min from -20 to 200 was used.

Dynamic Mechanical Analysis (DMA) was performed on a DMA Q800. A 10 mm x 2 mm x 0.7 mm (l x w x t) was placed in a tension film clamp. An oscillation amplitude of 15 um and a static force of 0.01 N were employed. A ramp of 3 °C/min was used from – 20 to 175 with a frequency of 1 Hz. T_α was measured as the peak in the Tan delta curve.

S2. Small molecule studies

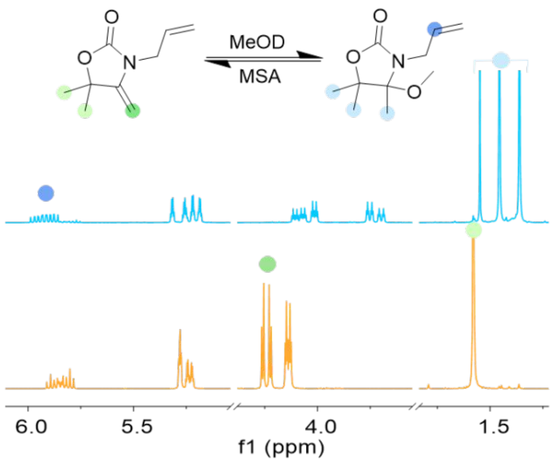

Figure S1. ^{1}H NMR spectra pre and post addition of MSA of Allyl Oxazolidone in MeOD (0.18 M, 1 mol% MSA)

The conversion was calculated with Equation 1 (where I_t is the integral at time t and I_0 the integral at time 0) by taking into consideration the signal of the CH_3 of **AllOx** (1.55 ppm). The signals were normalized using the signal of the allyl bond (multiplet, 5.16 ppm).

$$Conversion\ (\%) = \frac{I_t}{I_0} * (1)$$

S2.1 Hydration of AllOx

AllOx (1 eq) was mixed with water (1 eq). MSA (1 mol%) was added to the reaction and the reaction was stirred at rt. Aliquots were sampled at time 0, 1, 2, 5 and 10 min, quenched with TEA (10 uL) before being dissolved in deuterated DMSO-d_6 (0.5 mL).

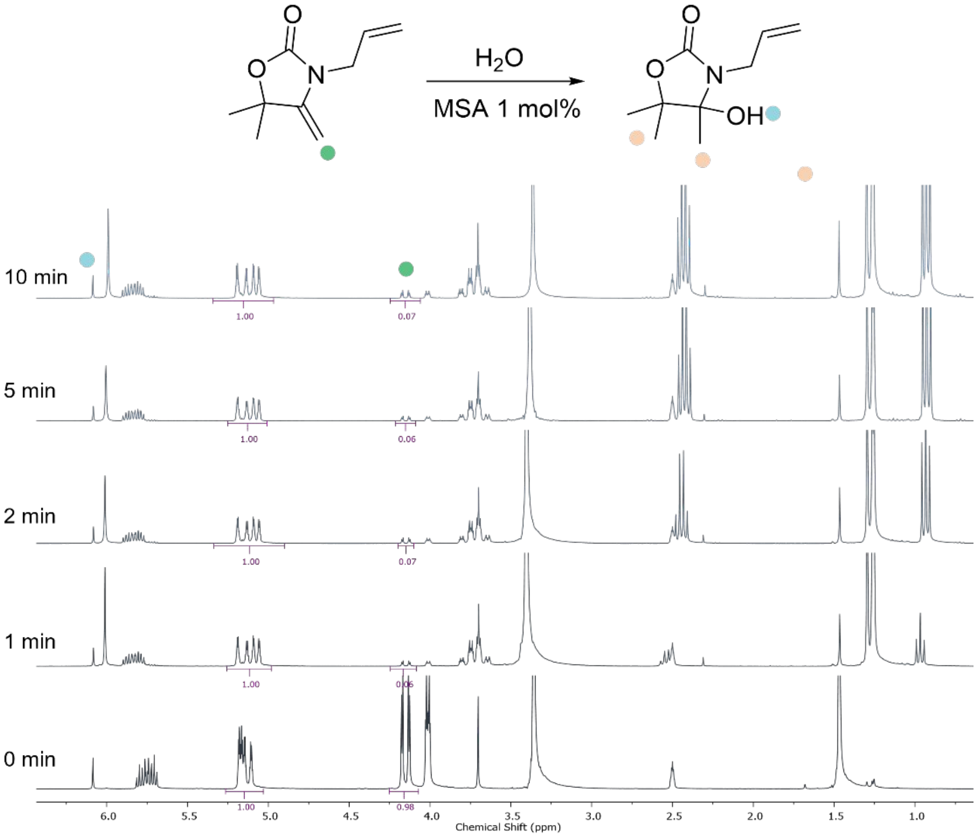

Figure S2. ^{1}H NMR kinetics of hydration of AllOx (conditions: [**AllOx**]/[H_2O] = 1:1, 1 mol% MSA, 25°C)

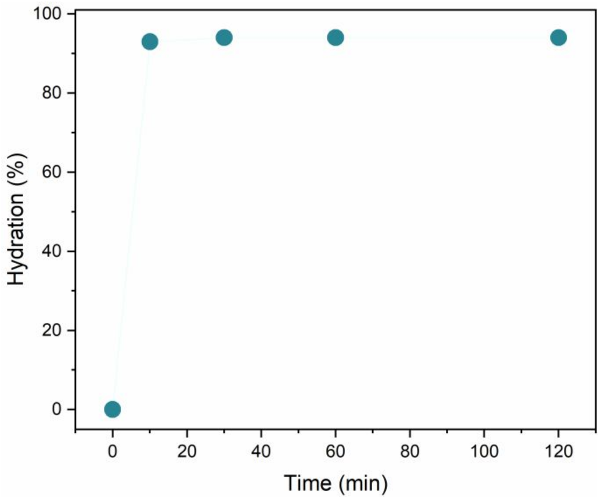

Figure S3. Hydration over time of AllOx (conditions: [**AllOx**]/[H_2O] = 1:1, 1 mol% MSA, 25°C)

S2.2 Alcohol Screening Model Reactions

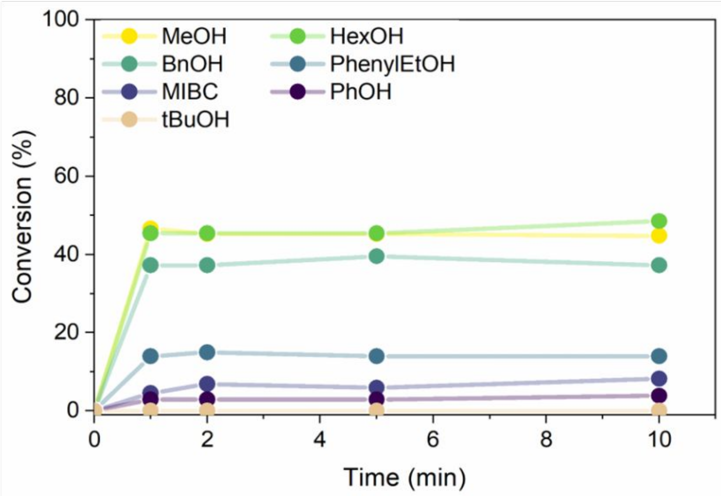

Figure S4. ^{1}H NMR kinetics of the reaction between **AllOx** and a variety of alcoholic partners (MeOH=methanol, HexOH= Hexanol, BnOH=Benzyl Alcohol, MIBC= 4-methyl-2-pentanol, tBuOH= tert-buthanol, PhenylETOH= Phenyl Ethyl Alcohol, PhOH=Phenol) (conditions: [**AllOx**]/[ROH] = 1:1, 1 mol% MSA, 25°C)

Model reactions between **AllOx** and a set of alcohols were carried out. The two components were mixed in an argon-filled glovebox in equal OH to double bond ratio in bulk with the catalyst. Aliquots of the reaction mixture were sampled over time. The reactions were monitored by ^{1}H-NMR spectroscopy to determine the conversion in the product.

For all reaction the conversion was calculated with Equation 1 (where It is the integral at time t and I0 the integral at time 0) by taking into consideration the signal of the CH_2 of exovinylene double bond (3.98 ppm). The signals were normalized using the signal of the allyl bond (multiplet, 5.16 ppm).

Methanol kinetics: **AllOx** (1 eq), anhydrous methanol (1.0 eq) and MSA (1 mol%) were mixed in a glass vial under argon atmosphere. The mixture was sampled at 0, 1, 2, 5 ,10 min and immediately quenched with 10 uL of DBU before being dissolved in DMSO-d_6.

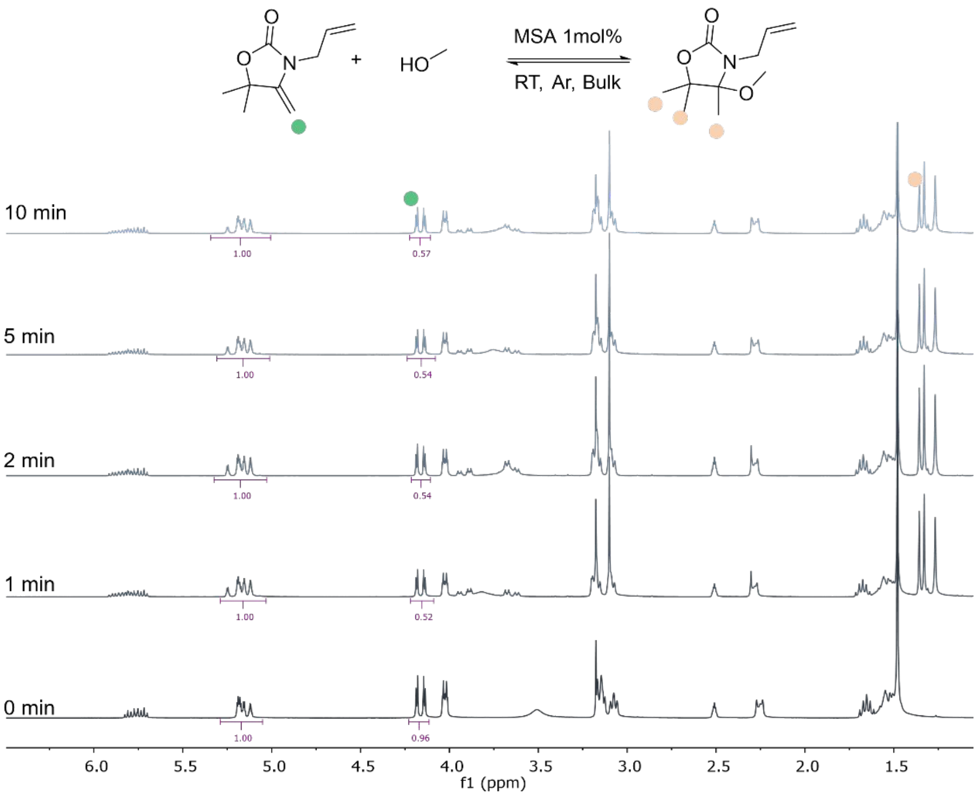

Figure S5. ^{1}H NMR kinetics of the reaction between AllOx and Methanol (conditions: [**AllOx**]/[ROH] = 1:1, 1 mol% MSA, 25°C)

1-Hexanol kinetics: **AllOx** (1 eq), anhydrous 1-hexanol (Distilled over CaH_2, 1.0 eq) and MSA (1 mol%) were mixed in a glass vial under argon atmosphere. The mixture was sampled at 0, 1, 2, 5 ,10 min and immediately quenched with 10 uL of DBU before being dissolved in DMSO-d_6.

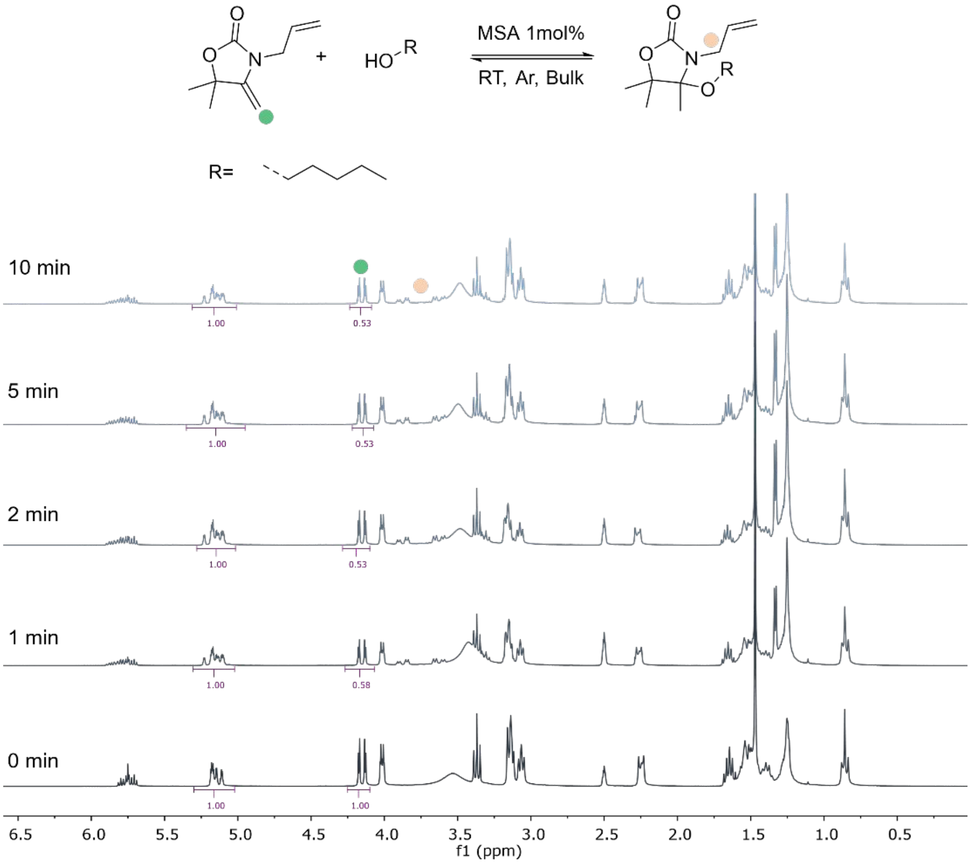

Figure S6. ^{1}H NMR kinetics of the reaction between AllOx and Hexanol (conditions: [**AllOx**]/[ROH] = 1:1, 1 mol% MSA, 25°C)

Benzyl Alcohol kinetics: **AllOx** (1 eq), anhydrous Benzyl Alcohol (Distilled over CaH_2, 1.0 eq) and MSA (1 mol%) were mixed in a glass vial under argon atmosphere. The mixture was sampled at 0, 1, 2, 5 ,10 min and immediately quenched with 10 uL of TEA before being dissolved in DMSO-d_6.

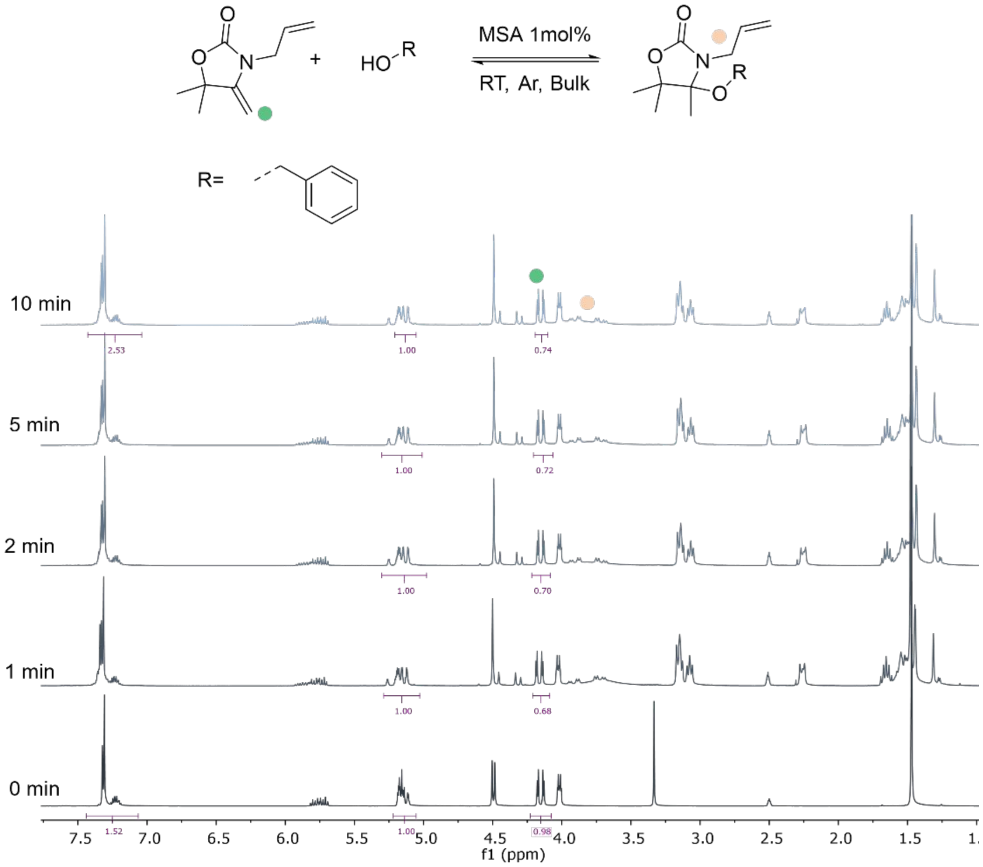

Figure S7. ^{1}H NMR kinetics of the reaction between AllOx and Benzyl alcohol (conditions: [**AllOx**]/[ROH] = 1:1, 1 mol% MSA, 25°C)

Benzy Ethyll Alcohol kinetics: **AllOx** (1 eq), anhydrous Benzyl Ethyl Alcohol (Distilled over CaH_2, 1.0 eq) and MSA (1 mol%) were mixed in a glass vial under argon atmosphere. The mixture was sampled at 0, 1, 2, 5 ,10 min and immediately quenched with 10 uL of TEA before being dissolved in DMSO-d_6.

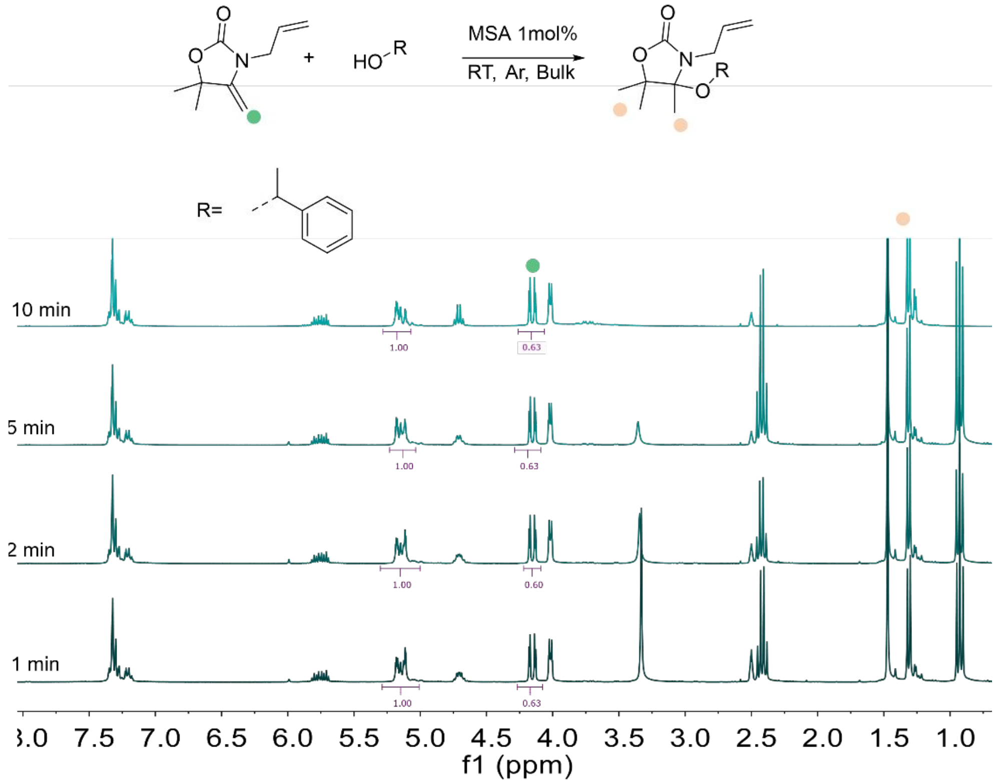

Figure S8. ^{1}H NMR kinetics of the reaction between AllOx and Benzyl Ethyl alcohol (conditions: [**AllOx**]/[ROH] = 1:1, 1 mol% MSA, 25°C)

2-Methyl-4-Pentanol kinetics: **AllOx** (1 eq), anhydrous 2-Methyl-4-Pentanol (Distilled over CaH_2, 1.0 eq) and MSA (1 mol%) were mixed in a glass vial under argon atmosphere. The mixture was sampled at 0, 1, 2, 5 ,10 min and immediately quenched with 10 uL of TEA before being dissolved in DMSO-d_6.

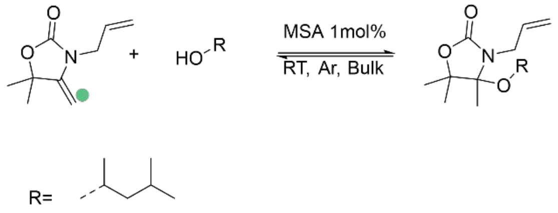

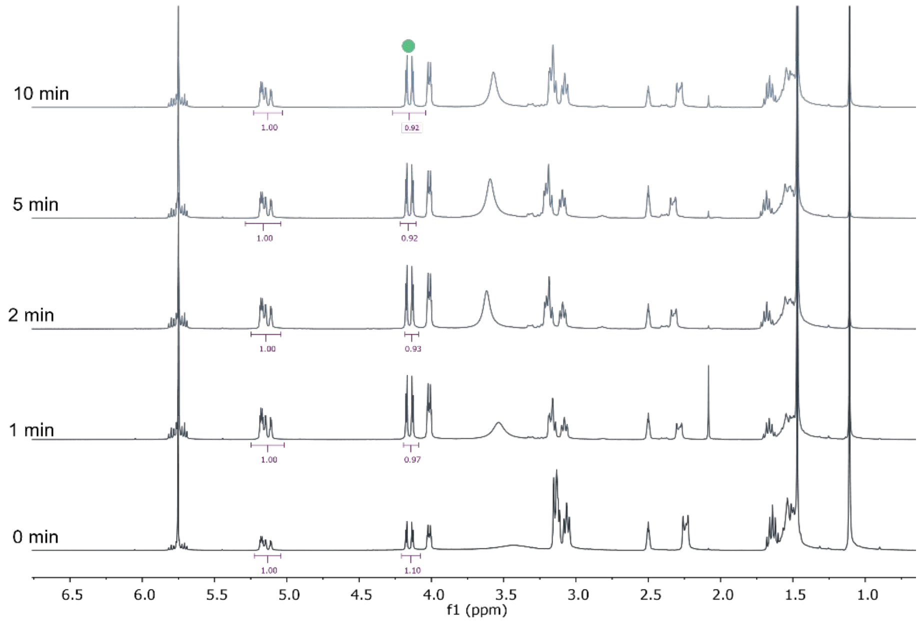

Figure S9. ^{1}H NMR kinetics of the reaction between AllOx and MIBC (conditions: [**AllOx**]/[ROH] = 1:1, 1 mol% MSA, 25°C)

Phenol kinetics: **AllOx** (1 eq), phenol (recrystallised from Toluene, 1.0 eq) and MSA (1 mol%) were mixed in a glass vial under argon atmosphere. The mixture was sampled at 0, 1, 2, 5 ,10 min and immediately quenched with 10 uL of TEA before being dissolved in DMSO-d_6.

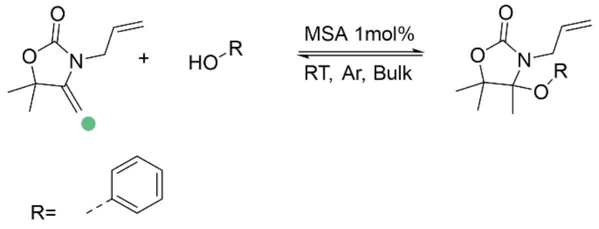

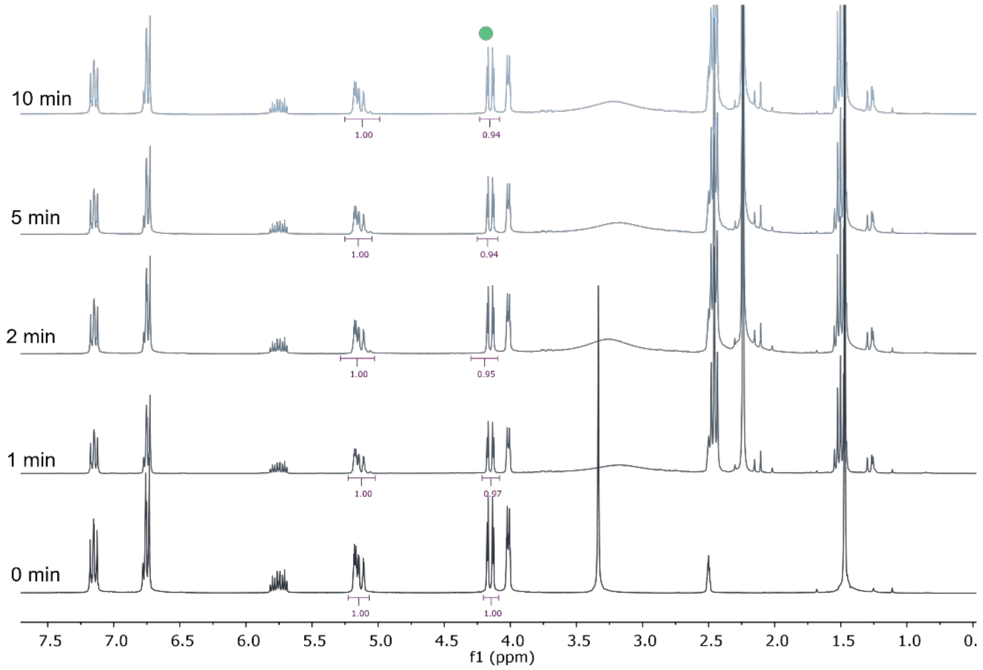

Figure S10. ^{1}H NMR kinetics of the reaction between AllOx and Phenol (conditions: [**AllOx**]/[ROH] = 1:1, 1 mol% MSA, 25°C)

Tert-Butanol kinetics: **AllOx** (1 eq), anhydrous Tert-Butanol (Distilled over CaH_2, 1.0 eq) and MSA (1 mol%) were mixed in a glass vial under argon atmosphere. The mixture was sampled at 0, 1, 2, 5 ,10 min and immediately quenched with 10 uL of TEA before being dissolved in DMSO-d_6.

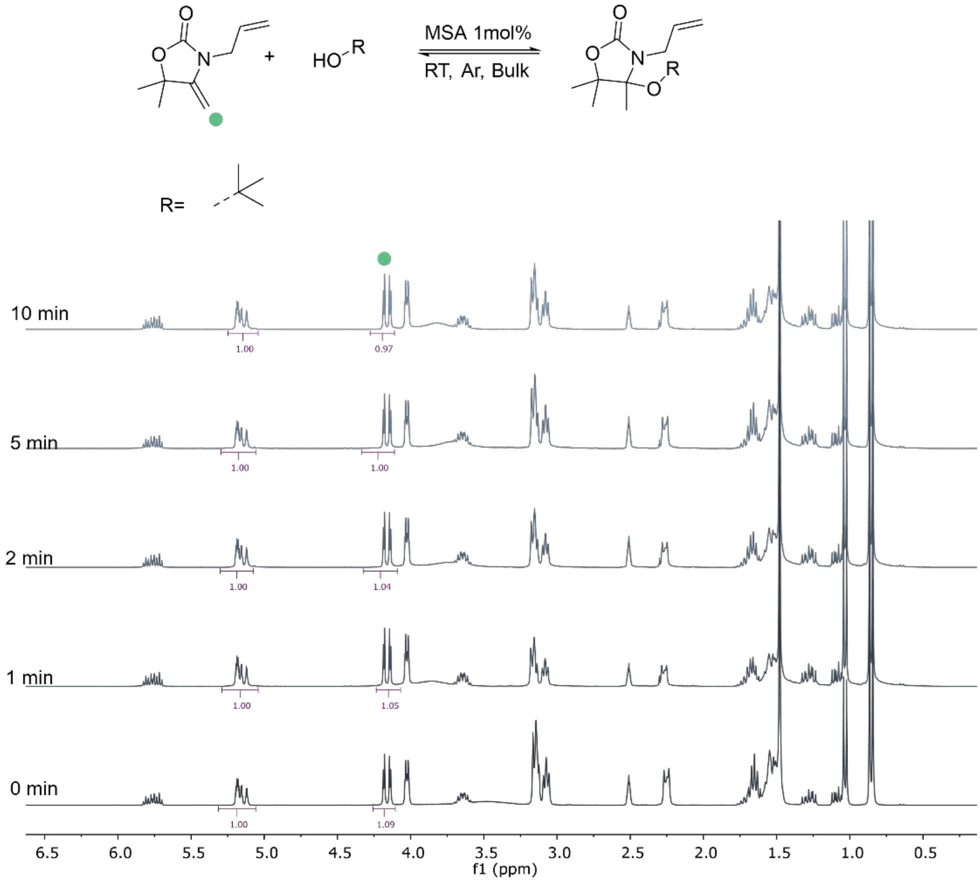

Figure S11. ^{1}H NMR kinetics of the reaction between AllOx and Tert butanol (conditions: [**AllOx**]/[ROH] = 1:1, 1 mol% MSA, 25°C)

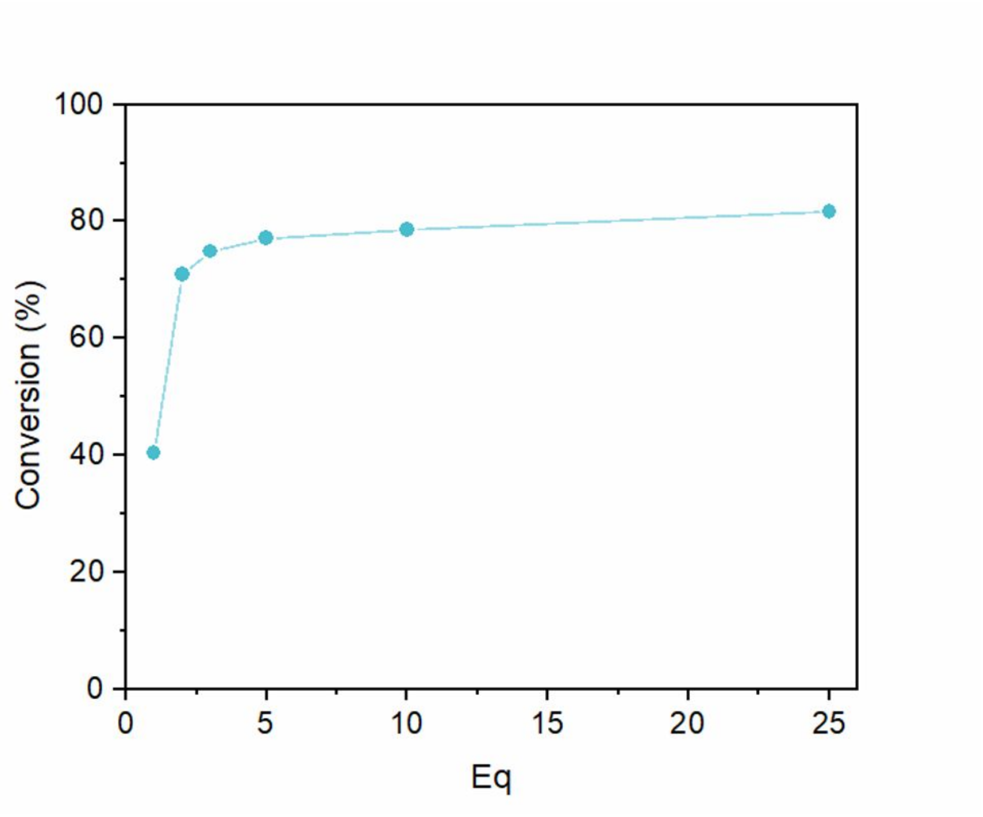

Figure S12. Conversion vs equivalent of methanol for the reaction between AllOx and Methanol (dry). Conditions: Glovebox, 25 °C, 1 mol% MSA, 15 min reaction.

S3. Synthetic Procedures

S3.1 Synthesis of AllOx

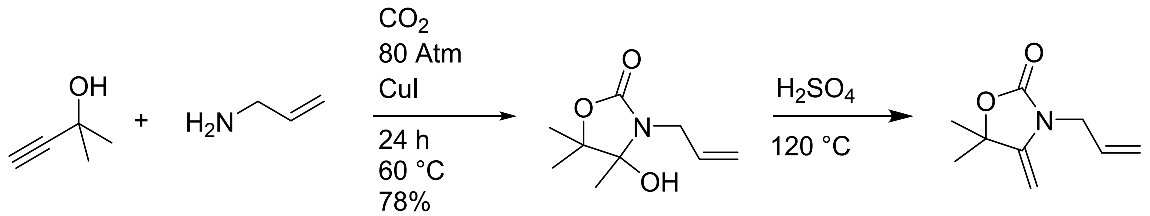

Scheme S1. Synthesis of AllOx

3-allyl-5,5-dimethyl-4-methyleneoxazolidin-2-one (AllOx) was prepared by adapting Jiang *et al.* procedure.[2] 2-Methyl-3-butyn-2-ol (84.1 g, 100 mmol, 1 eq), allyl amine (60 g, 105 mmol, 1.05 eq) and copper iodide (10 g, 5 mol%) were loaded in a stainless-steel autoclave. CO_2 was injected at a pressure of 80 atm and a temperature of 60 °C under mechanical stirring. After 24 h the reaction vessel was depressurized, and the deep red mixture was distilled with a short path distillation apparatus at 120 °C over sulfuric acid (0.5 mL) under vacuum. The

target material was obtained as a colourless oil (130 g, 78% yield) and stored in a freezer (-18 °C).

^{1}H-NMR (300 MHz, DMSO-d_6) δ 5.76 (m, 1H), 5.26 – 4.99 (m, 2H), 4.22 – 4.10 (m, 3H), 4.02 (dt, J = 5.0, 1.7 Hz, 2H), 1.47 (s, 9H). ^{13}C-NMR (75 MHz, DMSO-d_6) δ 154.48, 149.72, 131.17, 116.58, 81.92, 80.31, 42.82, 27.52. IR: 2980, 1760, 1682, 1644. Mp (DSC): 6 °C.

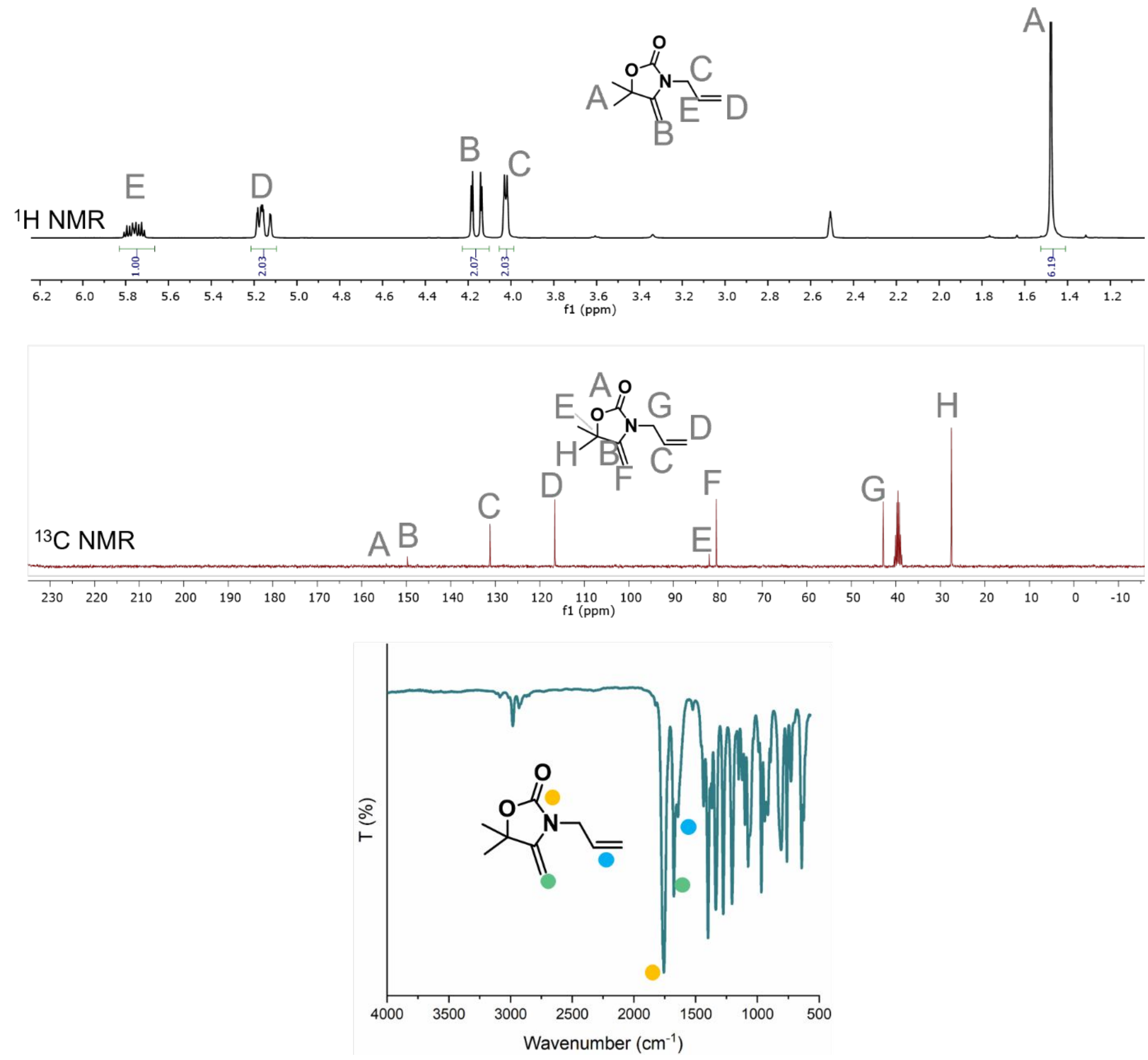

Figure S13. Characterisation of **AllOx**: ^{1}H-NMR, ^{13}C NMR, IR.

S3.2 Synthesis of **1**

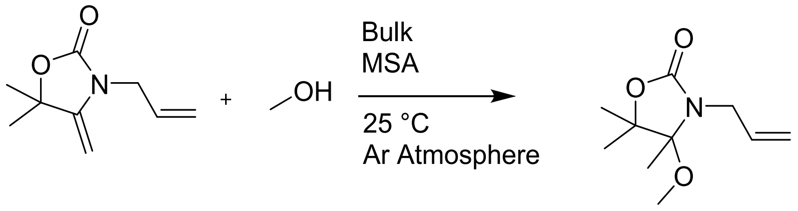

1

Scheme S2. Synthesis of **1**

3-allyl-5,5-dimethyl-4-methyleneoxazolidin-2-one (**AllOx**) (0.5 g, 3 mmol, 1 eq), anhydrous methanol (0.5 g, 15 mmol, 5 eq) and MSA (3 mg, 1 mol%) were mixed in a glass vial under argon atmosphere. The mixture was stirred for 15 min before being quenched with TEA (50 uL). The resulting mixture was diluted with ethyl acetate (25 mL) and washed thrice with water (3x25 mL), once with brine (25 mL). The organic phase was separated and dried over $MgSO_4$ before being evaporated *via* rotary evaporation. The resulting transparent oil was purified by column chromatography (8.5:1.5 Hexane:Ethyl Acetate, I_2 and Vanillin stain, I_2 was used as it selectively stains AllOx but not its derivatives), a colourless oil was obtained (0.36g, 60% yield). The sample was placed in a freezer (-18 °C) for long term storage.

^{1}H NMR (300 MHz, DMSO-d6) δ 5.85 (m, 1H), 5.35 – 4.98 (m, 2H), 3.78 (m, 2H), 3.10 (s, 3H), 1.35 (s, 3H), 1.33 (s, 3H), 1.26 (s, 3H); **^{13}C NMR** (75 MHz, MeOD) δ 187.15, 159.50, 134.81, 131.94, 117.52, 117.35, 95.03, 87.17, 49.85, 49.57, 49.28, 49.00, 48.72, 48.43, 48.15, 44.45, 43.97, 28.12, 26.37, 19.90, 18.93. **HRMS (ESI)** m/z: $[M-H]^+$ calcd for $C_{10}H_{17}NO_3$, 200.183; found, 200.112

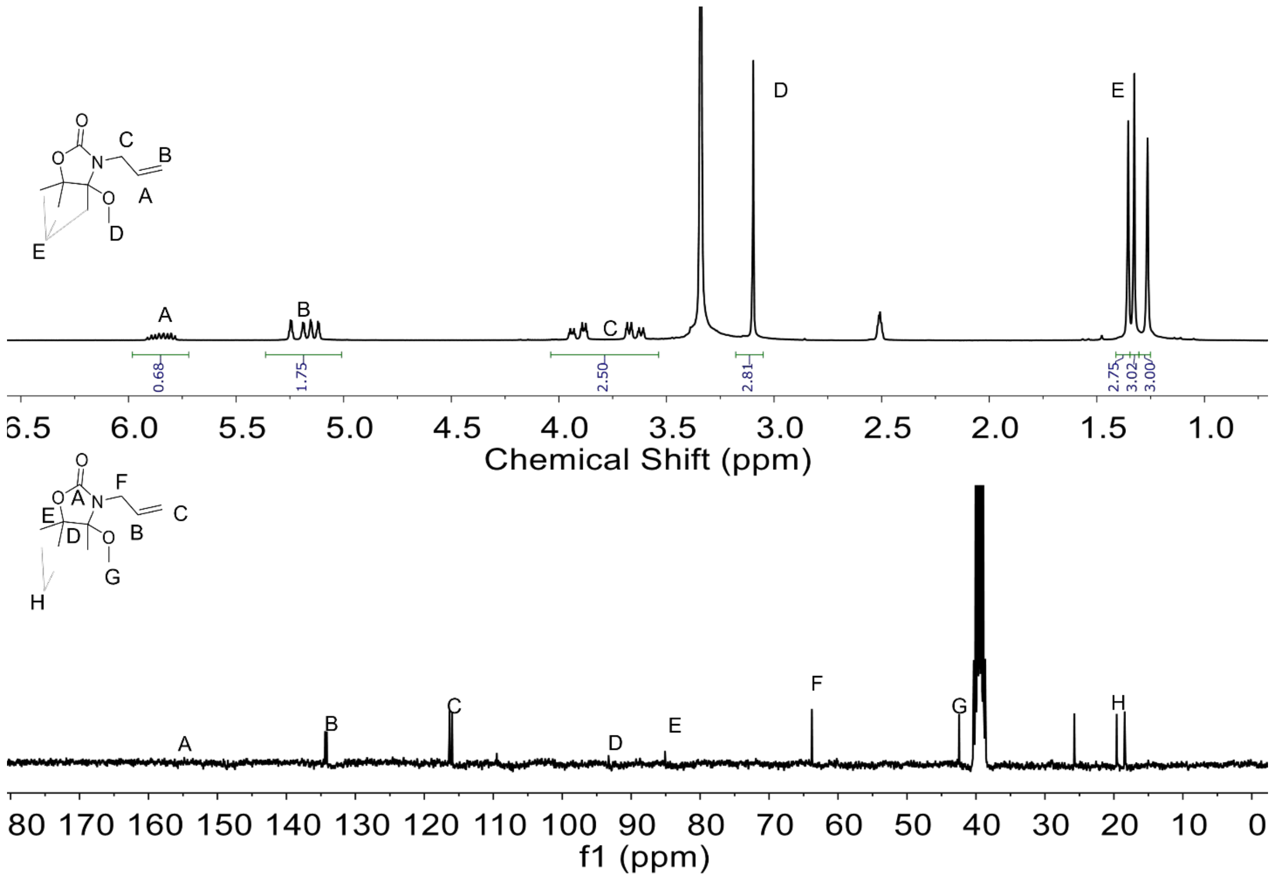

Figure S14. ^{1}H NMR of **1** (top) and ^{13}C NMR (bottom)

S3.3 Synthesis of **2**

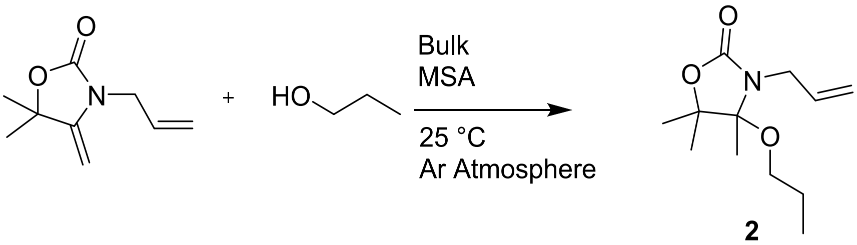

2

Scheme S3. Synthesis of **2**

AllOx (0.5 g, 3 mmol, 1 eq), anhydrous 1-propanol (distilled over CaH_2, 0.190 g, 3.1 mmol, 1.05 eq) and MSA (3 mg, 1 mol%) were mixed in a glass vial under argon atmosphere. The mixture was stirred for 15 min before being quenched with TEA (50 uL). The resulting mixture was diluted with ethyl acetate (25 mL) and washed thrice with water (3x25 mL), once with brine (25 mL). The organic phase was separated and dried over $MgSO_4$ before being evaporated *via* rotary evaporation. The resulting transparent oil was purified by column

chromatography (8.5:1.5 Hexane:Ethyl Acetate, I_2 and Vanillin stain), a slight yellow oil was obtained (0.25g, 35% yield)

^{1}H NMR δ 5.84 (m, 1H), 5.42 – 4.96 (m, 2H), 4.11 – 3.53 (m, 2H), 3.29 – 2.96 (m, 2H), 1.49 (q, *J* = 7.0 Hz, 2H), 1.35 (s, 3H), 1.34 (s, 3H), 1.26 (s, 3H), 0.87 (t, *J* = 7.4 Hz, 3H). **^{13}C NMR** δ 156.30, 134.24, 116.20, 92.89, 84.98, 64.13, 42.33, 25.76, 22.52, 19.46, 18.53, 10.50. **HRMS (ESI)** m/z: $[M\text{-}Na]^+$ calcd for $C_{12}H_{21}NO_3$, 250.289; found, 250.142

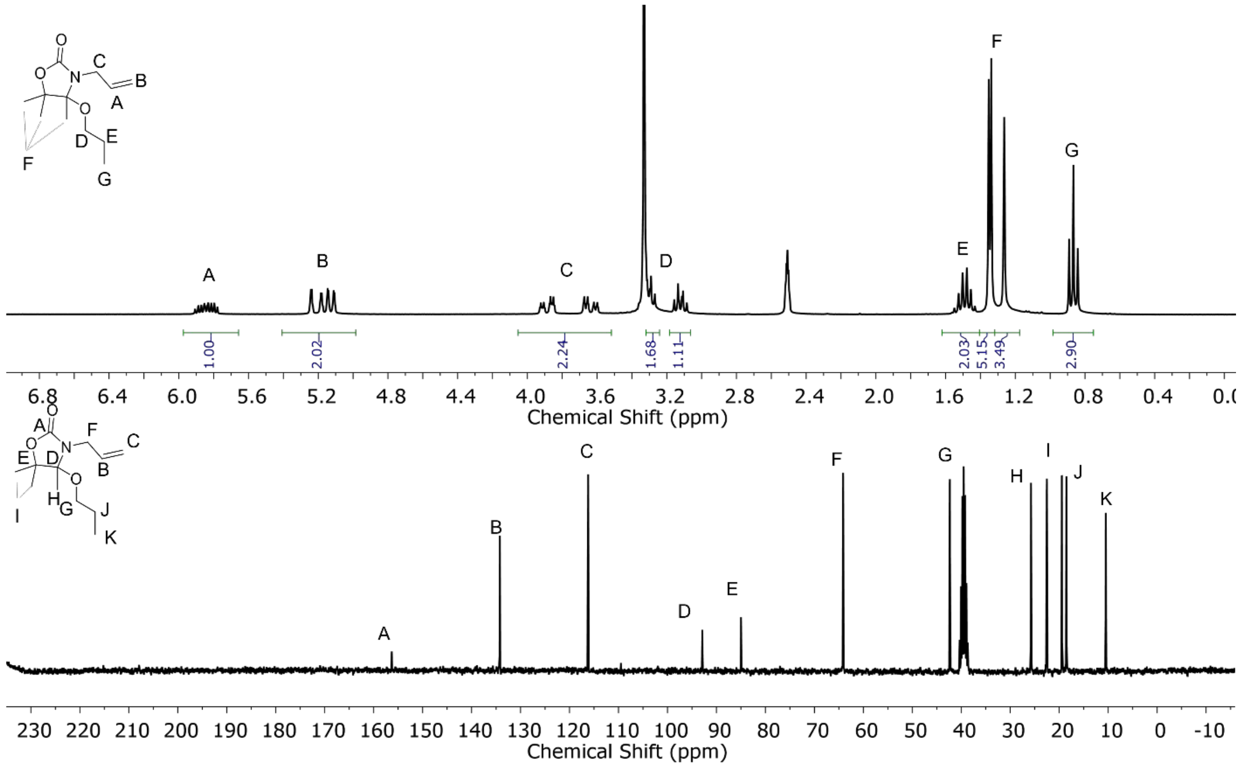

Figure S15. ^{1}H NMR of **2** (top) and ^{13}C NMR (bottom)

S3.4 Synthesis of 3-allyl-4,5,5-trimethyl-4-(prop-2-yn-1-yloxy)oxazolidin-2-one (3)

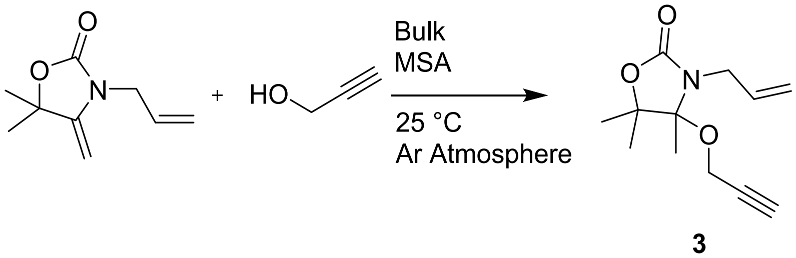

3

Scheme S4. Synthesis of **3**

AllOx (0.5 g, 3 mmol, 1 eq), anhydrous propargylic alcohol (distilled over CaH_2, 0.175 g, 3.1 mmol, 1.05 eq) and **MSA** (3 mg, 1 mol%) were mixed in a glass vial under argon atmosphere. The mixture was stirred for 15 min before being quenched with TEA (50 uL). The resulting mixture was diluted with ethyl acetate (25 mL) and washed thrice with water (3x25 mL), once with brine (25 mL). The organic phase was separated and dried over $MgSO_4$ before being evaporated *via* rotary evaporation. The resulting transparent oil was purified by column chromatography (8.5:1.5 Hexane:Ethyl Acetate, I_2 and Vanillin stain, I_2), a yellow oil was obtained (0.288 g, 43% yield)

^{1}H NMR (300 MHz, DMSO-*d*$_6$) δ 6.07 – 5.68 (m, 1H), 5.39 – 4.96 (m, 2H), 4.09 (qd, J = 15.6, 2.4 Hz, 2H), 3.82 (dddt, J = 59.8, 16.6, 6.0, 1.6 Hz, 2H), 3.45 (t, J = 2.4 Hz, 2H), 1.41 (s, 3H), 1.37 (s, 3H), 1.26 (s, 3H). **^{13}C NMR** (75 MHz, DMSO-*d*$_6$) δ 155.91, 134.07, 116.52, 93.96, 85.17, 80.16, 76.66, 51.66, 42.66, 40.34, 40.06, 39.78, 39.50, 39.22, 38.95, 38.67, 25.48, 19.75, 17.63.**HRMS (ESI)** m/z: $[M-H]^+$ calcd for $C_{12}H_{17}NO_3$, 224.1282; found, 224.1281

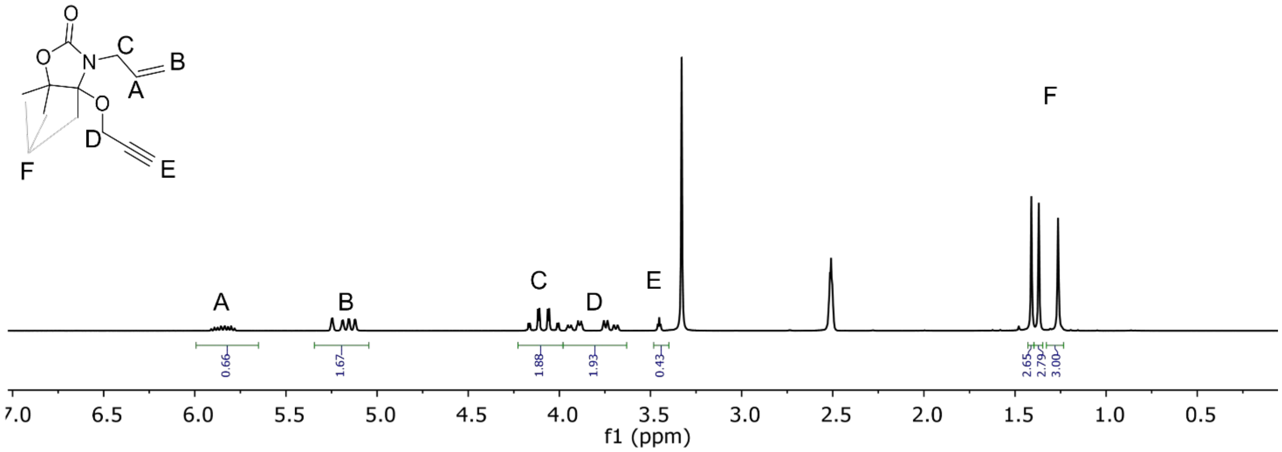

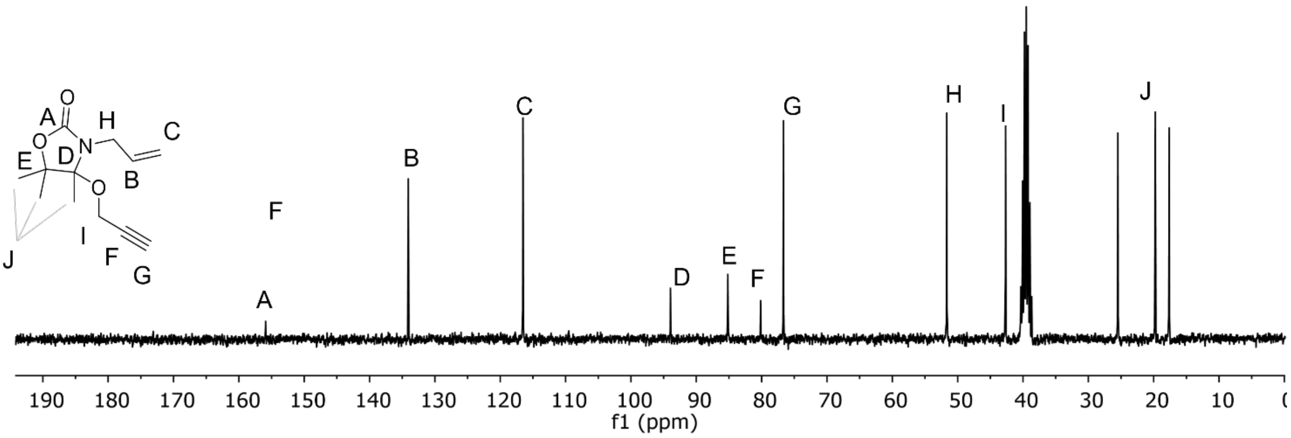

Figure S16. ^{1}H NMR of **3** (top) and ^{13}C NMR (bottom)

S3.5 Synthesis of 3-allyl-4-(allyloxy)-4,5,5-trimethyloxazolidin-2-one (4)

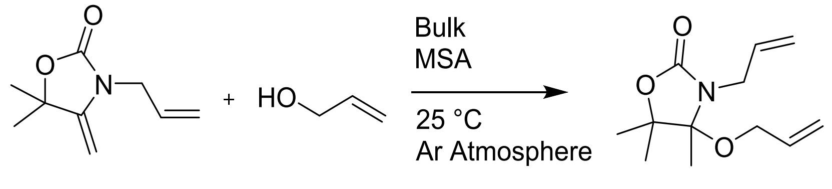

4

Scheme S5. Synthesis of **4**

AllOx (0.5 g, 3 mmol, 1 eq), anhydrous allyl alcohol (distilled over CaH_2, 0.181 g, 3.1 mmol, 1.05 eq) and MSA (3 mg, 1 mol%) were mixed in a glass vial under argon atmosphere. The mixture was stirred for 15 min before being quenched with TEA (50 uL). The resulting mixture was diluted with ethyl acetate (25 mL) and washed thrice with water (3x25 mL), once with brine (25 mL). The organic phase was separated and dried over $MgSO_4$ before being evaporated *via* rotary evaporation. The resulting transparent oil was purified by column chromatography (8.5:1.5 Hexane:Ethyl Acetate, I_2 and Vanillin stain), a colourless oil was obtained (0.324g, 48% yield)

^{1}H NMR (300 MHz, DMSO-d_6) δ 6.10 – 5.66 (m, 2H), 5.40 – 4.89 (m, 4H), 4.02 – 3.58 (m, 4H), 1.38 (s, 3H), 1.36 (s, 3H), 1.28 (s, 3H). **^{13}C NMR** (75 MHz, DMSO-d_6) δ 156.21, 134.42, 134.17, 116.37, 115.99, 93.32, 85.12, 63.83, 42.47, 25.76, 19.63, 18.46. **HRMS (ESI)** m/z: $[M-H]^+$ calcd for $C_{12}H_{19}NO_3$, 226.1440; found, 226.1438

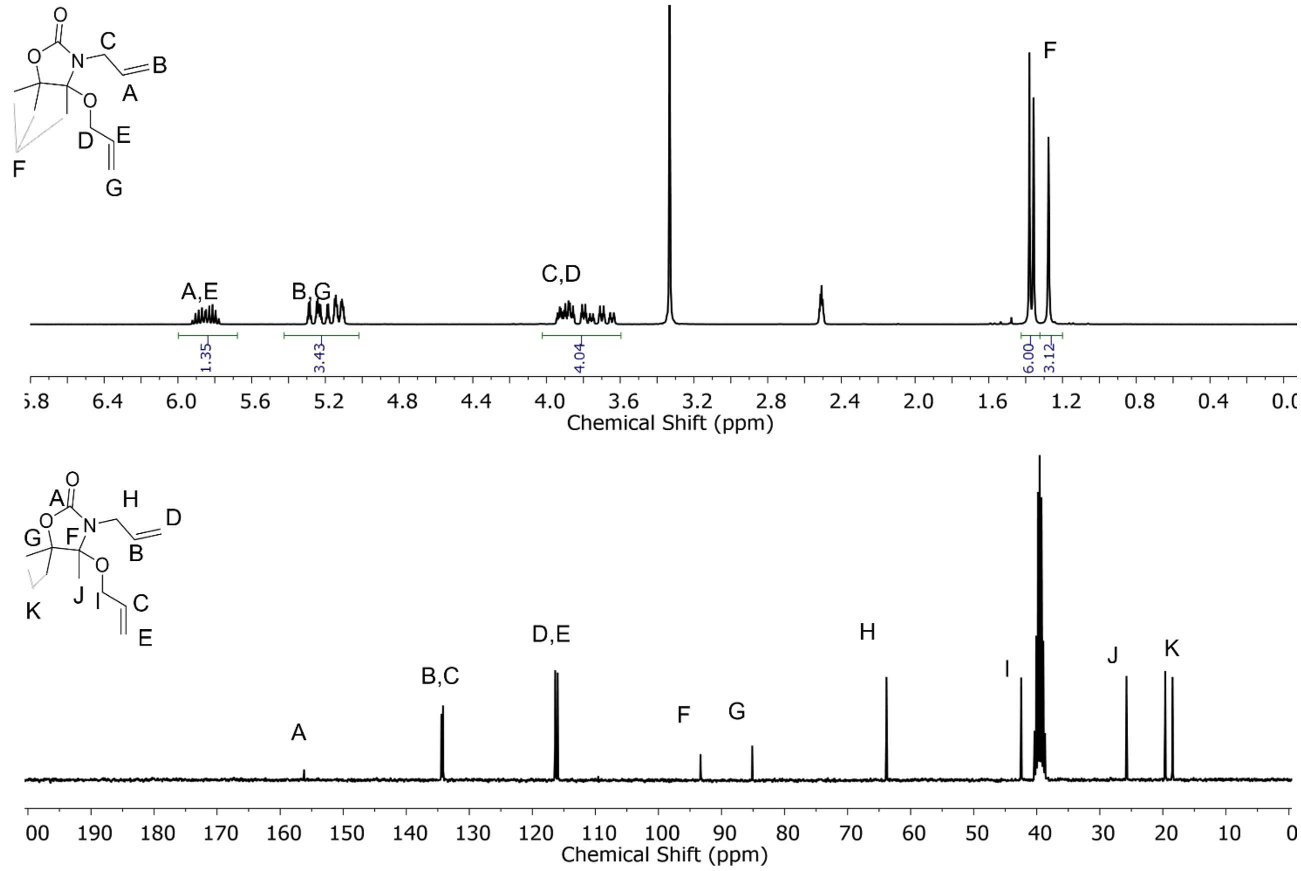

Figure S17. ^{1}H NMR of **4** (top) and ^{13}C NMR (bottom)

S3.6 Synthesis of 3-allyl-4-(benzyloxy)-4,5,5-trimethyloxazolidin-2-one (**5**)

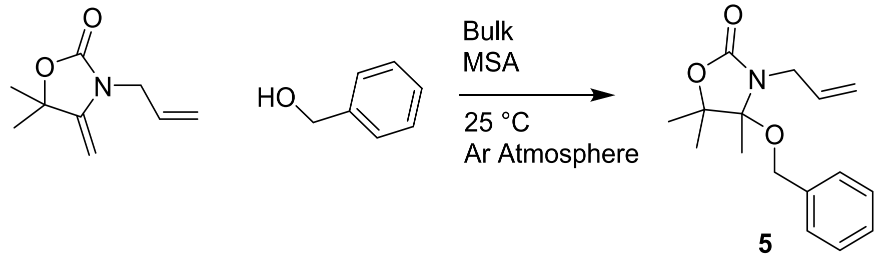

Scheme S6. Synthesis of **5**

3-allyl-5,5-dimethyl-4-methyleneoxazolidin-2-one (AllOx) (0.5 g, 3 mmol, 1 eq), anhydrous benzyl alcohol (distilled over CaH_2, 0.34 g, 3.1 mmol, 1.05 eq) and MSA (3 mg, 1 mol%) were mixed in a glass vial under argon atmosphere. The mixture was stirred for 15 min before being quenched with TEA (50 uL). The resulting mixture was diluted with ethyl acetate (25 mL) and washed thrice with water (3x25 mL), once with brine (25 mL). The organic phase was separated and dried over MgSO4 before being evaporated *via* rotary evaporation. The

resulting transparent oil was purified by column chromatography (8.5:1.5 Hexane:Ethyl Acetate, I_2 and Vanillin stain), a yellow oil was obtained (0.12 g, 15% yield)

^{1}H NMR (300 MHz, DMSO-*d*$_6$) δ 7.51 – 7.13 (m, 5H), 6.03 – 5.70 (m, 1H), 5.42 – 4.97 (m, 2H), 4.61 – 4.21 (m, 2H), 3.96 – 3.63 (m, 2H), 1.44 (s, 6H), 1.31 (s, 3H). **^{13}C NMR** (75 MHz, DMSO-*d*$_6$) δ 156.21, 137.78, 134.16, 128.23, 127.43, 127.26, 116.57, 116.42, 93.53, 85.18, 80.29, 64.74, 42.81, 42.49, 40.34, 40.06, 39.78, 39.50, 39.22, 38.94, 38.66, 27.51, 25.73, 19.68, 18.36. **HRMS (ESI)** m/z: $[M-H]^+$ calcd for $C_{16}H_{22}NO_3$, 276.1597; found, 276.1594

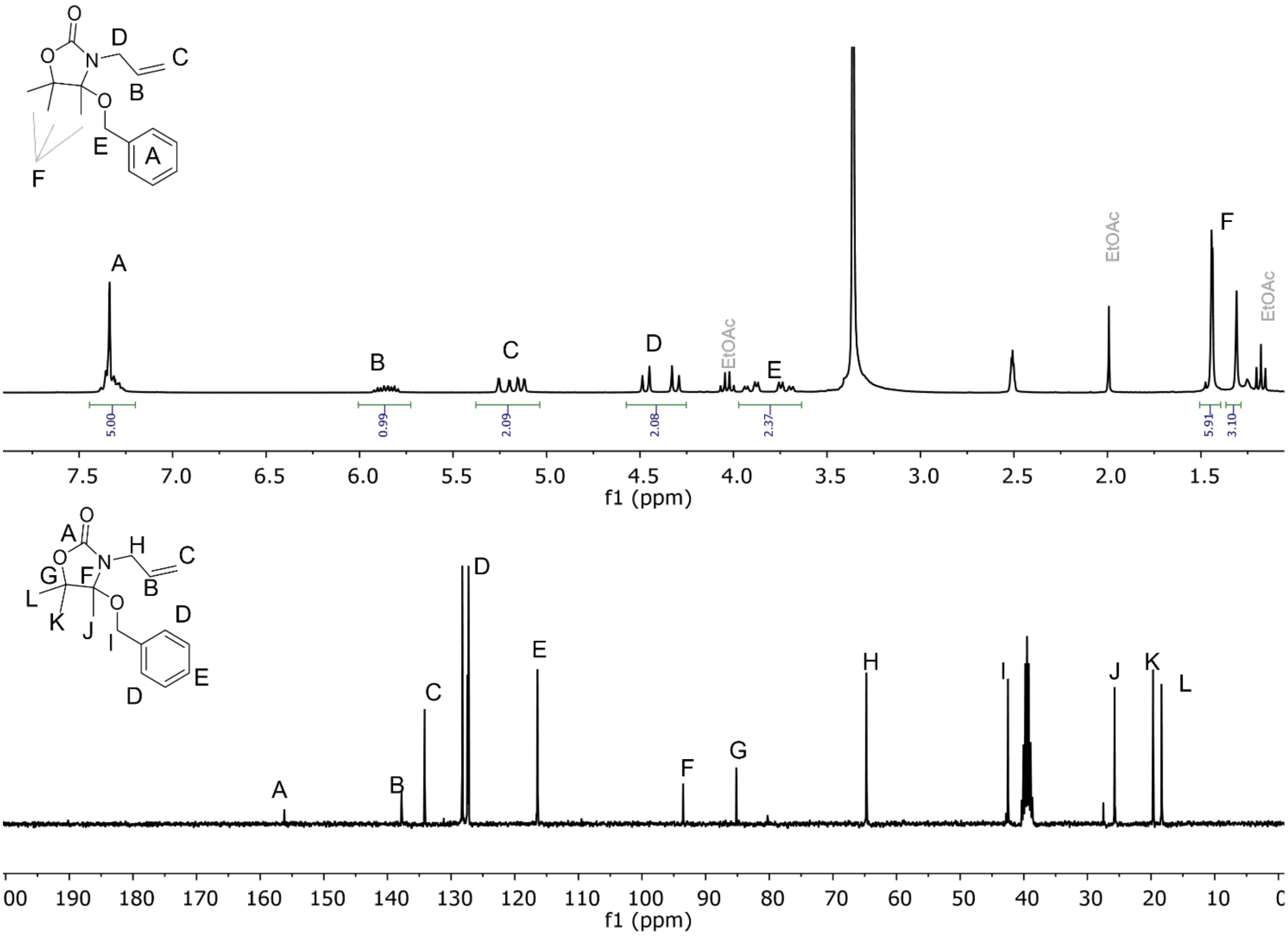

Figure S18. ^{1}H NMR of **5** (top) and ^{13}C NMR (bottom)

S3.7 Synthesis *N*,*S*-acetal model compound (**6**)

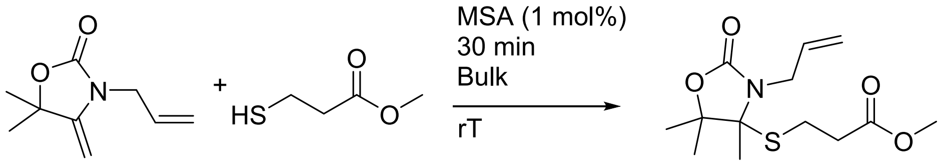

6

Scheme S7. Synthesis of **6**

AllOX (0.6 mmol, 1 eq) was mixed with methyl-3-mercaptopropionate (0.6 mmol, 1 eq) in a glass vial. MSA (1 mol%) was added to the mixture. The homogenous yellow mixture was stirred for 30 min. The resulting transparent mixture was diluted in EtOAc and was washed twice with subsaturated brine. The organic phases were dried over $MgSO_4$ and evaporated using rotary evaporation. The resulting yellow viscous liquid was subjected to column chromatography (Hex:EtOAc 8:2, vanillin and iodine stain). The organic solvent was removed *via* rotary evaporation and the resulting product was obtained as a transparent viscous liquid (yield = 74%).

^{1}H-NMR (300 MHz, DMSO-d_6) δ 6.01 – 5.81 (m, 1H), 5.35 – 5.02 (m, 2H), 3.85 (dddt, 2H), 3.61 (s, 3H), 2.86 – 2.33 (m, 4H), 1.55 (s, 3H), 1.40 (s, 3H), 1.34 (s, 3H).**^{13}C-NMR** (75 MHz, DMSO-d_6) δ 171.50, 155.91, 134.34, 116.50, 85.38, 78.15, 51.55, 42.58, 40.33, 40.06, 39.78, 39.50, 39.22, 38.94, 38.66, 32.58, 24.92, 24.01, 23.01, 20.57 **HRMS (ESI)** m/z: $[M-H]^+$ calcd for $C_{17}H_{30}NO_6S_2H^+$, 288.3740; found, 288.3742

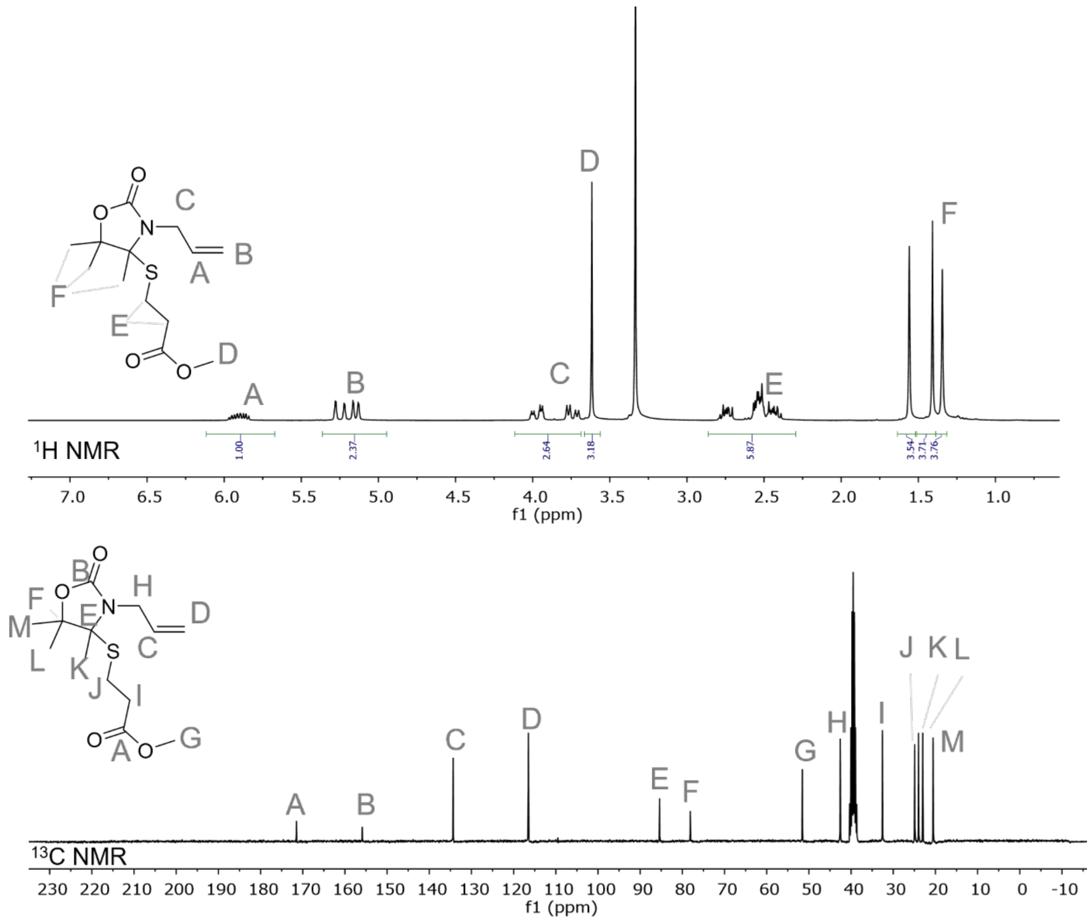

Figure S19. ^{1}H NMR of **6** (top) and ^{13}C NMR (bottom)

S4. Temperature Screening Model Reactions

Model reactions between AllOx and hexanol at different temperatures were carried out. The two components were mixed in an argon-filled glovebox in equal OH to double bond ratio in bulk with the catalyst. Aliquots of the reaction mixture were sampled over time and quenched with a base. The reactions were monitored by ^{1}H-NMR spectroscopy to determine the conversion in the product. For all reaction the conversion was calculated with Equation 1 by taking into consideration the signal of the CH_2 of exovinylene double bond (3.98 ppm). The signals were normalized using the signal of the allyl bond (multiplet, 5.16 ppm). A Van't Hoff plot was built as previously reported.[3]

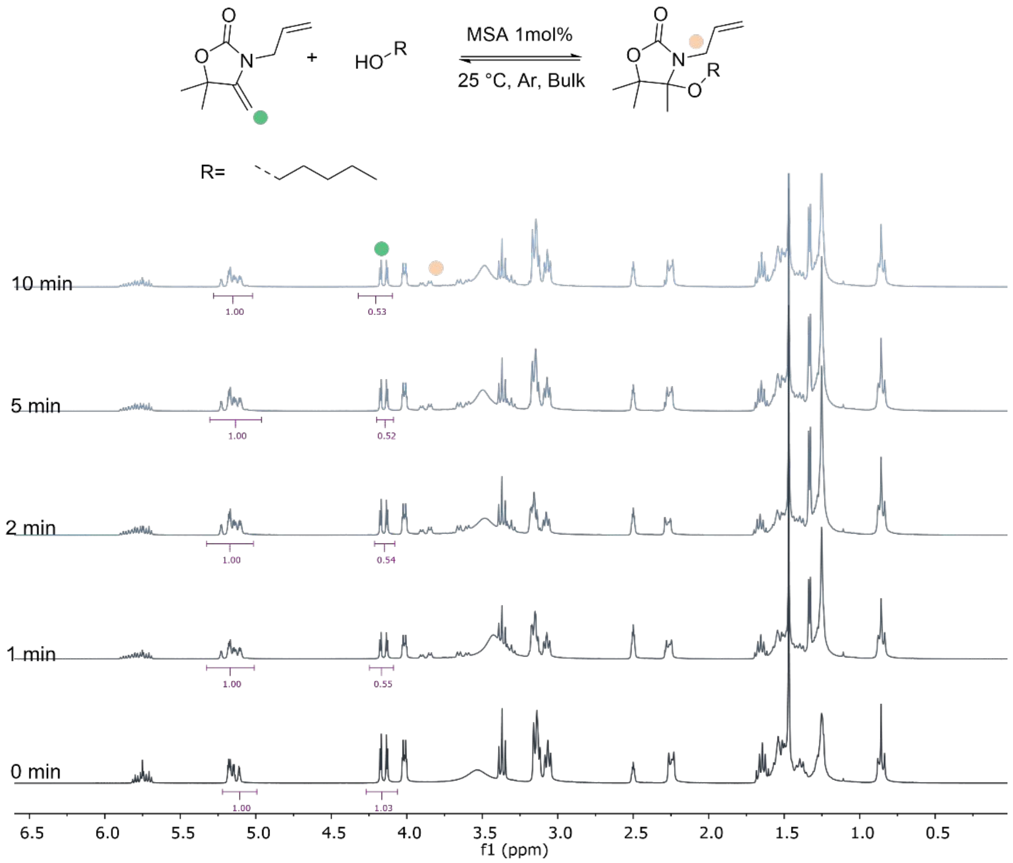

Figure S20. ¹H NMR kinetics of the reaction between AllOx and Hexanol at 25 °C. (conditions: [**AllOx**]/[HexOH] = 1:1, 1 mol% MSA, 25°C)

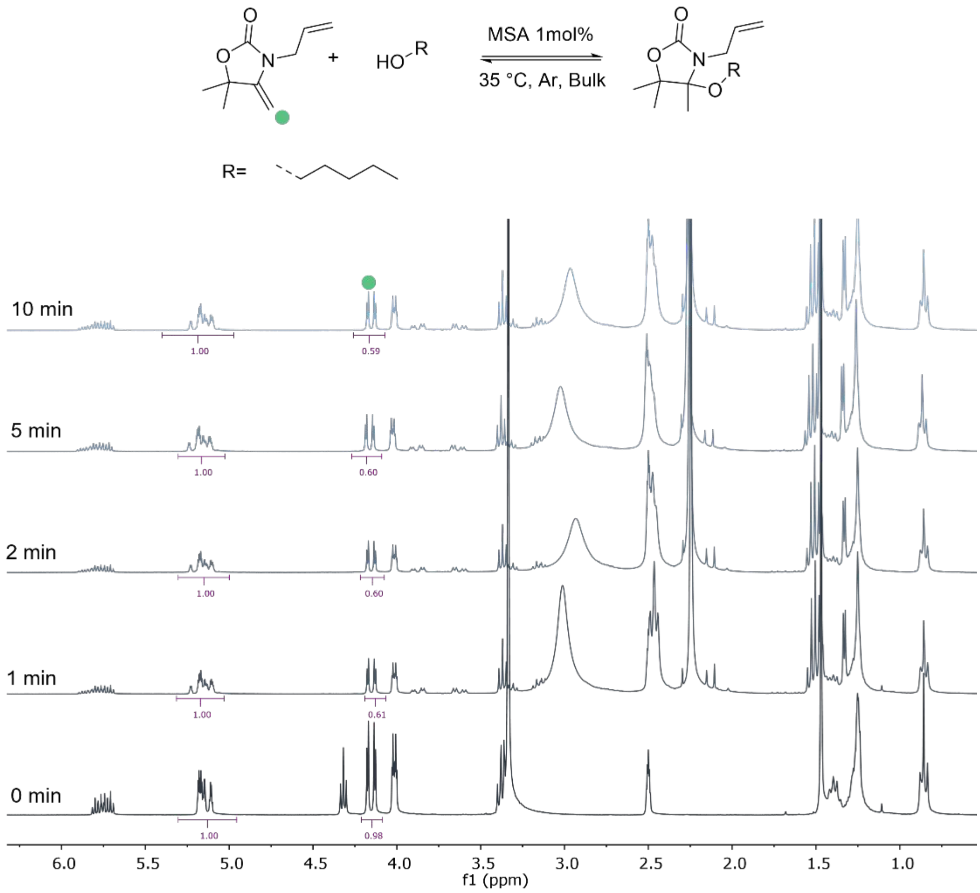

Figure S21. ^{1}H NMR kinetics of the reaction between AllOx and Hexanol at 35 °C (conditions: [**AllOx**]/[HexOH] = 1:1, 1 mol% MSA, 35°C)

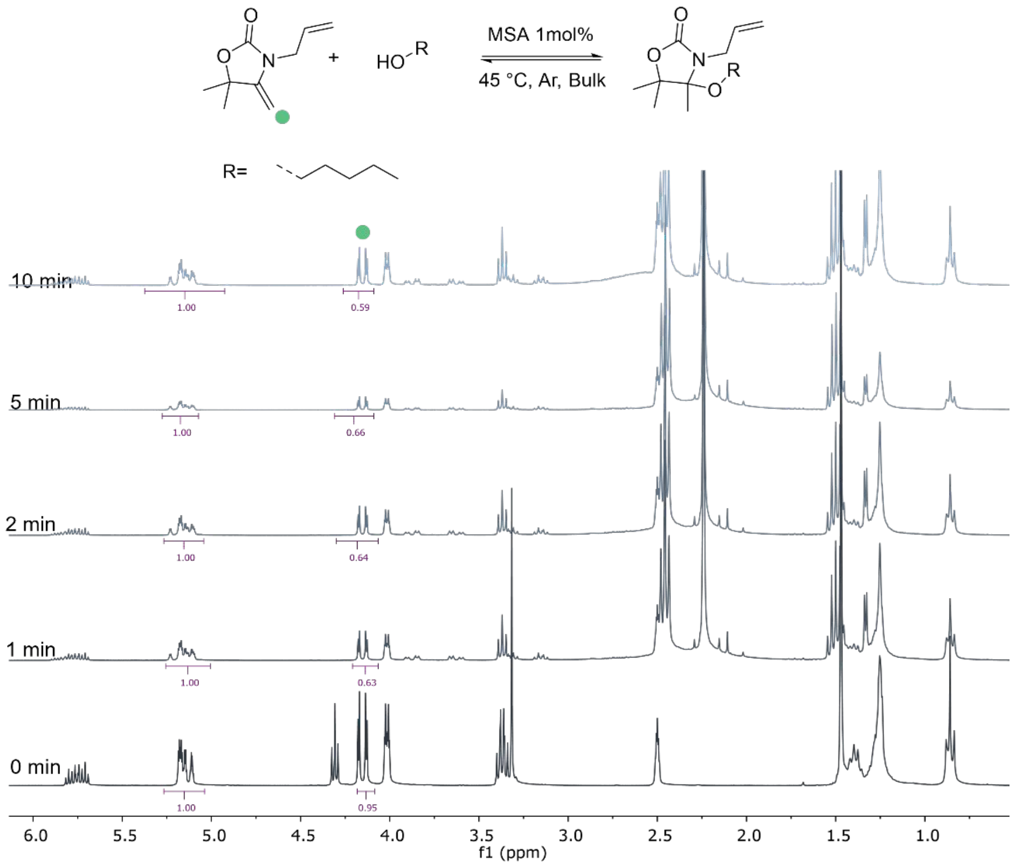

Figure S22. ^{1}H NMR kinetics of the reaction between AllOx and Hexanol at 45 °C. (conditions: [**AllOx**]/[HexOH] = 1:1, 1 mol% MSA, 45°C)

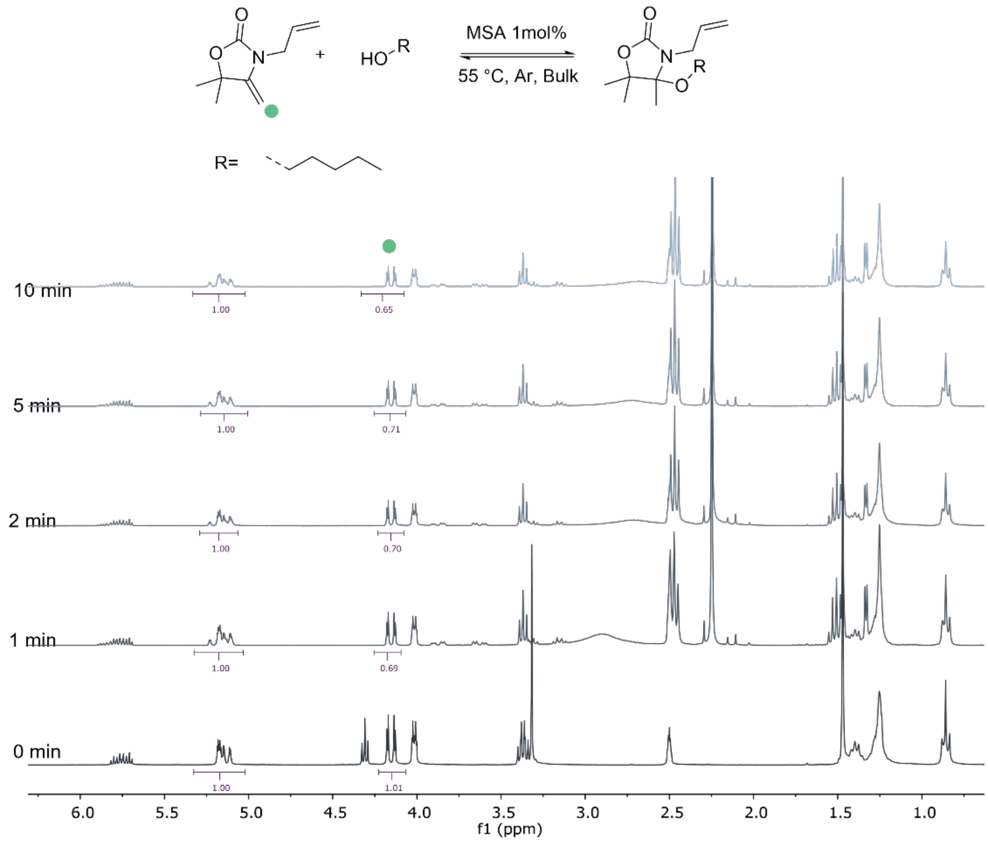

Figure S23. ^{1}H NMR kinetics of the reaction between AllOx and Hexanol at 55 °C. (conditions: [**AllOx**]/[HexOH] = 1:1, 1 mol% MSA, 55°C)

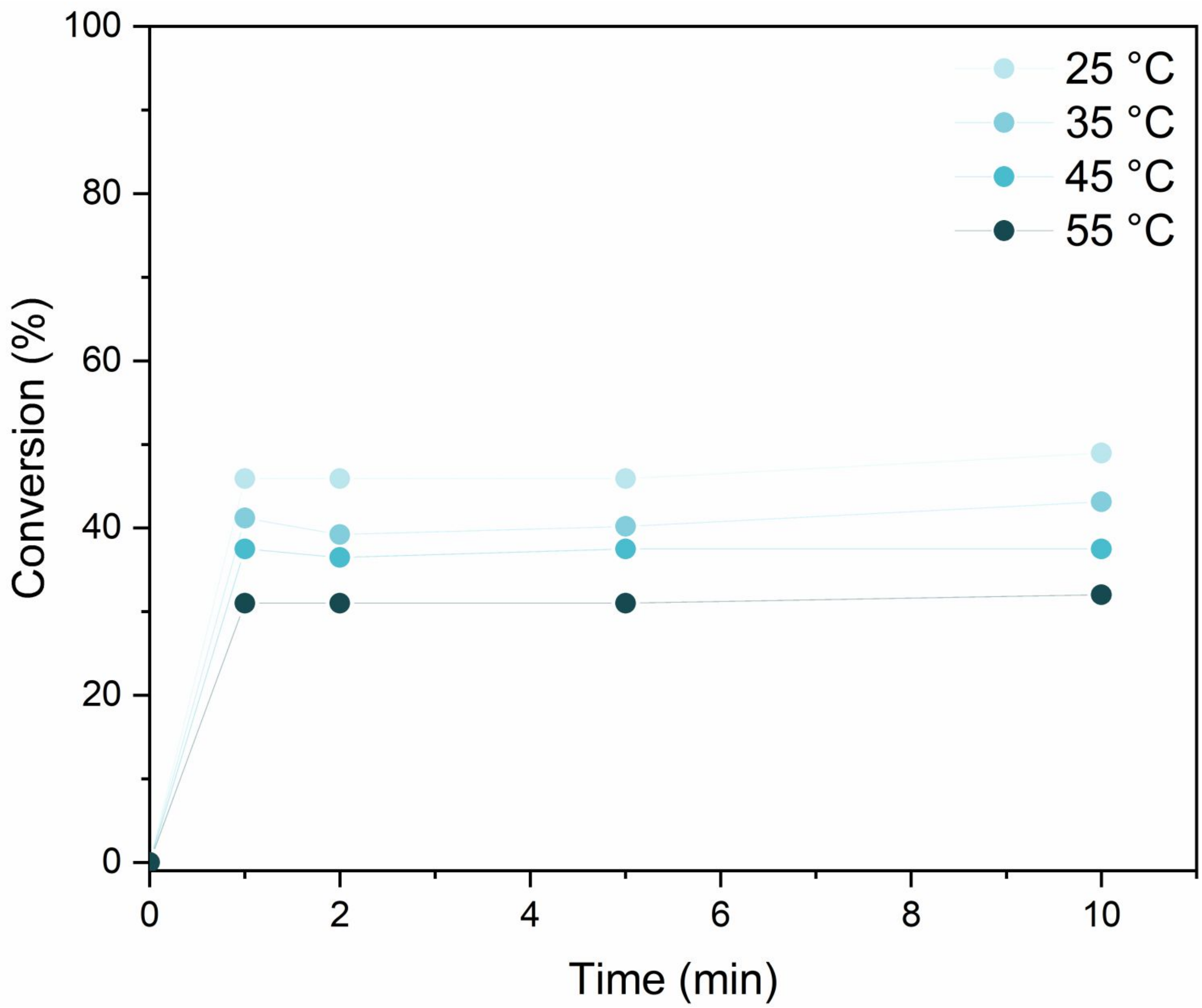

Figure S24. Kinetics of the reaction between Hexanol and **AllOx** at temperature ranging from 25 to 55 °C

S5. DFT

All geometry optimizations were executed within density functional theory (DFT) using the ωB97XD[4] functional combined with the 6-31+G(d)[5] basis set for all atoms. Frequency calculations were carried out at the same level of theory to validate that the optimized structures were minima or transition states on the potential energy surfaces. These frequencies were then used to evaluate the zero-point vibrational energy (ZPVE) and the thermal corrections to the enthalpy (H) and Gibbs free energy (G), at T = 298.15 K, in the

harmonic oscillator approximation. To refine the electronic energies, single-point calculations using the 6-311++G(2df,2p) basis set[6] were performed on the optimized structures. All the calculations were performed with the Gaussian 16 suite of programs.[7] The molecular models used in the simulations compounds are depicted below.

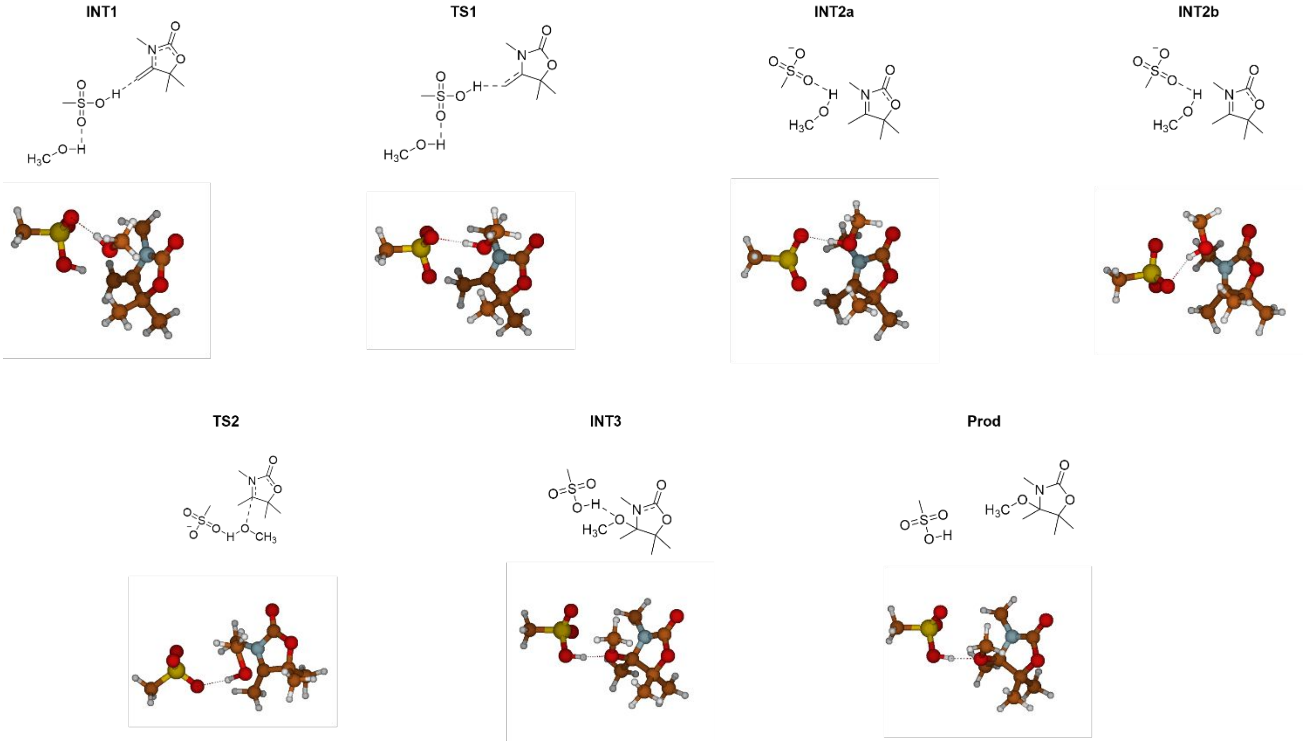

Figure S25. 2D and 3D structures of optimised intermediate and transition states of the modelled reaction path

Table S1 Bond Length evolution along the reaction path for intermediates and transition states.

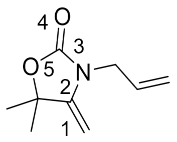

	SR	Int1	TS1	Int2a	Int2b	TS2	Int3	SP
C-C (1)	1.335	1.346	1.403	1.467	1.464	1.497	1.519	1.519
C-N (2)	1.384	1.368	1.320	1.293	1.292	1.318	1.431	1.431
N-C (3)	1.382	1.389	1.422	1.457	1.456	1.418	1.357	1.375
C-O (4)	1.205	1.203	1.195	1.189	1.189	1.192	1.206	1.207
C-O (5)	1.349	1.345	1.333	1.326	1.327	1.334	1.358	1.358

The markedly higher reactivity of thiols compared to alcohols with the exovinylene double bond under cationic conditions can be rationalized by analysing the HOMO-LUMO interaction that occur during the reaction. Since the cationic thiol–ene reaction proceeds through a carbocation at the α-position of nitrogen, the nucleophilic attack of this intermediate depends on the efficiency of HOMO (nucleophile)–LUMO (electrophile) interaction. Thiols (R–SH) possess a higher-energy HOMO than alcohols (R–OH), due to the lower electronegativity and greater polarizability of sulphur relative to oxygen. This results in more favourable orbital overlap with the LUMO of the carbocation intermediate, facilitating a more efficient reaction. In contrast, alcohols have more tightly bound, lower-energy HOMOs, leading to less effective interaction with the carbocationic LUMO and, consequently, lower reactivity.

This effect can also be approached using hard and soft acids and bases principle as the electrophilic centre in the cationic intermediate is relatively soft—being a stabilized, polarizable carbocation. Thiols, as soft nucleophiles, are well matched for soft electrophiles, leading to strong and favourable interactions. In contrast, alcohols are hard nucleophiles (due to the small, highly electronegative oxygen atom), resulting in a less favourable hard–soft mismatch. This mismatch reduces the driving force for nucleophilic attack, further explaining the reduced conversion observed with alcohols.

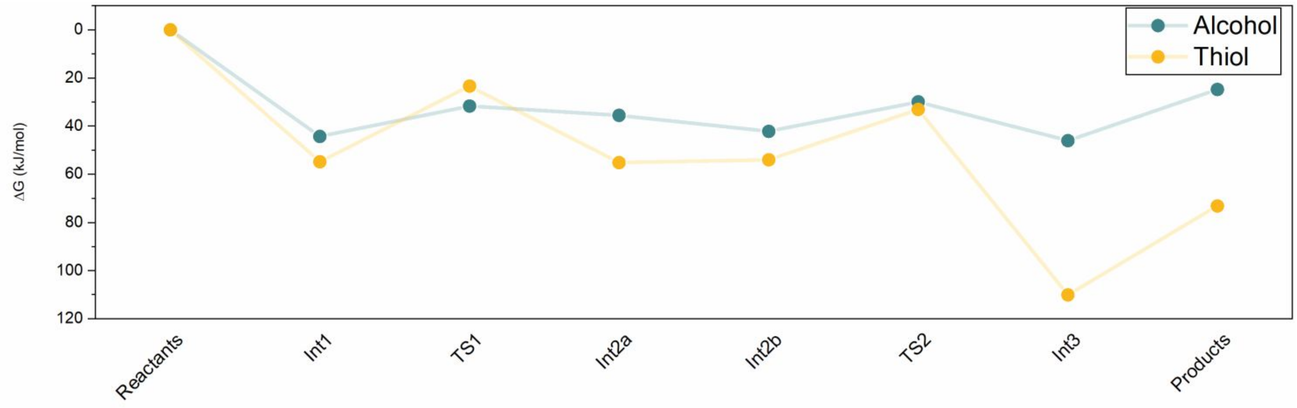

Figure S26. Gibbs-free energy profile of the reaction pathway for the formation of the N,O-acetal moiety compared to the N,S-acetal moiety (Data from ref 8)[8].

S6. In situ NMR of dissociation of D with temperature

4 was placed in a vial under argon atmosphere and its ^{1}H NMR spectrum was measured at temperature interval of 20 °C from 20 to 100 °C. It was then dissolved in dry DMSO-d_6. The sample was given 15 min to equilibrate at each temperature before measuring its ^{1}H NMR spectrum. For the sample containing 1 mol% MSA, the ^{1}H NMR spectra was measured in situ by heating the same tube at different temperatures. A stock solution MSA was previously prepared and added directly to an NMR tube containing **4** and dry DMSO-d_6 (0.18 M).

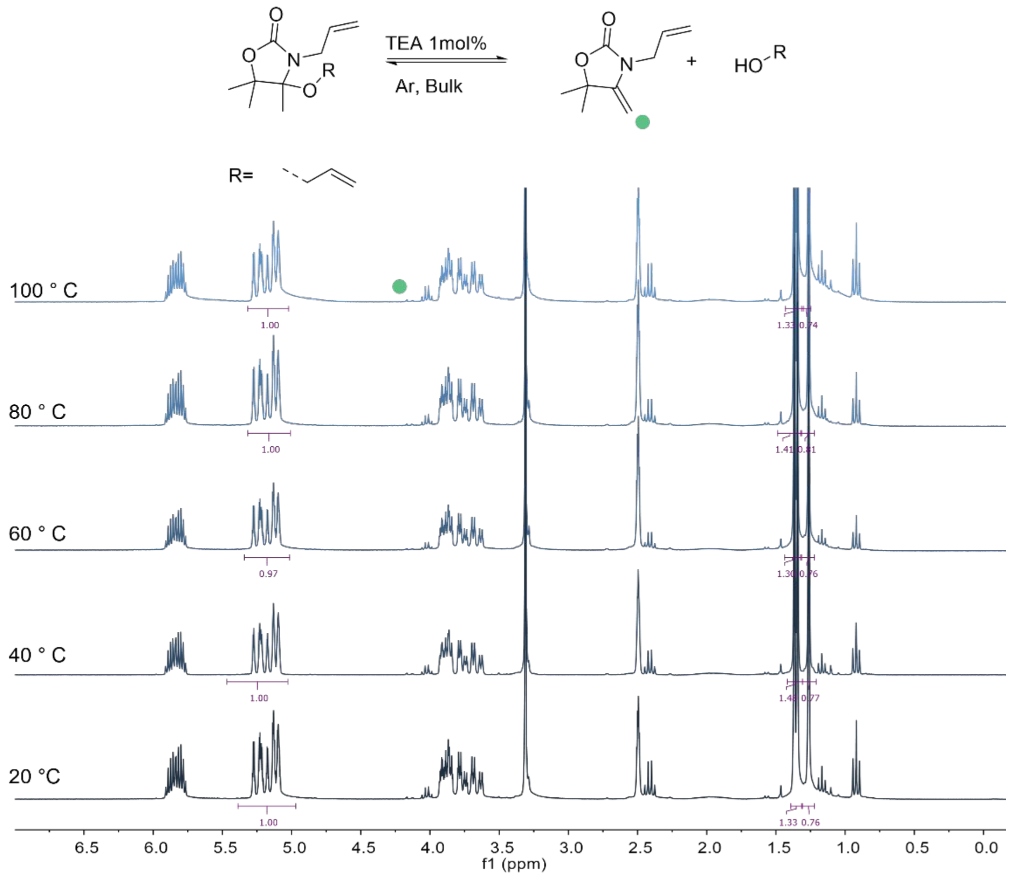

Figure S27. ^{1}H NMR spectra of **4** at different temperatures in dry DMSO-d_6 with 1 mol% TEA

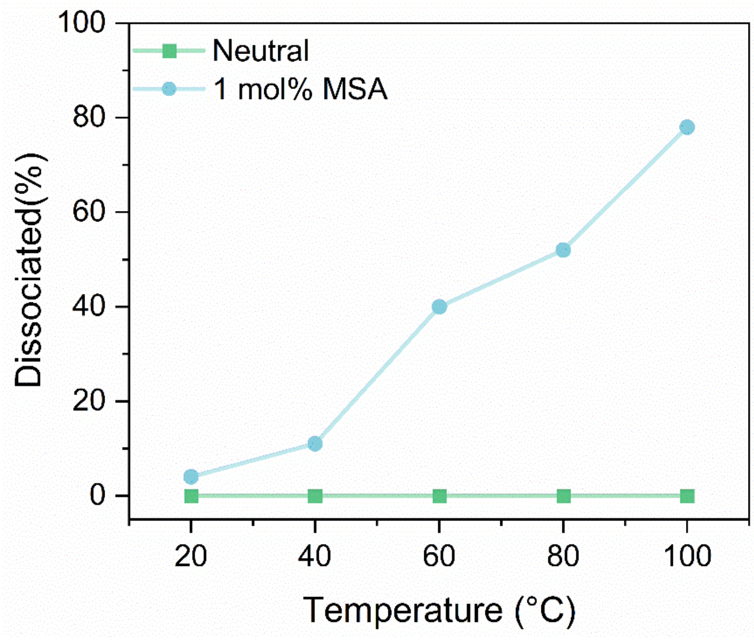

Figure S28. Dissociation of **4** at different temperatures in dry DMSO-d_6

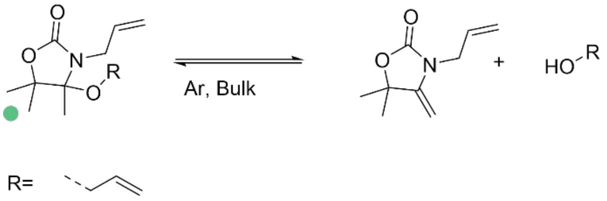

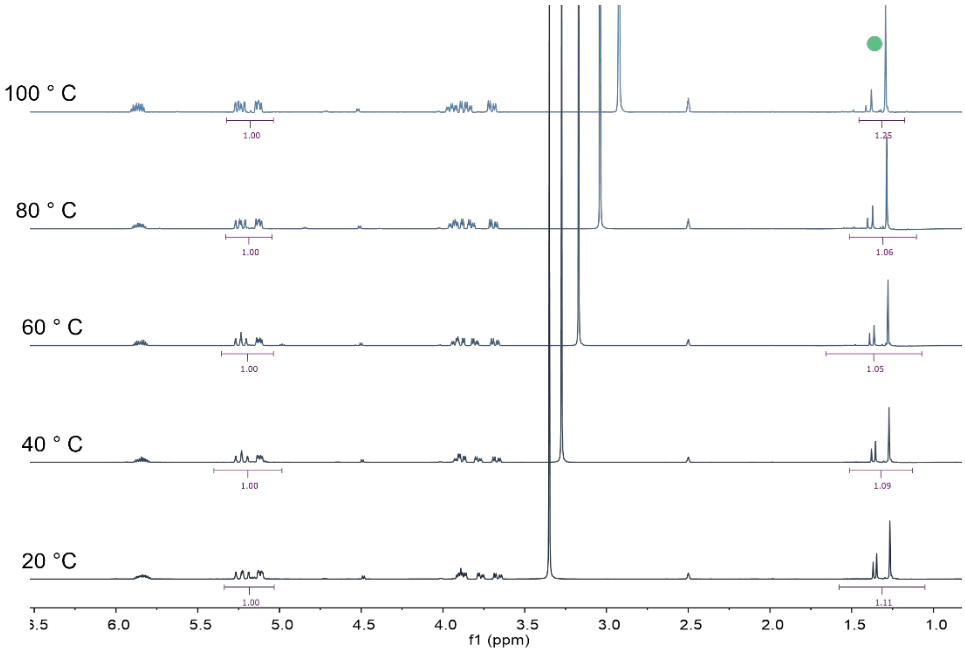

Figure S29. ^1H NMR spectra of **4** at different temperatures in dry DMSO-d_6

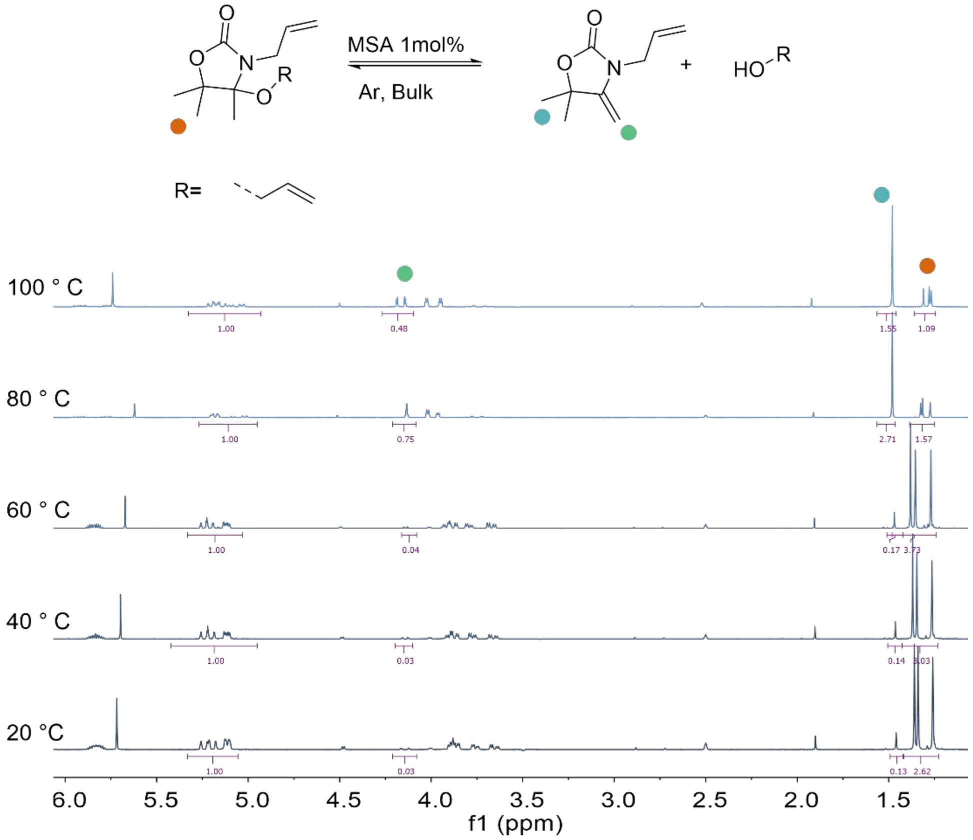

Figure S30. ^{1}H NMR spectra of **4** at different temperatures in dry DMSO-d_6 with 1 mol% MSA

S7. Catalyst Screening

Model reactions between **AllOx** and hexanol with different amounts of catalyst were carried out. MSA, TFA were screened at 1 mol% and 0.1 mol% concentration. The two components were mixed in an argon-filled glovebox in equal ROH to double bond ratio in bulk with the catalyst. Aliquots of the reaction mixture were sampled over time. The reactions were monitored by ^{1}H-NMR spectroscopy to determine the conversion in the product.

For all reaction the conversion was calculated with Equation 1 by taking into consideration the signal of the CH_2 of exovinylene double bond (3.98 ppm). The signals were normalized using the signal of the allyl bond (multiplet, 5.16 ppm).

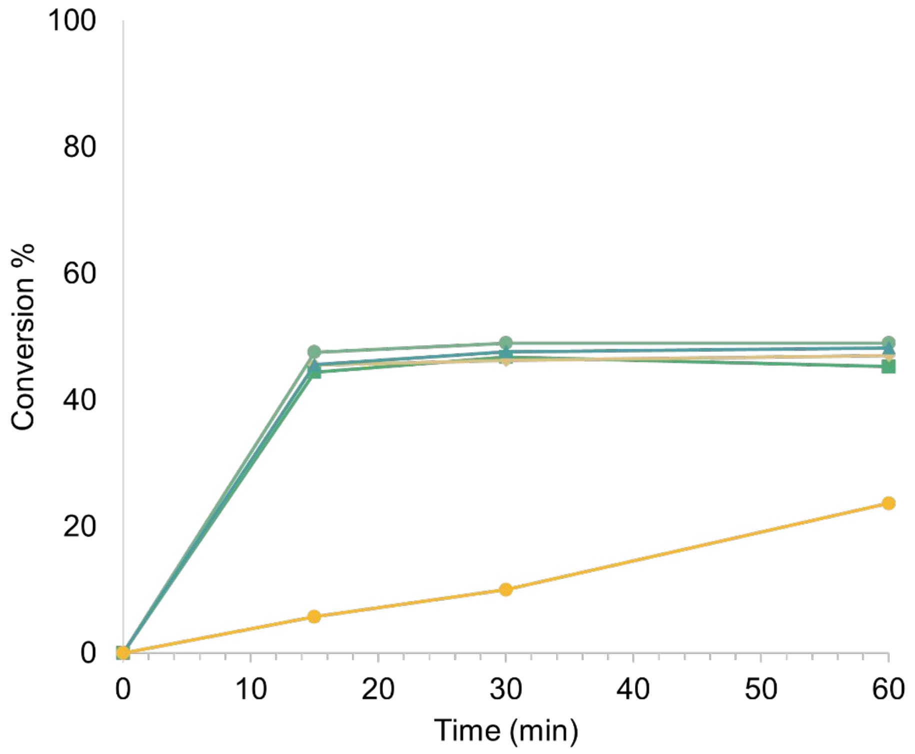

Figure S31. Conversion of the reaction between hexanol and **AllOx** against time of a variety of catalyst: MSA (green traces) and TFA (yellow traces) in two different concentrations: 1mol% (triangles and squares) and 0.1 mol% (circles).

S8. Exchange Reaction

Exchange reactions between **4** and methanol were carried out. The two components were mixed in an argon-filled glovebox with a ten-fold excess of methanol to **4**. The reaction was carried out in dry $CDCl_3$ using TFA (0.1mol%) as catalyst. Aliquots of the reaction mixture were sampled over time. The reactions were monitored by ^{1}H-NMR spectroscopy to determine the conversion in the product.

For all reaction the conversion was calculated with Equation 2 (where I_t is the integral at time t and 3 is the value of the integral when 100% exchange occurred) by taking into consideration the singlet of the CH_3 of **1** (3.18 ppm). The signals were normalized using the signal of the allyl bond (multiplet, 5.16 ppm).

$$Conversion\ (\%) = \frac{I_t}{3} * (2)$$

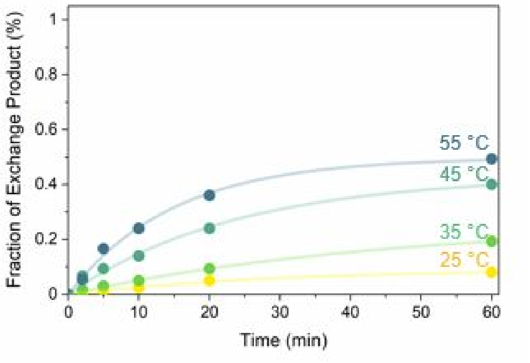

Figure S32. Fraction of exchanged product calculated from ^{1}H NMR of the exchange reaction between **4** and methanol at temperature between 25 and 55 °C

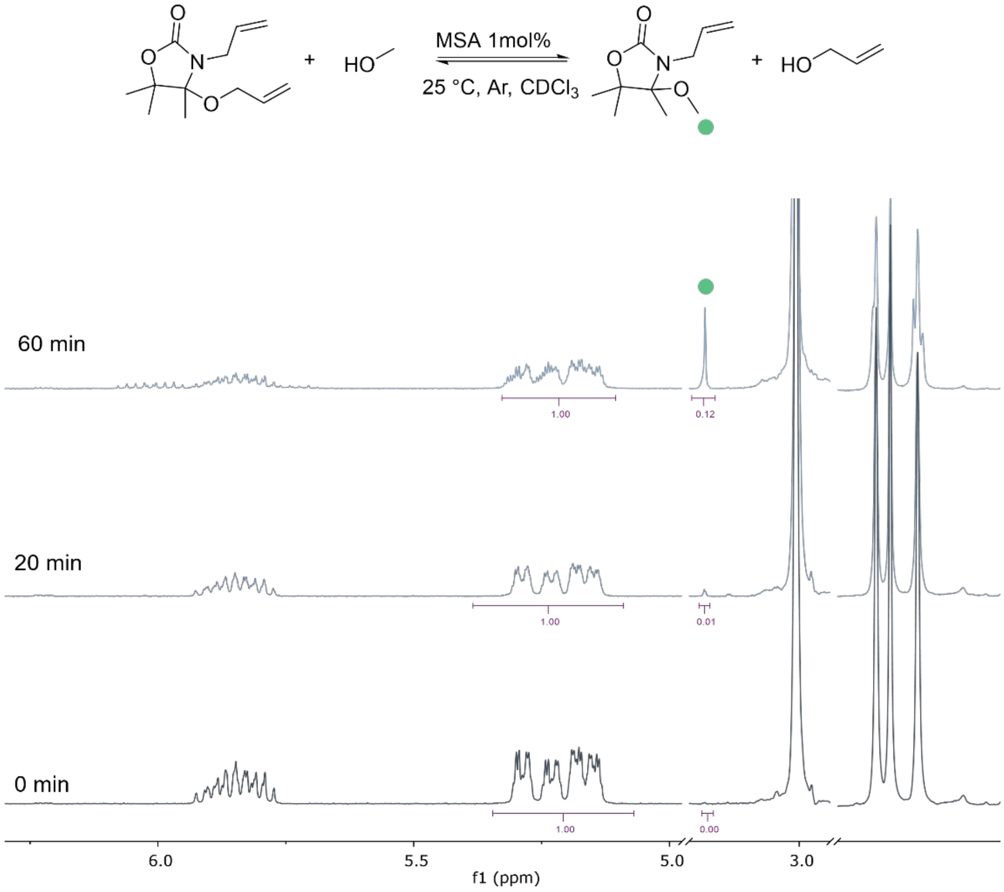

Figure S33. ^{1}H NMR kinetics of the exchange reaction between **4** and methanol at 25 °C

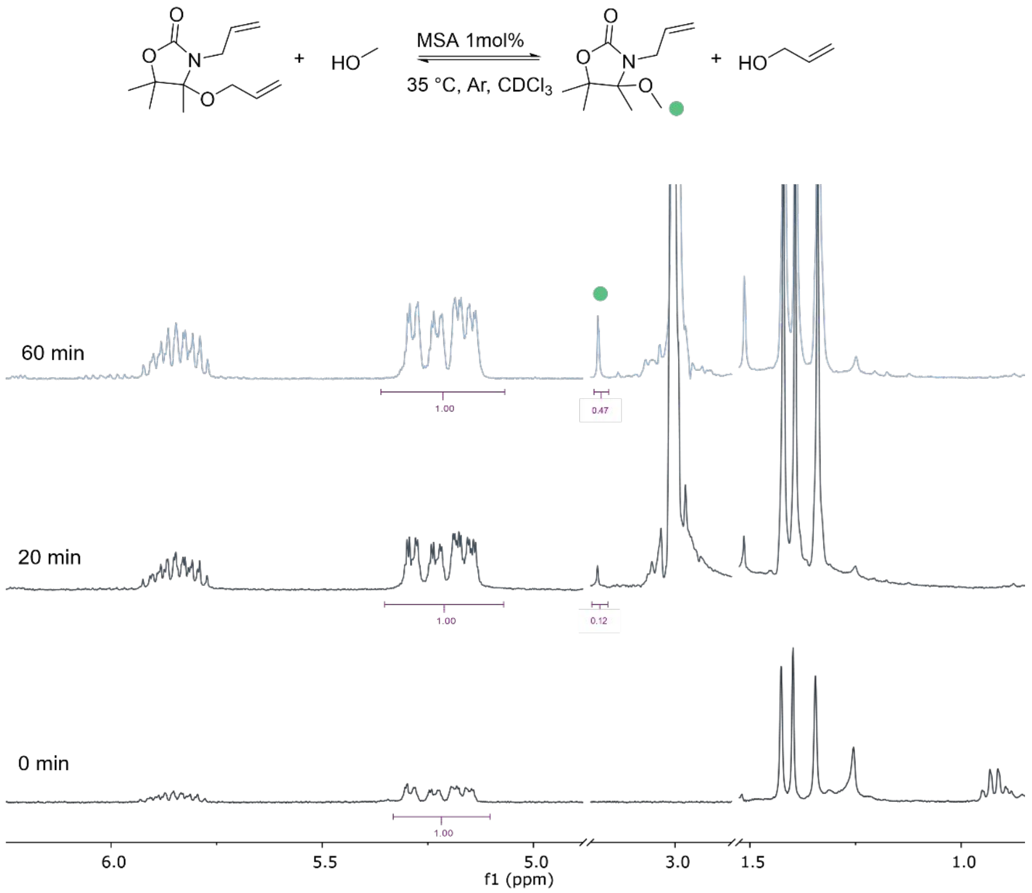

Figure S34. ^{1}H NMR kinetics of the exchange reaction between **4** and methanol at 35 °C

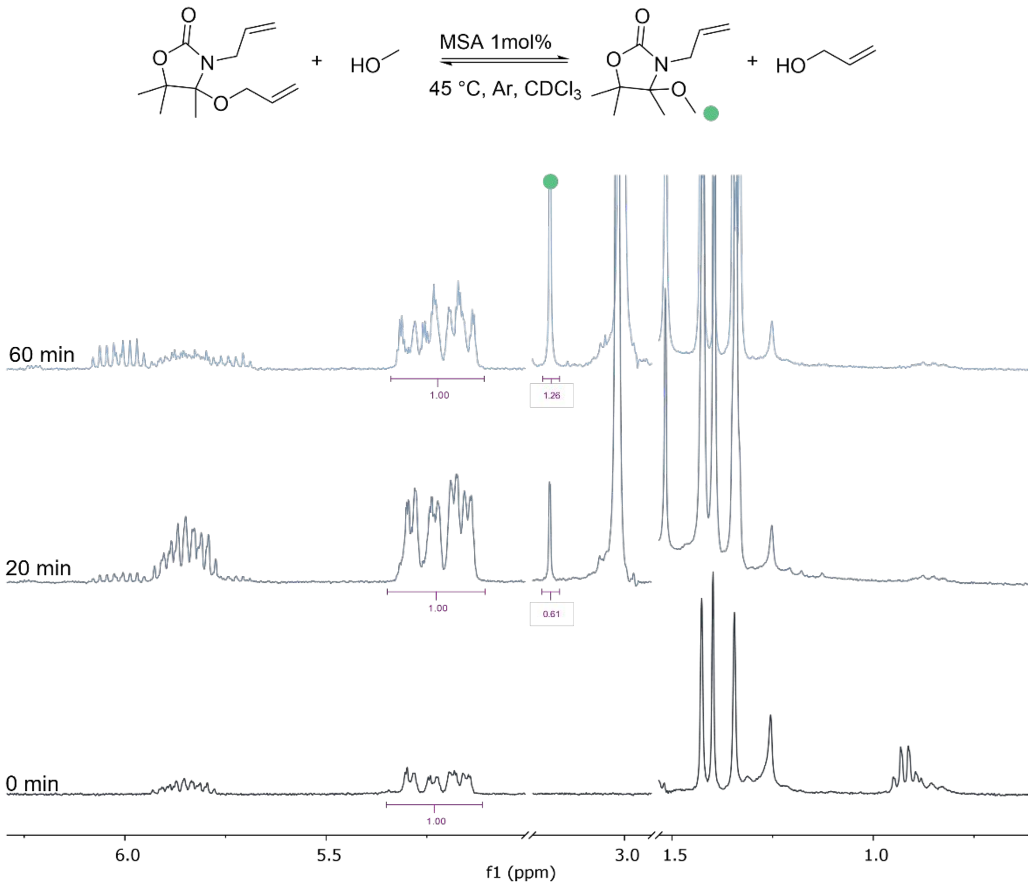

Figure S35. ^{1}H NMR kinetics of the exchange reaction between **4** and methanol at 45 °C

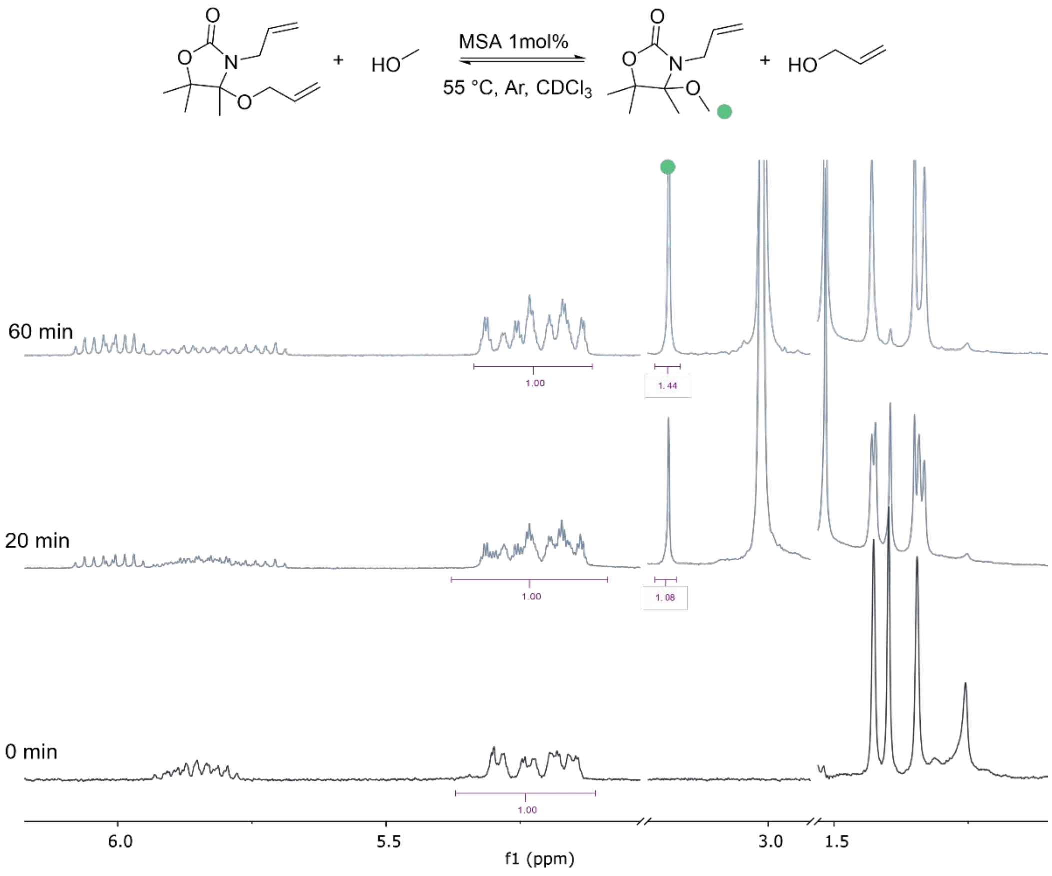

Figure S36. ^{1}H NMR kinetics of the exchange reaction between **4** and methanol at 55 °C

Table S2 Fitting parameters of exchange kinetics of **4** and methanol at different temperatures

**T**	**R^2**
25 °C	0.991
35 °C	0.992
45 °C	0.979
55 °C	0.994

S9. Materials preparation

Mixture of **AllOx** and **4** was mixed with TEA (if applicable, 0.5 wt%). After homogenisation, a stoichiometric amount of crosslinker together with BAPO (0.5 wt%) were added. The clear, pale-yellow mixture was casted onto silicone moulds before being irradiated with light (390 nm, 20 mW/cm^2). After 60 second the films were demoulded and stored in a desiccator before testing.

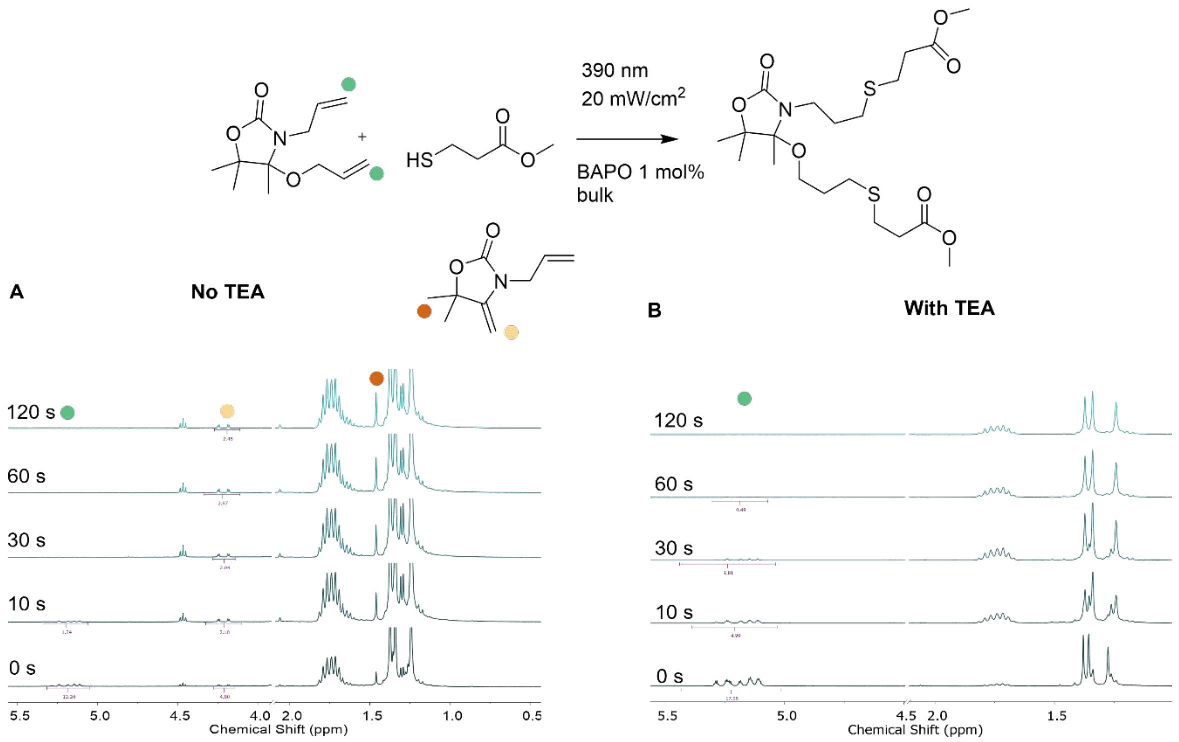

Figure S37. ^{1}H NMR kinetics in DMSO-d_6 of thiol ene kinetics between **4** and 3-methyl-mercaptopropionate with (a) and without TEA (b) (reaction conditions: bulk, 25 °C, stoichiometric under ambient conditions. Light 390 nm at 20 mW/cm^2. BAPO 1 mol%, TEA 1 mol%)

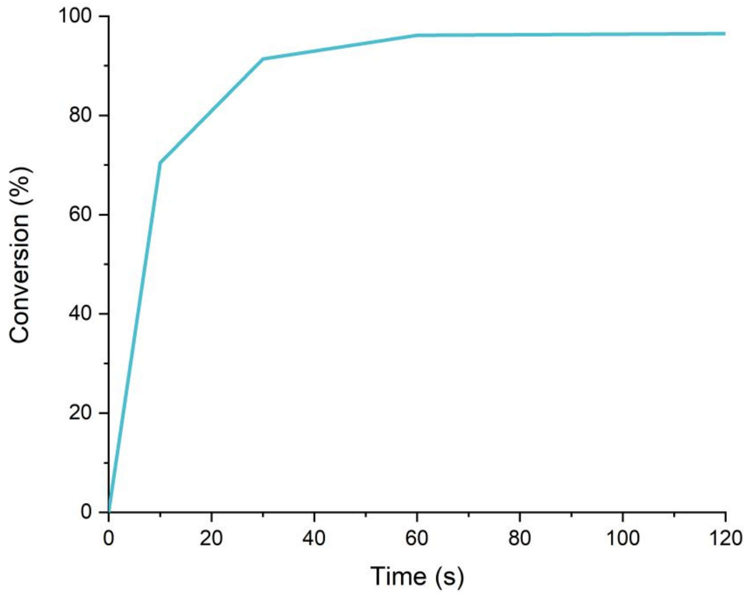

Figure S38. Conversion over time of thiol ene reaction between **4** and 3-methyl-mercaptopropionate with TEA. The conversion was calculated with Equation 1 (where I_t is the integral at time t and I_0 the integral at time 0) by taking into consideration the signal of the CH_2 of Allyl double bond (5.2 ppm, m). The signals were normalized using the signal of CH_3 (1.4 ppm, s).

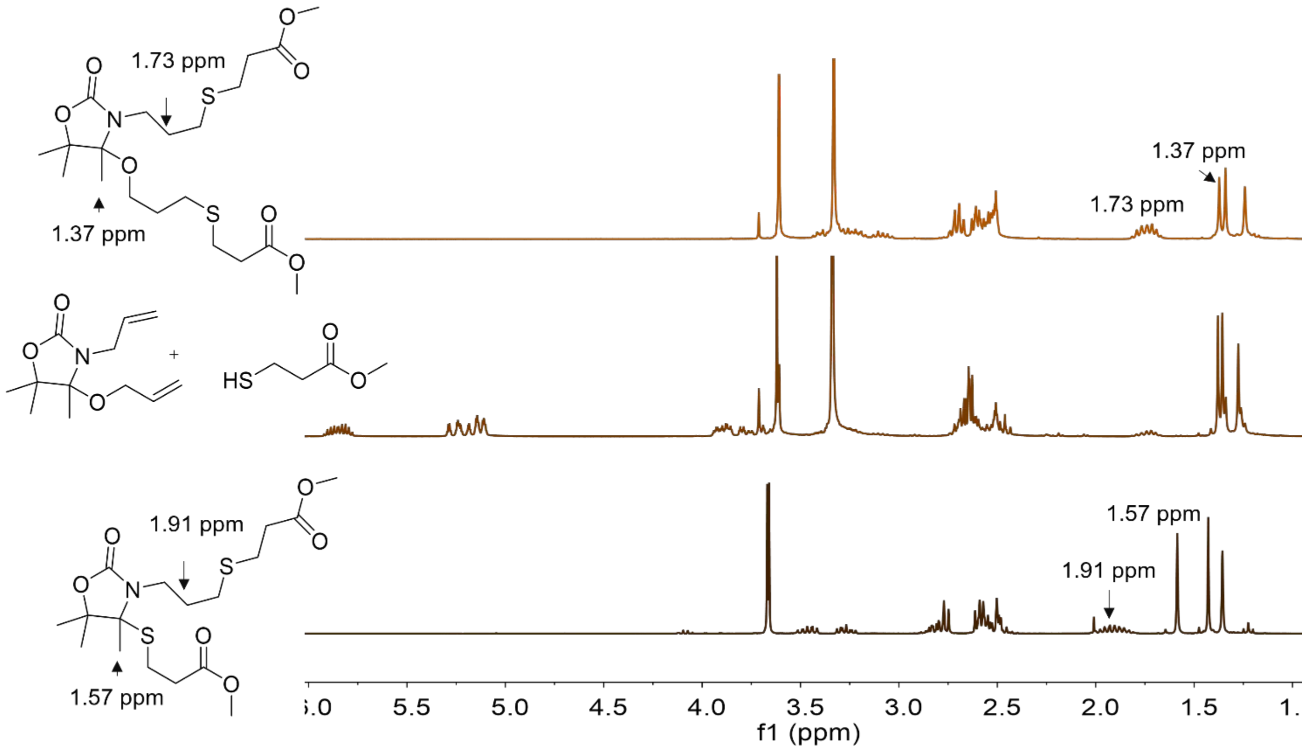

Figure S39. ^{1}H-NMR spectra of a model N,S acetal compound (taken from ref 9)[9], mixture of **4** and 3-methyl-mercaptopropionate pre and post thiol-ene reaction confirming that there is no formation of N,S-acetal moieties during the photocuring procedure.

Table S3 Thermomechanical properties of materials prepared in this study

Entry	Sample	Swelling (THF, %)	Gel Content (THF, %)	Tg (DSC)	Endothermic peak (DSC)	Young's Modulus (Mpa)	Elongation at Break (%)	Stress at Break (MPa)
1	P(S3,0)	195±2	97.0±0.3	9	-	8±0.3	75±9	3.9±0.2
2	P(S4,0)	146.2±0.5	98±0.3	24.8	-	737±26	28.1±2.1	11.9±1.2
3	P(S6,0)	142±5	97±3	41	-	1720±70	4±0.4	27±0.9
4	P(S4,25)	151±3	98.2±0.3	19.5	145	n.d.	n.d.	n.d.
5	P(S3,50)	205±0.7	90.7±0.8	-0.2	154.6	3.0±0.2	45.5±1.5	1.3±0.2
6	P(S4,40)	140±3	96±2	17	149	n.d.	n.d.	n.d.
7	P(S4,50)	149±2	94±2	2	164	2.8±0.2	60.8±2.9	1.77±0.2
8	P(S6,50)	155.3±0.9	98.0±0.1	18	169	27.4±1.5	47.2±2.3	11.4±1.1
9	P(S4,60)	151±3	96.2±0.8	9	160	n.d.	n.d.	n.d.
10	P(S4,75)	161±2	95.5±0.4	4.2	141	n.d.	n.d.	n.d.
11	P(S3,100)	172.4±2.5	94.7±0.8	1.4	145	n.d.	n.d.	n.d.
12	P(S4,100)	135±2	95±2	8.8	106	10.4±0.3	64.3±3.2	5.97±0.5
13	P(S6,100)	146.3±1.5	97.2±0.5	19.5	99	n.d.	n.d.	n.d.

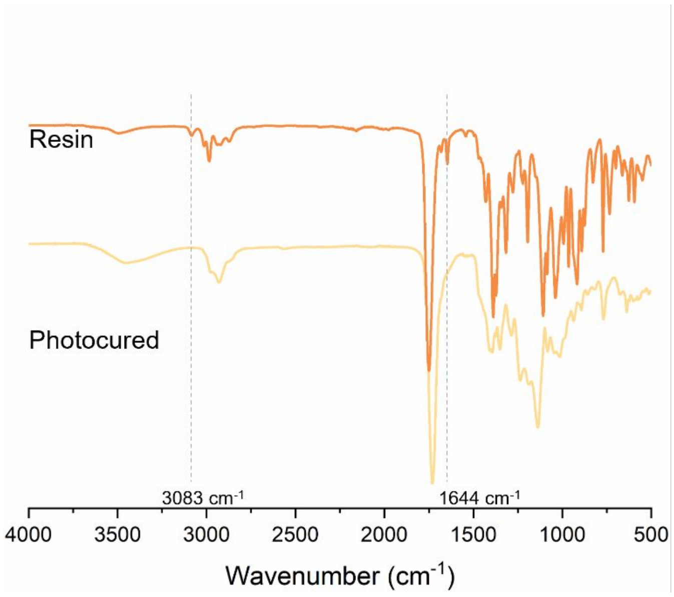

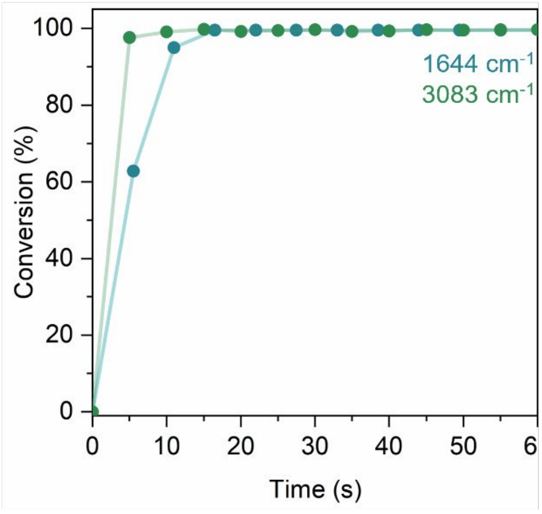

Figure S40. FTIR of P(S4,100) pre and post curing (60 s, 20 mW/cm^2, 390 nm), Conversion calculated from Real time FTIR monitoring of photo curing of P(S4,100) by monitoring the resonance at 1644 cm^{-1} and at 3083 cm^{-1}

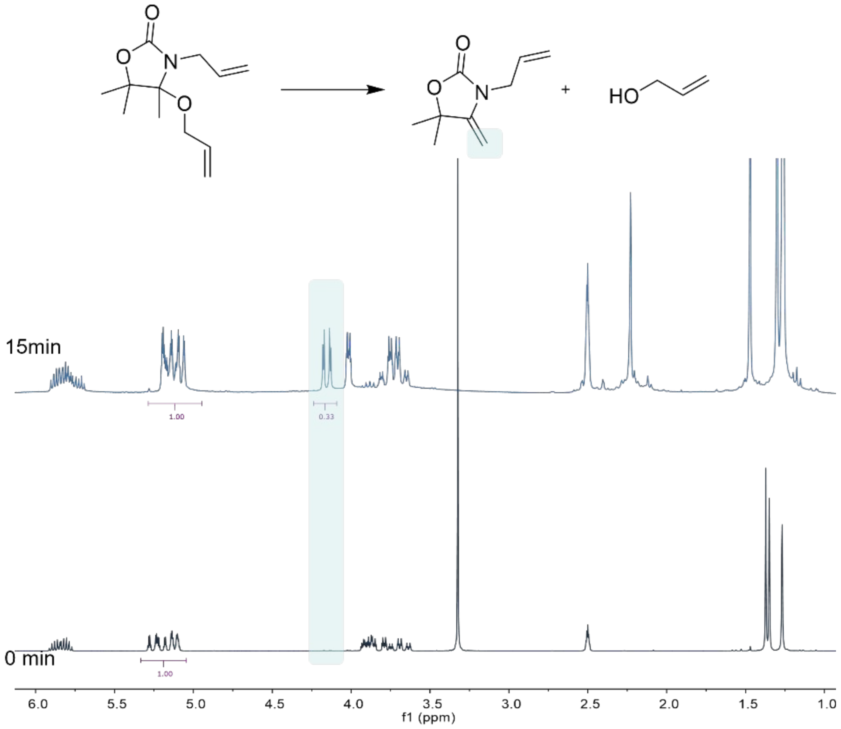

Figure S41. Reaction between **4** and BAPO photodegradation products (reaction conditions: stoichiometric, 25 °C, bulk, Argon atmosphere)

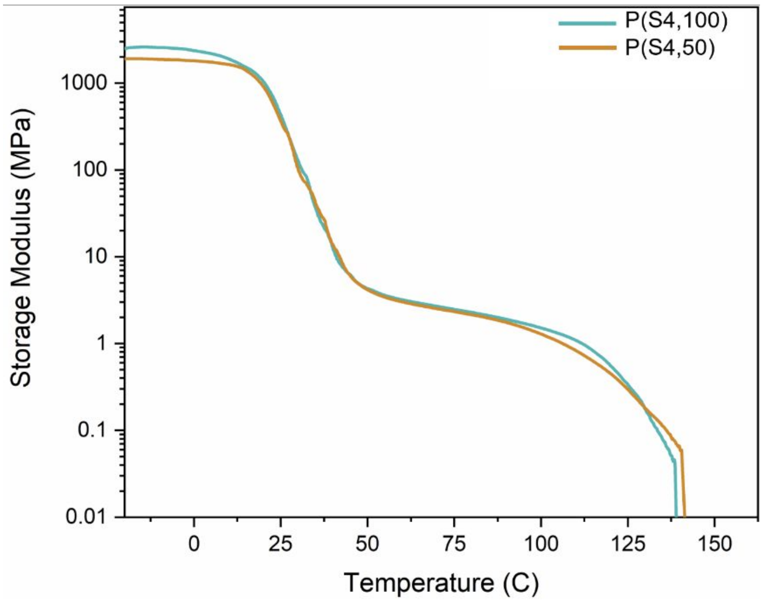

Figure S42. DMA trace of **P(S4,50)** and **P(S4,100)**

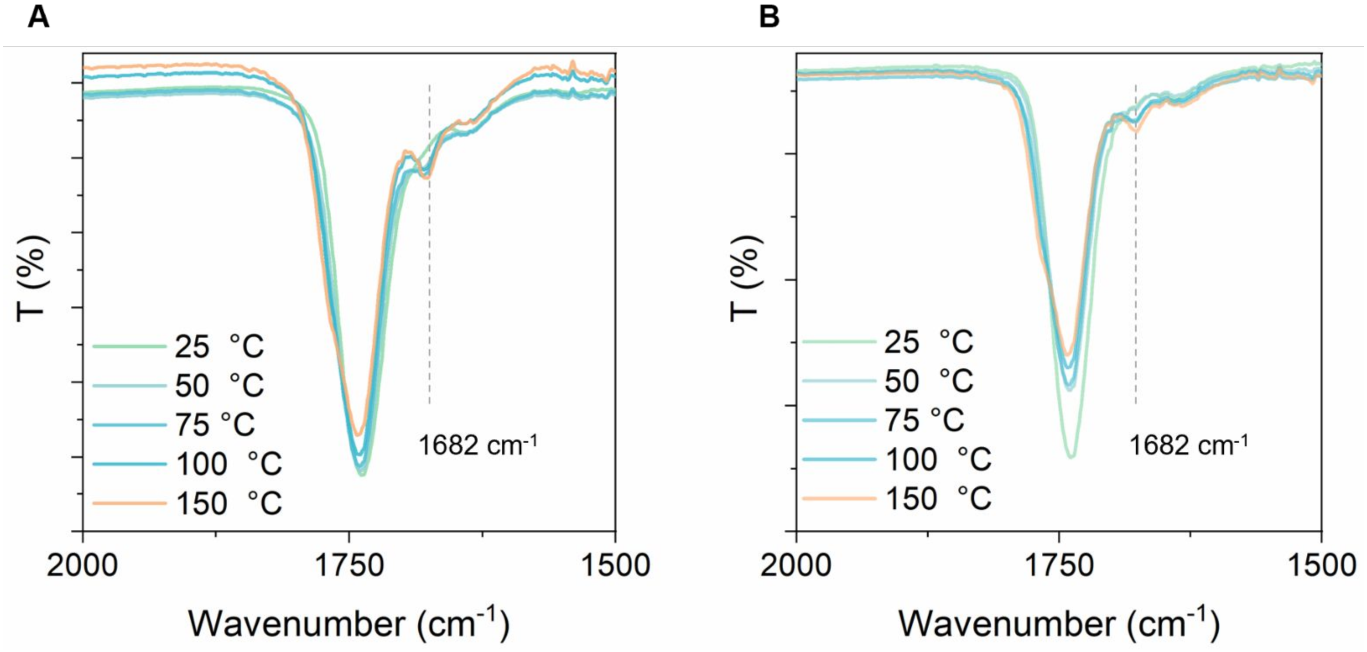

Figure S43. Temperature dependant FTIR spectra of **P(S4,100)** (a) and **P(S4,50)** (b) at temperature ranging from 25 to 150 °C

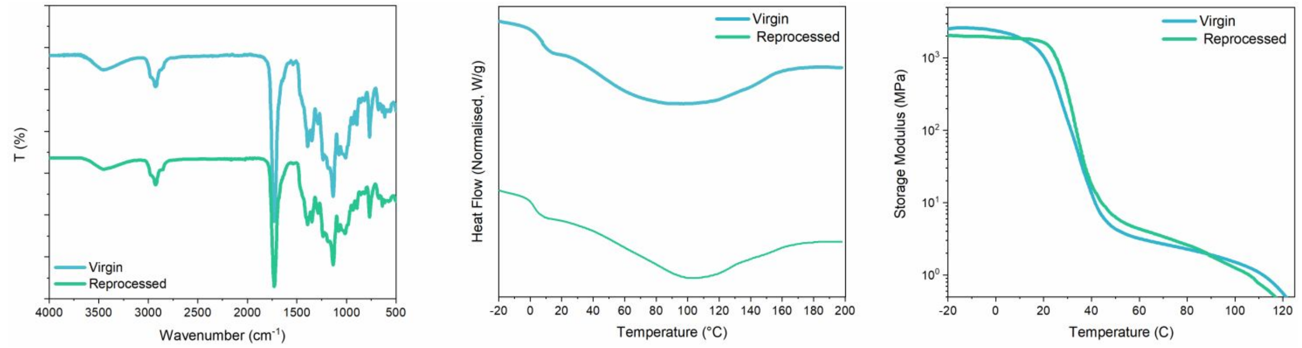

Figure S44. IR spectra, DSC and DMA trace of virgin and reprocessed **P(S4,50)**

S10. Hydrolytic Stability of Model Compounds

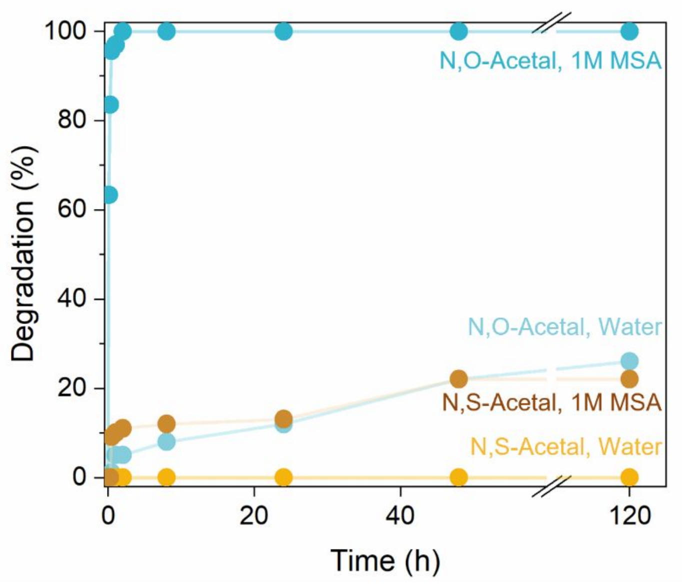

Figure S45. Kinetics of the hydrolysis of **4** at 25 °C and **6**. (0.2 M D_2O/H_2O, watergated ^1H NMR, 1mol% MSA for acid environment)

Hydrolytic degradation of a *N*,*O*-acetal Model Compound

4 was mixed with H_2O (0.2M, 1 mol% MSA) and stirred vigorously. Aliquots were samples and immediately quenched with TEA before being diluted in D_2O:H_2O (9:1) and their $^1H_{watergate}$ NMR spectra being measured. The conversion was calculated on the ratio between methyl signal of **4** and its hydrolysis products (1.41, 1.37 ppm) using Equation 1

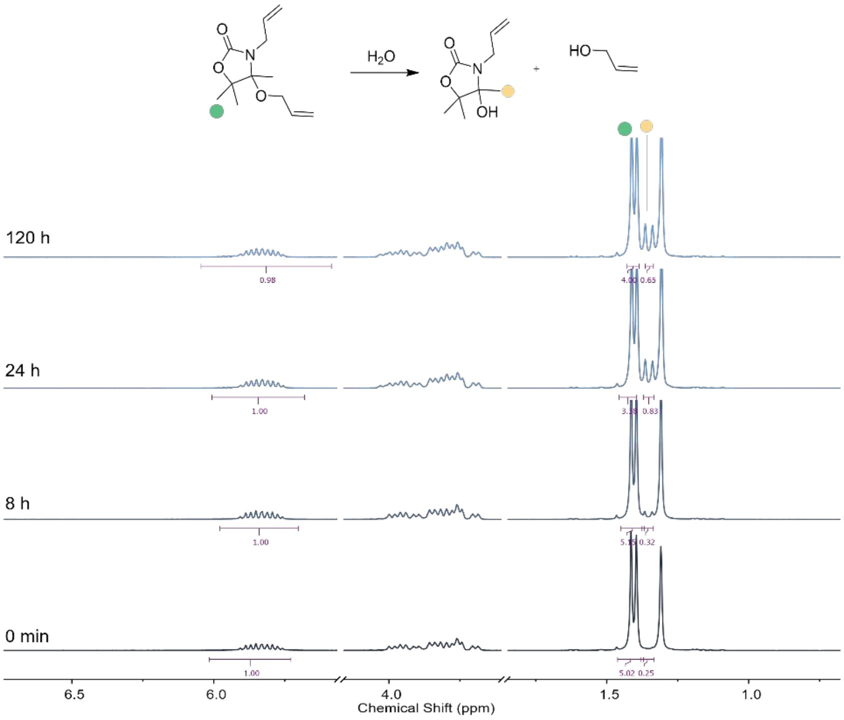

Figure S46. ^{1}H NMR kinetics of the hydrolytic degradation of **4** at 25 °C (0.2 M D_2O/H_2O, watergate ^{1}H NMR)

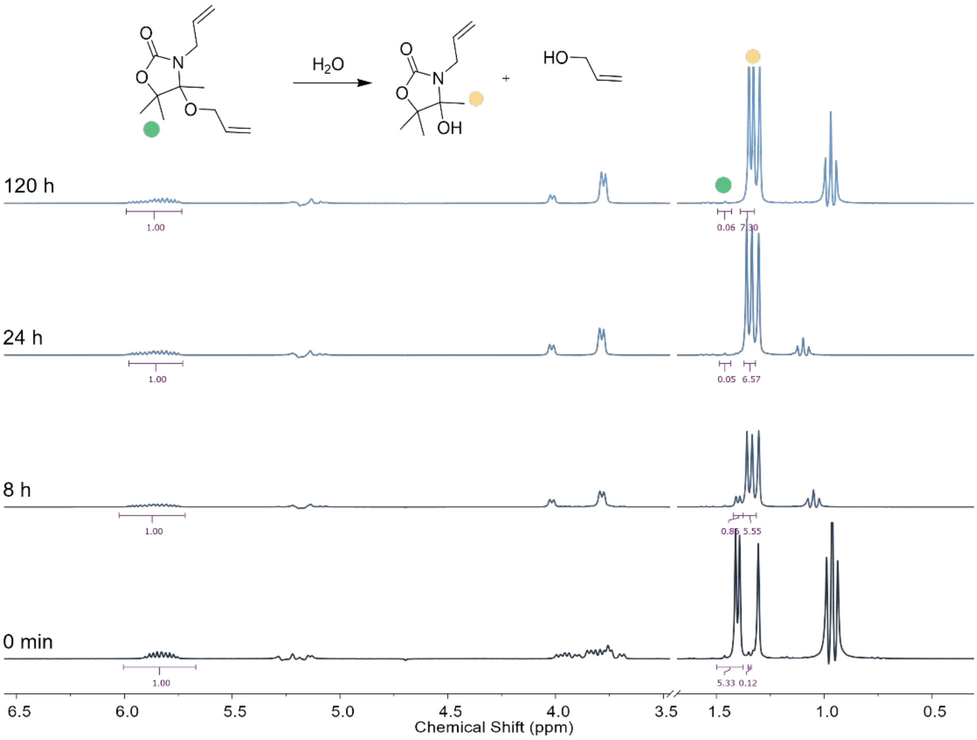

Figure S47. ^{1}H NMR kinetics of the hydrolytic degradation of **4** at 25 °C with MSA 1 mol% (0.2 M D_2O/H_2O, TEA quench 10 uL, watergate ^{1}H NMR)

Hydrolytic degradation of a *N*,*S*-acetal Model Compound

6 was mixed with water (0.2M) and stirred vigorously. Aliquots were samples and immediately quenched with TEA before being diluted in DMSO-d_6 and their ^{1}H NMR spectra being measured. The conversion was calculated on the reduction of the singlet at 1.46 ppm (methyl group of AllOx) using Equation 1.

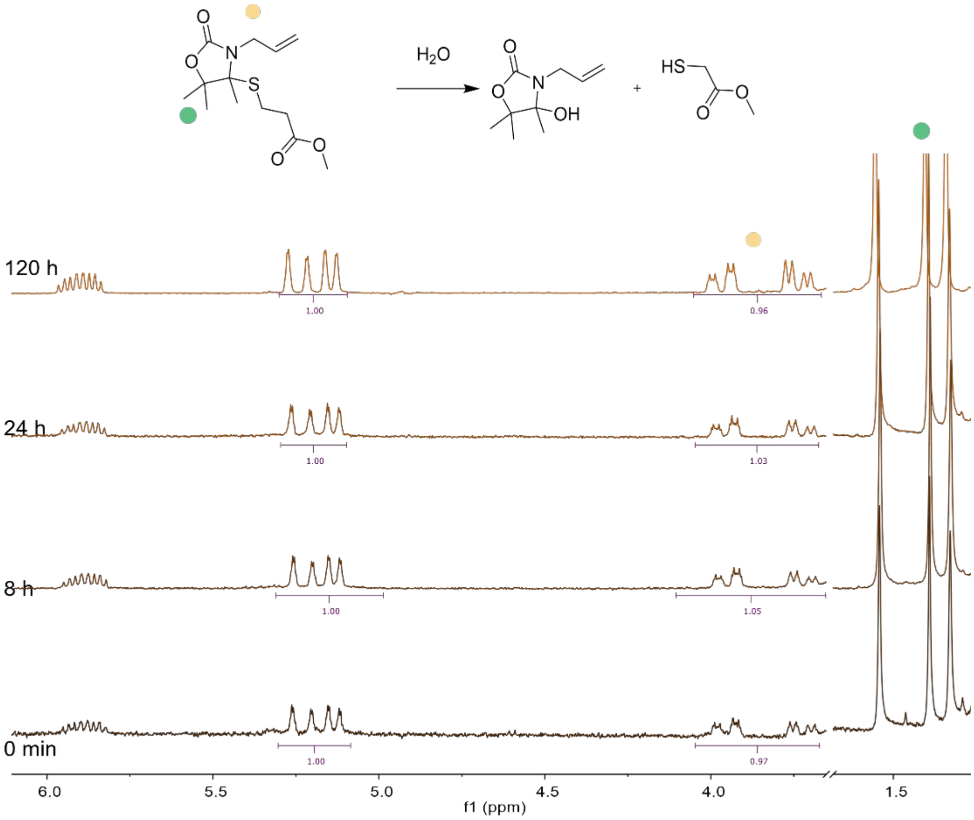

Figure S48. ^{1}H NMR kinetics of the hydrolytic degradation of **5** at 25 °C (0.2 M in H_2O, quench TEA 10 uL, DMSO-d_6 0.5 mL)

6 was mixed with water (0.2M, 1 mol% MSA) and stirred vigorously. Aliquots were samples and immediately quenched with TEA before being diluted in DMSO-d_6 and their ^{f}H NMR spectra being measured. The conversion was calculated on the reduction of the singlet at 1.46 ppm (methyl group of AllOx) using Equation 1.

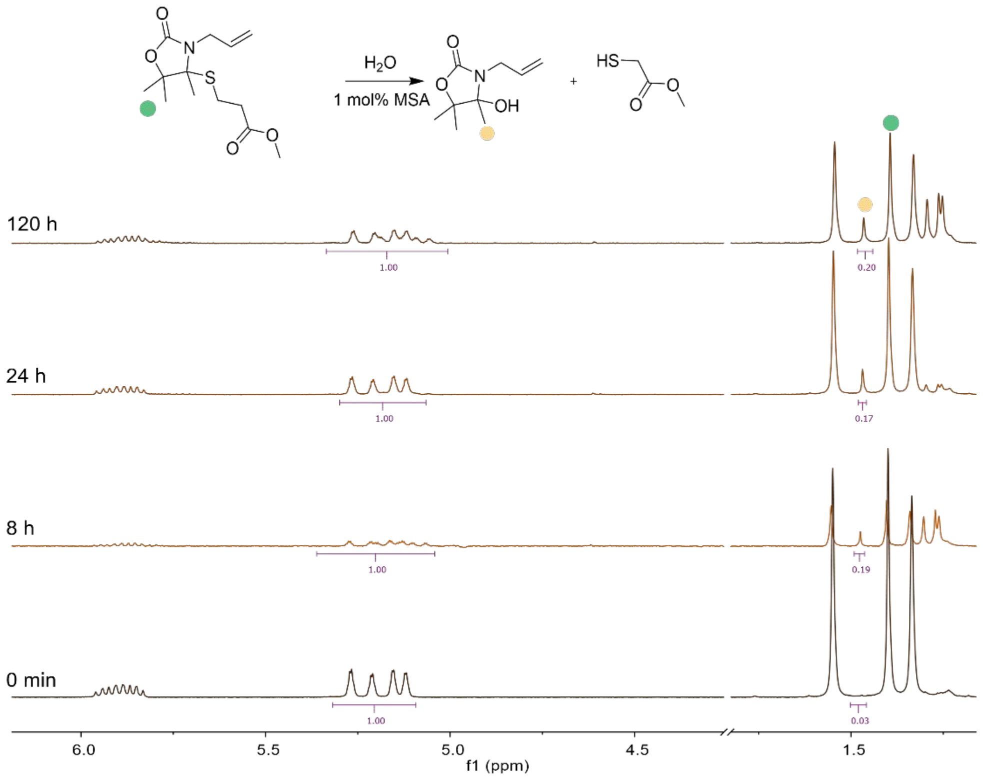

Figure S49. ^{1}H NMR kinetics of the hydrolytic degradation of **5** at 25 °C with MSA 1 mol% (0.2 M in H_2O, quench TEA 10 uL, DMSO-d_6 d6 0.5 mL)

Hydrolytic degradation of an O,O-acetal Model Compound

1,1-Dimethoxyethane was mixed with water (0.2M) and stirred vigorously. Aliquots were samples and immediately quenched with TEA before being diluted in DMSO-d_6 and their 1H NMR spectra being measured. The conversion was calculated on the disappearance of multiplet at 4.5 ppm. For all reaction the conversion was calculated with Equation 1.

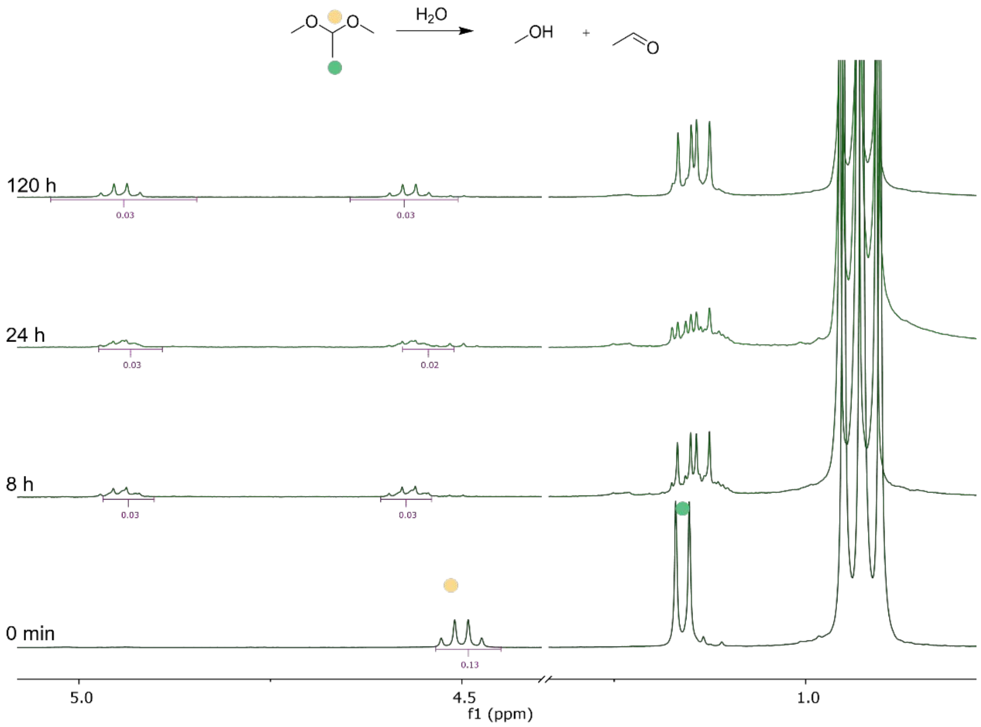

Figure S50. ^{1}H NMR kinetics of the hydrolytic degradation of 1,1-Dimethoxyethane at 25 °C (0.2 M in H_2O, quench TEA 10 uL, DMSO-d_6 0.5 mL)

1,1-Dimethoxyethane was mixed with water (0.2M, 1 mol% MSA) and stirred vigorously. Aliquots were samples and immediately quenched with TEA before being diluted in DMSO-d_6 and their 1H NMR spectra being measured. The conversion was calculated on the disappearance of multiplet at 4.5 ppm.

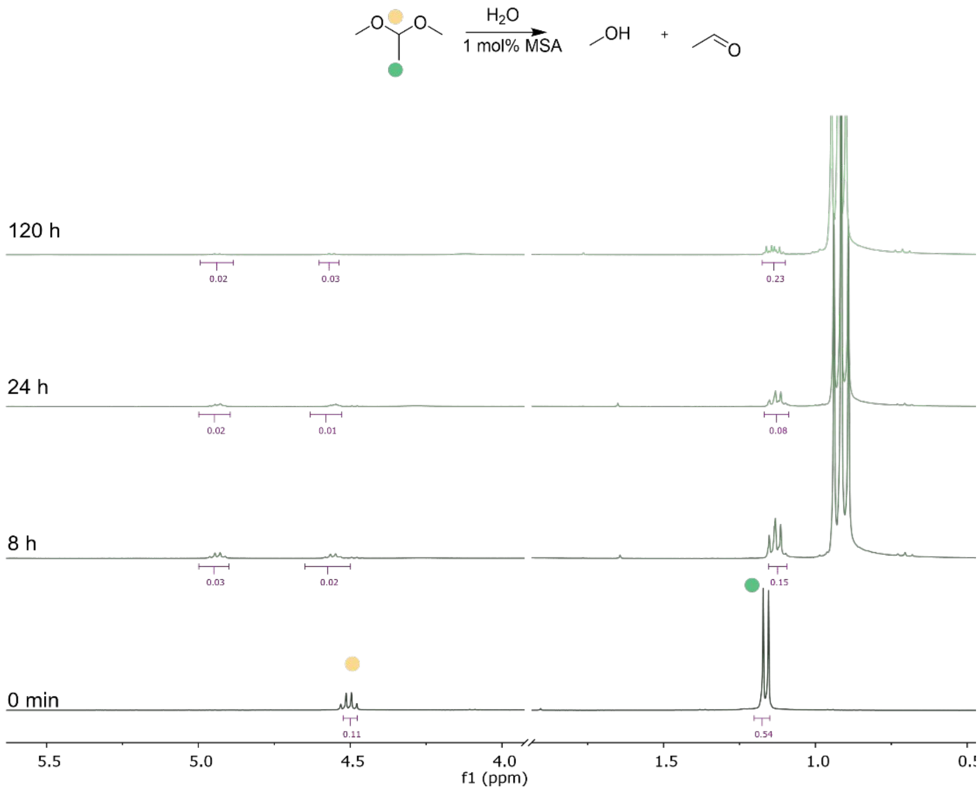

Figure S51. ^{1}H NMR kinetics of the hydrolytic degradation of 1,1-Dimethoxyethane at 25 °C with MSA 1 mol% (0.2 M in H_2O, quench TEA 10 uL, DMSO-d_6 0.5 mL)

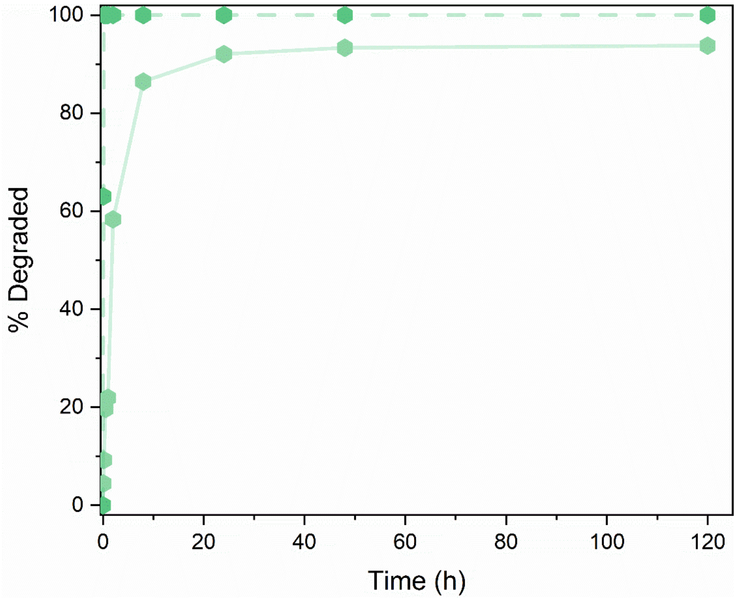

Figure S52. Degradation in water (solid lines) and 1M MSA (dashed lines) of an O,O-acetal model compound (1,1-Dimethoxyethane).

DFT Modelling of Hydrolytic Degradation of a *N,O*-acetal Adduct

DFT calculations of geometries, energies and vibrational frequencies reported in this paper were carried out with the ωB97X-D functional [1] using the 6-311++G(d,p) basis set with the CPCM solvation model (chloroform) n the Gaussian 16 package. All frequencies of each structure have also been calculated to verify the presence of a single imaginary frequency for transition states and the absence of imaginary frequency for ground states.

In a first step, the acidic catalyst (MSA) protonates the oxygen of the *N*,*O*-acetal moiety giving origin to TS1 (ΔG=24.6 kJ/mol). This evolves into INT1, with strong H-Bonding interactions between water, the alcoholic moiety and the acidic catalyst (ΔG=-11.1 kJ/mol). Following, water attacks the as-formed carbocation, originating TS2 (ΔG=7.7 kJ/mol). Deprotonation of the Hydroxyoxazolidone moiety restitutes the product with a stabilization of -8.7 kJ/mol when compared to the initial N,O-acetal derivative.

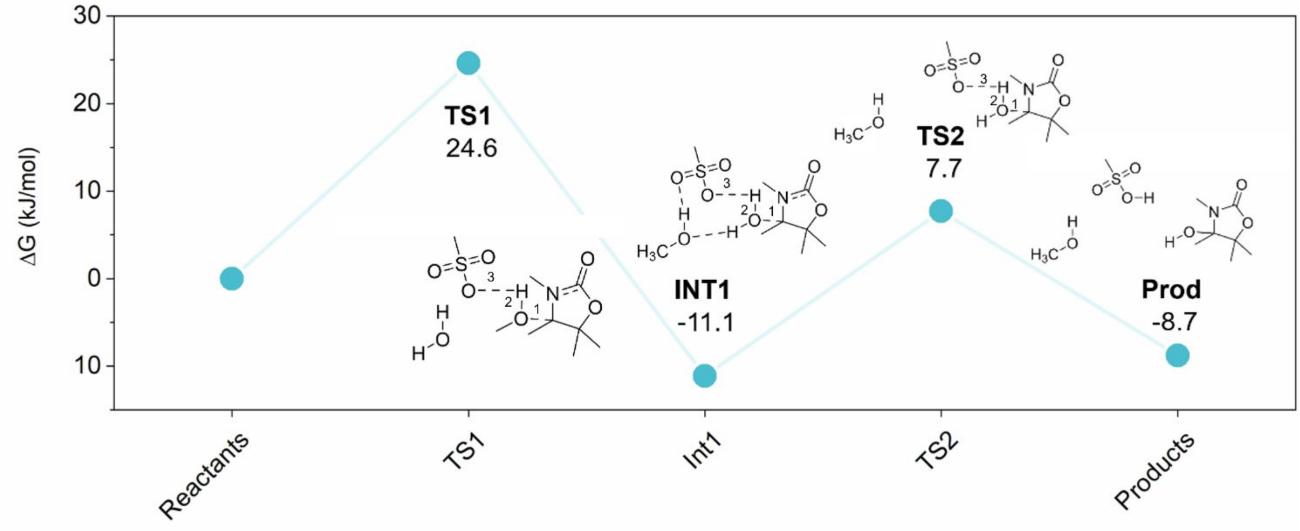

Figure S53. Gibbs-free energy profile of the reaction pathway for the hydrolytic degradation of the *N,O*-acetal moiety catalysed by MSA.

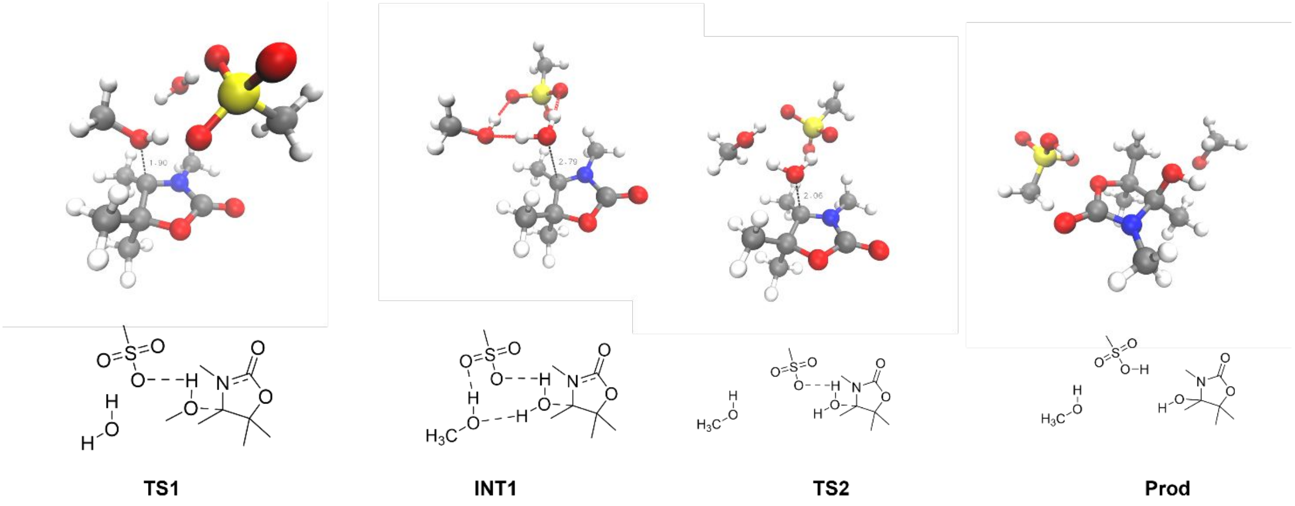

Figure S54. 2D and 3D structures of optimised intermediate and transition states of the modelled reaction path of hydrolysis of the *N,O*-acetal moiety

Table S4 Bond Length evolution along the reaction path for intermediates and transition states.

	Label	TS1	Int1	TS2
C_{mon}--O_{mon}	1	1.89979	2.79281	2.06211
-O_{mon}--H-	2	1.02191	0.97311	0.98683
-H--O_{cat}-	3	1.47463	1.83196	1.71255

Determination of degradation kinetics in materials

Circular films of the material (~250 mg) were placed in water or a 1M solution of MSA. Their degradation % was determined gravimetrically measuring the weight loss over time.

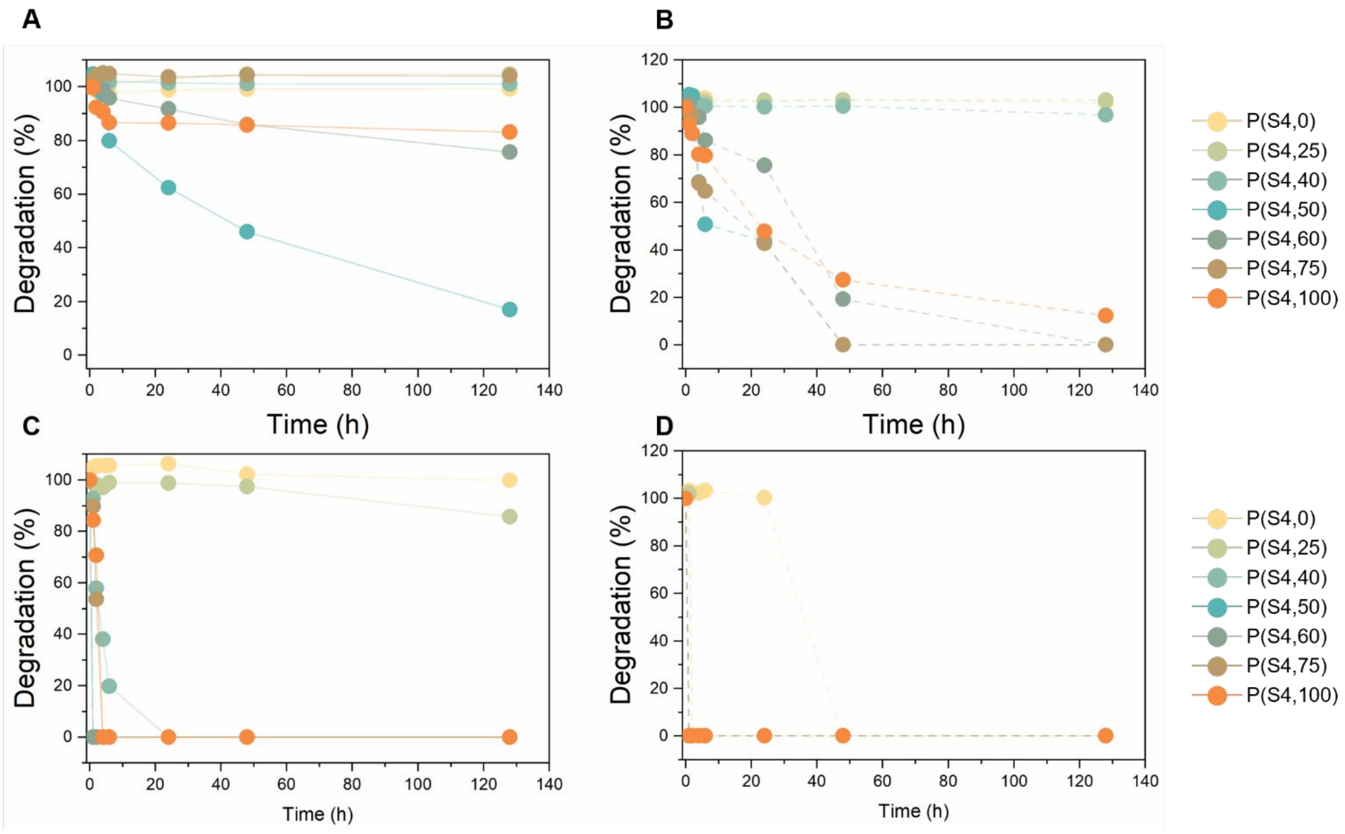

Figure S55. Hydrolytic degradation of materials ranging from **P(S4,0)** to **P(S4,100)** under neutral (a) and acidic conditions at 25°C (b). Hydrolytic degradation of materials ranging from **P(S4,0)** to **P(S4,100)** under neutral (c) and acidic conditions at 100°C (d).

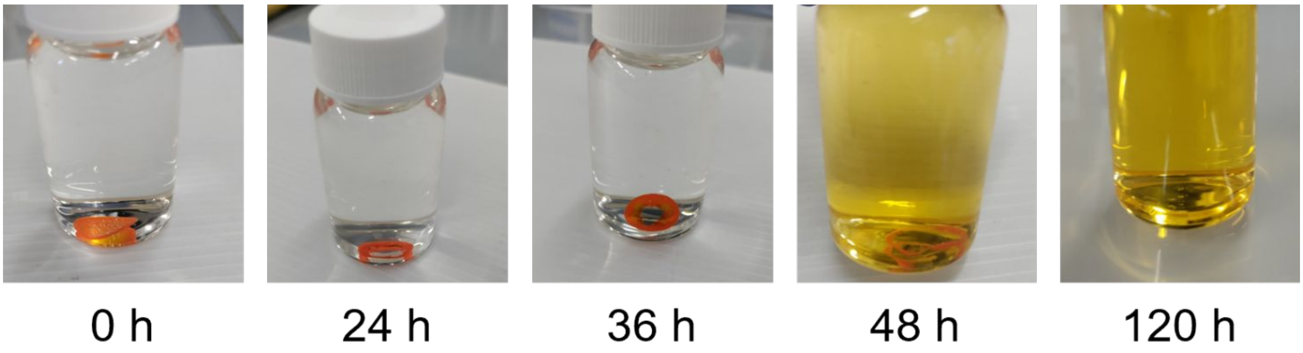

Figure S56. P(S4,50) degradation in 1M MSA (a water-soluble dye was added to the material to improve visibility)

3D Printing

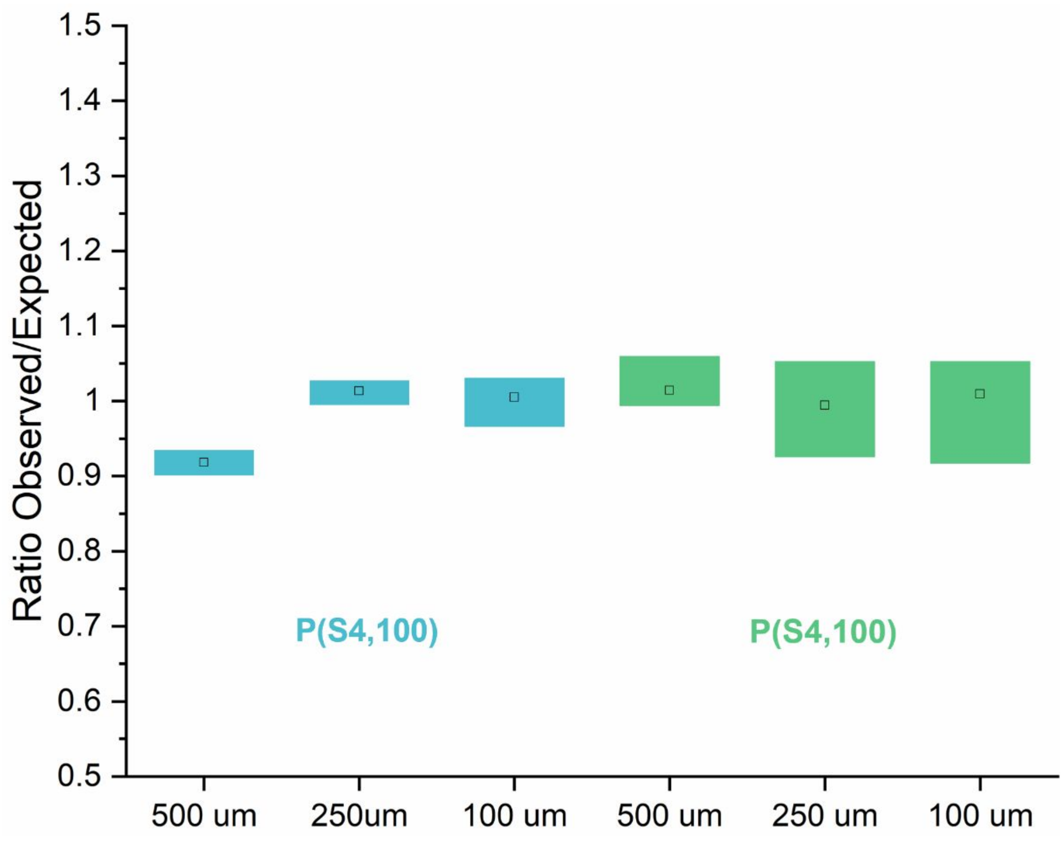

Figure S57. SEM measurement of grooves in structures for resolution studies with their relative size and distribution from the expected printed feature for **P(S4,100)** and **P(S4,50)**. Box shows the 25-75 percentile. Target size of 500 μm, 250 μm, and 100 μm.

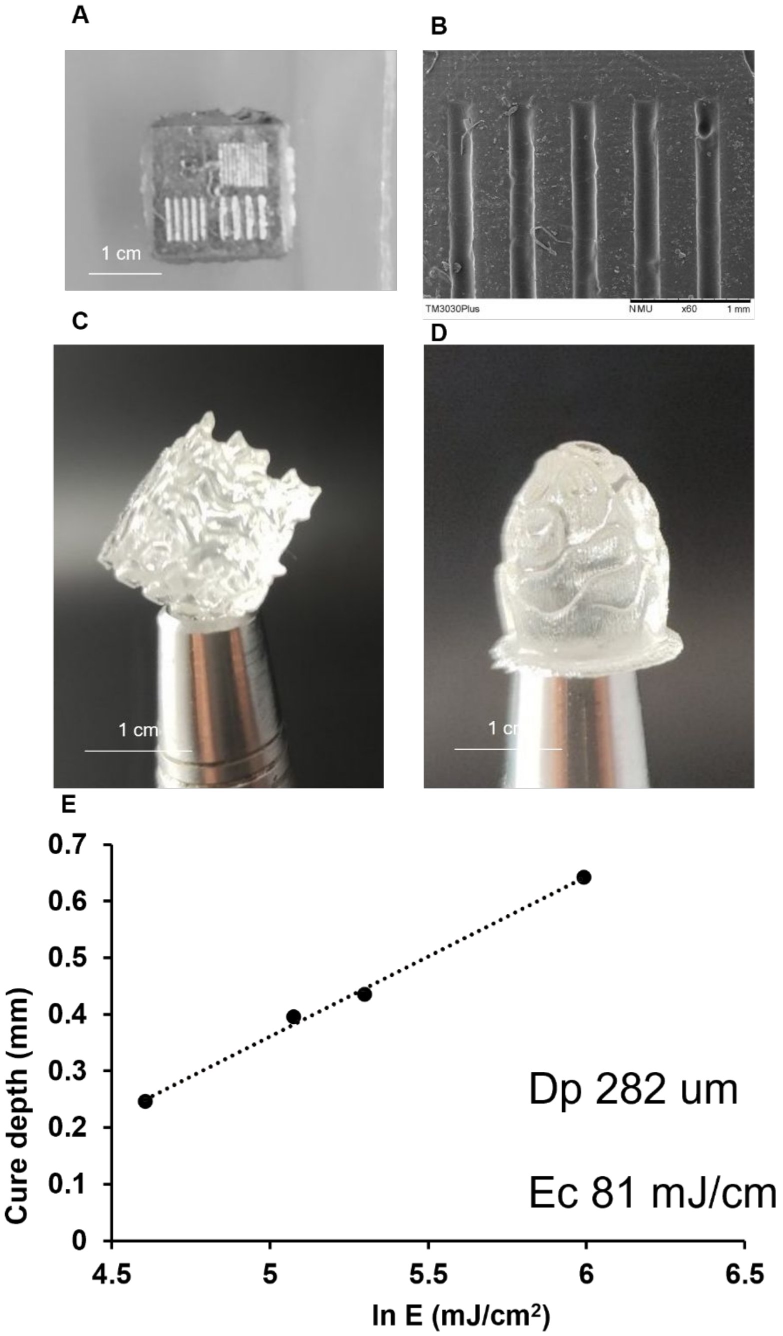

Figure S58. a) 3D printed structure for optimisation printed using **P(S4,50)** (390 nm, 20 mW/cm^2) ; b) SEM image showing 100 µm lines; c,d) 3D printed gyroid cube and voronoy egg printed using **P(S4,50)** (390 nm, 20 mW/cm^2) ; e) Jacobs working curve of **P(S4,50)** (BAPO 0.5 wt%, TEA 0.5 WT%, 390 nm, 20 mW/cm^2)

DFT ωB97-XD/6-311++G(d,p) Optimized Cartesian Coordinates

INT1
36

C	-2.424513	0.710220	0.907834
O	-2.753242	1.730800	1.454530
O	-2.952304	0.254568	-0.242599
N	-1.483582	-0.207132	1.358438
C	-2.340063	-0.997420	-0.630794
C	-3.419598	-2.075391	-0.594875
H	-4.217457	-1.821416	-1.298971
H	-2.993172	-3.042956	-0.876952
H	-3.848237	-2.161641	0.408009
C	-1.726982	-0.822440	-2.013791
H	-1.236133	-1.750917	-2.324568
H	-2.514398	-0.588284	-2.736987
H	-0.995091	-0.010697	-1.997284
C	-1.297006	-1.215785	0.452781
C	-0.640559	0.044515	2.504331
H	0.387484	0.248606	2.187864
H	-1.039009	0.914855	3.026456
H	-0.648354	-0.822220	3.172192
C	-0.381028	-2.201416	0.513643
H	0.278657	-2.318034	1.368234
H	-0.387030	-2.990378	-0.231843
H	1.043555	-1.204251	-0.443461
C	-0.701039	2.967691	-0.777113
H	-0.762388	3.474487	0.193484
H	-0.118484	3.582026	-1.477052
H	-1.715532	2.852248	-1.164886
S	2.821142	-0.227437	0.286526
O	2.441954	1.175706	0.427767
O	1.913371	-0.910504	-0.837789
O	-0.155904	1.665906	-0.652848
H	0.738882	1.720222	-0.282584
O	2.833330	-1.056832	1.479817
C	4.384002	-0.316332	-0.550819
H	5.115057	0.168368	0.098909
H	4.637648	-1.365440	-0.704700
H	4.300462	0.215964	-1.498999

INT2A
36

C	-2.208165	0.797704	0.788235
O	-2.510481	1.768626	1.406149
O	-2.798320	0.290490	-0.286599
N	-1.183440	-0.151901	1.205227
C	-2.188092	-0.959391	-0.670026
C	-3.283808	-2.022129	-0.688419
H	-4.061903	-1.706612	-1.387868

H	-2.881616	-2.979316	-1.030430
H	-3.735365	-2.152289	0.299903
C	-1.486307	-0.792126	-2.017929
H	-0.972437	-1.718114	-2.290216
H	-2.244166	-0.565091	-2.773126
H	-0.757786	0.019635	-1.967943
C	-1.180287	-1.185642	0.428270
C	-0.335707	0.175451	2.349140
H	0.053892	1.180179	2.178912
H	-0.945687	0.154373	3.254246
H	0.508709	-0.512668	2.388348
C	-0.343922	-2.386588	0.535836
H	0.091712	-2.514841	1.526066
H	-0.914996	-3.269467	0.237455
H	0.494964	-2.238903	-0.169329
C	-0.462219	3.249950	-0.563510
H	-0.221911	3.640556	0.434420
H	0.176760	3.747816	-1.304442
H	-1.507424	3.482575	-0.783438
O	-0.315061	1.846256	-0.621664
H	0.610439	1.614892	-0.377097
S	2.485706	-0.270370	0.113049
O	2.123906	1.159905	0.329339
O	1.733163	-0.866652	-1.022614
C	4.208733	-0.266382	-0.361394
H	4.312106	0.320085	-1.275786
H	4.785561	0.181322	0.449510
H	4.520754	-1.298522	-0.529392
O	2.389246	-1.079328	1.350383

INT2B
36

C	-2.316019	1.137230	-0.123303
O	-2.536248	2.201813	0.358615
O	-2.853808	0.602271	-1.213158
N	-1.462581	0.123582	0.480568
C	-2.352246	-0.730081	-1.433183
C	-3.544531	-1.685936	-1.423593
H	-4.240188	-1.381757	-2.209629
H	-3.217649	-2.708801	-1.628274
H	-4.069781	-1.661446	-0.464019
C	-1.565875	-0.765706	-2.742109
H	-1.120552	-1.754281	-2.885834
H	-2.254513	-0.563660	-3.567081
H	-0.780221	-0.007950	-2.732468
C	-1.451647	-0.947038	-0.244097
C	-0.709441	0.461005	1.685336
H	-0.189332	-0.421029	2.054501
H	0.044572	1.204846	1.412661
H	-1.407665	0.869048	2.416900

C	-0.665257	-2.164377	-0.029986
H	-0.403701	-2.332462	1.014853
H	-1.165492	-3.032651	-0.463480
H	0.292648	-1.982271	-0.555423
C	0.342834	3.039066	-1.144964
H	-0.575772	3.625328	-1.062536
H	0.916662	3.121551	-0.215464
H	0.945030	3.432617	-1.974029
O	-0.037982	1.694219	-1.387358
H	0.737322	1.117810	-1.255281
S	2.414423	-0.311824	0.316756
O	1.739779	-0.588902	-0.996280
O	1.973678	-1.255268	1.369089
C	4.152821	-0.628492	0.044035
H	4.273129	-1.671078	-0.254923
H	4.506294	0.040590	-0.742184
H	4.682560	-0.434823	0.978357
O	2.293765	1.114516	0.688509

INT3
36

C	2.575334	1.536009	0.696517
O	2.885317	2.561445	1.250610
O	3.221027	0.355523	0.882008
N	1.547637	1.340356	-0.196945
C	2.748502	-0.610591	-0.087151
C	3.728321	-0.575392	-1.260429
H	4.731839	-0.789806	-0.882620
H	3.473013	-1.327231	-2.013210
H	3.747197	0.410012	-1.735908
C	2.727365	-1.989123	0.549498
H	2.316911	-2.716285	-0.158414
H	3.748320	-2.290207	0.801580
H	2.123585	-2.019742	1.456689
C	1.339974	-0.051028	-0.461431
C	0.591086	2.379408	-0.527347
H	-0.406701	2.136439	-0.147567
H	0.938007	3.305135	-0.065556
H	0.530954	2.514365	-1.610964
C	0.879135	-0.348352	-1.878204
H	1.581452	0.058640	-2.607553
H	0.798737	-1.429465	-2.027339
H	-0.101739	0.098014	-2.062346
C	0.198069	-0.142670	1.730819
H	-0.085321	0.911948	1.766717
H	-0.597404	-0.736053	2.183447
H	1.130650	-0.297537	2.280221
H	-1.180583	-1.049711	-0.190340
S	-2.862366	0.195150	-0.640734
O	-2.131693	-1.216527	-0.464726

O	-2.463140	0.799714	-1.903585
C	-4.533912	-0.392802	-0.768173
H	-4.602930	-1.062925	-1.625715
H	-4.796861	-0.904649	0.157859
H	-5.164301	0.485889	-0.916366
O	-2.698838	0.974091	0.579188
O	0.300689	-0.604266	0.381420

TS1

36

C	-2.392347	0.818178	0.559446
O	-2.801290	1.876017	0.937053
O	-2.849462	0.115491	-0.478046
N	-1.370329	0.052279	1.186838
C	-2.117611	-1.120026	-0.627877
C	-3.109708	-2.272978	-0.499586
H	-3.864169	-2.183854	-1.284930
H	-2.593611	-3.230214	-0.616317
H	-3.611496	-2.257426	0.473089
C	-1.390008	-1.099629	-1.968602
H	-0.782021	-2.001059	-2.081387
H	-2.132382	-1.066082	-2.771165
H	-0.745758	-0.219380	-2.029690
C	-1.154570	-1.075844	0.534834
C	-0.617292	0.559753	2.324877
H	-0.950596	1.581757	2.503836
H	-0.823456	-0.056913	3.205171
H	0.451080	0.543169	2.094584
C	-0.140134	-2.009986	0.793861
H	0.296132	-2.000656	1.792445
H	-0.301291	-3.001960	0.370802
H	0.808134	-1.569056	0.099225
C	-0.478401	3.115500	-1.153010
H	-0.045218	3.774397	-0.389033
H	-0.100000	3.415288	-2.139538
H	-1.564240	3.234268	-1.137348
O	-0.199074	1.755280	-0.883208
H	0.769849	1.628168	-0.850331
S	2.733111	-0.104940	0.010167
O	2.534038	1.192361	-0.660889
O	1.877234	-1.189667	-0.648900
C	4.398970	-0.622916	-0.349755
H	4.511586	-0.682611	-1.432896
H	5.078630	0.122746	0.066880
H	4.563863	-1.597010	0.113118
O	2.546804	-0.110170	1.468068

TS2

36

C	2.336999	1.503276	-0.030176
O	2.374829	2.693227	0.006117
O	3.265653	0.669384	0.421771
N	1.275877	0.725609	-0.568487
C	2.938561	-0.698589	0.068801
C	3.778725	-1.079191	-1.157512
H	4.831681	-0.963922	-0.899887
H	3.595919	-2.120067	-1.427781
H	3.553066	-0.441804	-2.015025
C	3.245916	-1.595717	1.249302
H	2.840817	-2.593834	1.074395
H	4.327862	-1.675982	1.361940
H	2.820807	-1.196861	2.167685
C	1.490591	-0.568451	-0.378605
C	0.122298	1.330162	-1.227983
H	-0.797536	0.876908	-0.859933
H	0.129451	2.393690	-1.000751
H	0.200512	1.182623	-2.305406
C	0.780239	-1.613502	-1.158945
H	1.185035	-1.591113	-2.177337
H	0.960266	-2.600281	-0.735503
H	-0.292415	-1.419007	-1.204531
C	0.269123	0.623884	1.599744
H	0.540720	1.551301	0.907268
H	-0.835092	0.947774	1.854686
H	0.808702	0.995051	2.577014
O	0.531646	-0.872531	1.107291
H	-0.519196	-0.971624	0.352981
S	-3.379477	-0.020162	0.037952
O	-2.315283	-1.027304	-0.271165
O	-3.293437	1.153537	-0.849316
C	-4.924937	-0.835401	-0.333779
H	-4.923511	-1.112484	-1.386918
H	-5.008807	-1.719013	0.297178
H	-5.734159	-0.137373	-0.123864
O	-3.408443	0.315670	1.474086ù

PRODUCT
36

O	-0.447802	0.260398	-1.017850
C	-0.344011	1.514322	-0.286033
C	-1.840600	1.935642	-0.132177
N	-2.462408	0.634398	-0.183250
C	-1.670280	-0.276793	-0.814621
C	0.320664	1.187302	1.047935
C	0.499803	2.488853	-1.083004
C	-2.158395	2.709284	1.134739
O	-2.295515	2.782772	-1.185101
C	-2.510844	2.216054	-2.470048
C	-3.867485	0.406898	0.086924

O	-1.959028	-1.397325	-1.163052
O	-5.717151	3.035208	-1.764560
S	-5.643856	4.031325	-0.722736
C	-4.362928	5.195436	-1.095942
O	-5.538618	3.622647	0.664592
O	-6.991455	4.907025	-0.885906
H	1.292265	0.730082	0.852062
H	0.478004	2.092217	1.637455
H	-0.279557	0.486410	1.632125
H	0.534500	3.448049	-0.561579
H	1.519227	2.106961	-1.165971
H	0.106263	2.657689	-2.083256
H	-4.096091	-0.627284	-0.165603
H	-4.083963	0.574131	1.143680
H	-4.493348	1.071315	-0.513943
H	-3.217805	2.968614	1.144991
H	-1.929384	2.124775	2.024856
H	-1.576710	3.633072	1.155495
H	-1.617401	1.726287	-2.867307
H	-3.339708	1.503950	-2.460610
H	-2.777215	3.048401	-3.120784
H	-7.223237	5.334933	-0.051277
H	-4.539528	5.584358	-2.096995
H	-4.387797	5.988436	-0.350568
H	-3.429270	4.630991	-1.047409

DFT ωB97-XD/6-311++G(d,p) Optimized Cartesian Coordinates fro the Hydrolysis of a N,O-acetal moiety

Reac
39

C	1.38640	1.81740	-0.16330
O	2.59970	1.38100	-0.84590
C	3.02220	0.23520	-0.29350
C	0.82700	0.47090	0.38450
C	0.49780	2.50780	-1.17670
H	-0.43930	2.80900	-0.70220
H	0.99650	3.40800	-1.54110
H	0.27460	1.85180	-2.01550
C	1.81700	2.77060	0.94520
H	2.37220	3.59870	0.50170
H	0.94780	3.17790	1.46450
H	2.45980	2.27320	1.67480
O	4.11300	-0.24620	-0.53170
N	2.07440	-0.25480	0.54240

C	2.31070	-1.43170	1.35950
H	2.32540	-2.34640	0.76330
H	3.26870	-1.33090	1.87130
H	1.52440	-1.50830	2.10850
C	0.01700	0.58910	1.66460
H	-0.47910	-0.35220	1.90210
H	-0.76410	1.33840	1.52600
O	0.02520	-0.08290	-0.65160
H	-4.03880	-2.26260	-0.99460
S	-3.81700	-0.40420	0.07380
O	-5.23240	-0.16790	0.27090
O	-3.62560	-1.40830	-1.18060
C	-3.03320	1.00980	-0.63980
H	-3.11360	1.80340	0.10270
H	-1.98890	0.75760	-0.83130
H	-3.56960	1.27820	-1.54750
O	-3.04320	-0.90140	1.19410
H	0.64230	0.88260	2.50800
C	-0.17040	-1.48760	-0.63560
H	-0.93920	-1.69460	-1.37870
H	0.74630	-2.01600	-0.91200
H	-0.52570	-1.84360	0.33650
O	4.76400	-2.98640	-0.38130
H	5.43060	-3.12960	-1.05460
H	4.56860	-2.03600	-0.42110

Int1
39

C	2.82560	0.65850	0.34210
O	3.53080	-0.25750	-0.53420
C	2.86640	-1.39000	-0.66920
C	1.57970	-0.11770	0.66900
C	2.56640	1.95480	-0.41110
H	1.93710	2.61340	0.18990
H	3.52150	2.44970	-0.59110
H	2.06800	1.75190	-1.35670
C	3.66740	0.86350	1.60080
H	4.63380	1.27340	1.30640
H	3.17350	1.57550	2.26350
H	3.82530	-0.07530	2.13490
O	3.17390	-2.33910	-1.31110
N	1.66680	-1.28980	0.12980
C	0.68780	-2.37430	0.17980
H	-0.21310	-2.06480	-0.35220
H	1.13970	-3.24050	-0.29660
H	0.44940	-2.59350	1.21830
C	0.51810	0.34330	1.56670
H	-0.34120	-0.32820	1.58250
H	0.19930	1.34470	1.26190

O	-1.12090	2.57740	-0.09990
H	-1.88580	1.99230	0.07620
S	-2.91060	-0.69300	0.15120
O	-2.32880	-1.28970	1.36860
O	-2.15180	-1.05350	-1.07540
C	-4.53760	-1.39480	-0.04960
H	-5.12620	-1.14380	0.83150
H	-4.98530	-0.96970	-0.94650
H	-4.43120	-2.47420	-0.14650
O	-3.11290	0.77450	0.26920
H	0.94350	0.42340	2.57380
C	-1.58560	3.81450	-0.61270
H	-2.15840	3.67620	-1.53600
H	-2.21060	4.33850	0.11770
H	-0.71340	4.43170	-0.83250
O	0.07400	0.54640	-1.58750
H	-0.69290	-0.05140	-1.54820
H	-0.27470	1.37270	-1.21040

TS1
39

C	2.30490	-0.61820	-0.76140
O	1.53470	-1.76340	-0.32720
C	0.98040	-1.53340	0.85820
C	1.88450	0.45740	0.24270
C	1.99000	-0.35900	-2.22010
H	2.46870	0.56420	-2.55140
H	2.38950	-1.18090	-2.81550
H	0.91490	-0.30150	-2.38160
C	3.78420	-0.94750	-0.54780
H	4.02190	-1.84140	-1.12480
H	4.41260	-0.12840	-0.90100
H	4.00400	-1.14040	0.50420
O	0.37140	-2.32300	1.51270
N	1.27570	-0.20470	1.23380
C	0.83740	0.34740	2.50900
H	0.00130	1.03260	2.35630
H	0.51940	-0.48590	3.13170
H	1.66910	0.86070	2.99070
C	2.73010	1.65340	0.50780
H	2.18430	2.40060	1.08120
H	3.08180	2.09210	-0.42500
O	0.40150	1.38850	-0.49410
H	-0.32250	0.68790	-0.66520
S	-2.69530	-0.38730	-0.49350
O	-3.07050	0.98600	-0.08870
O	-1.23740	-0.45090	-0.86640
C	-2.83880	-1.40960	0.95980
H	-3.88220	-1.40280	1.27160

H	-2.51690	-2.41710	0.70270
H	-2.19960	-0.99420	1.73720
O	-3.55040	-0.96420	-1.52950
H	3.60000	1.33400	1.08730
C	0.39020	2.40300	-1.50270
H	-0.58940	2.88480	-1.48990
H	1.15240	3.14060	-1.25500
H	0.57960	1.98750	-2.49280
O	-1.54470	2.60930	1.56510
H	-0.76240	2.66530	1.01200
H	-2.14700	2.05260	1.03890

TS2
39

C	1.47370	0.05260	0.49200
N	1.63710	-1.24940	0.32130
C	2.75830	-1.50880	-0.50840
O	3.38080	-0.35660	-0.73200
C	2.76070	0.71090	0.02960
C	0.74010	-2.30550	0.78010
O	3.08530	-2.57790	-0.92040
C	2.63880	1.93940	-0.84830
C	3.63210	0.97650	1.26180
C	0.59950	0.61590	1.54650
O	0.26990	0.47650	-1.12770
O	-1.85740	-1.15300	-0.89260
S	-2.91220	-0.61290	0.01470
O	-2.48660	-0.62620	1.42550
O	-3.39340	0.71570	-0.43920
C	-4.29720	-1.72260	-0.13590
O	-1.42090	2.54920	-0.79610
C	-1.44100	3.47240	0.28060
H	2.05200	2.70470	-0.33630
H	3.63550	2.34210	-1.03310
H	2.16080	1.69450	-1.79280
H	4.62730	1.26510	0.92310
H	3.21170	1.79410	1.84940
H	3.71700	0.09000	1.89350
H	-0.23490	-2.18530	0.30590
H	1.18400	-3.25660	0.49570
H	0.64380	-2.25180	1.86390
H	-0.38930	0.15170	1.54520
H	0.50400	1.69350	1.42620
H	-2.20990	1.97420	-0.72210
H	-5.09430	-1.35510	0.50850
H	-4.61640	-1.73280	-1.17670
H	-3.97310	-2.71310	0.17930
H	1.07830	0.41510	2.51130
H	-2.24490	4.20450	0.15620

H	-1.57350	2.96450	1.24230
H	-0.48720	4.00260	0.28720
H	-0.45770	-0.18970	-1.10700
H	-0.21080	1.33250	-1.03210

Prod
39

C	-7.40488	-2.72964	2.86144
O	-8.51061	-2.10149	3.56793
C	-8.38242	-0.76320	3.49818
C	-6.98059	-1.60611	1.86883
C	-7.91454	-4.00143	2.21237
H	-7.12925	-4.44530	1.59630
H	-8.18459	-4.71783	2.99060
H	-8.78319	-3.80608	1.58735
C	-6.32175	-3.02812	3.89193
H	-6.74690	-3.66524	4.66900
H	-5.48478	-3.55700	3.43284
H	-5.94825	-2.11410	4.35872
O	-9.06190	0.00469	4.13577
N	-7.38169	-0.44969	2.62382
C	-7.02668	0.92861	2.35116
H	-7.75757	1.41098	1.69715
H	-6.97866	1.47482	3.29314
H	-6.04580	0.95878	1.87882
C	-5.52377	-1.62950	1.45369
H	-5.31584	-0.87812	0.69469
H	-5.28820	-2.60844	1.03284
O	-7.83565	-1.79935	0.71773
H	-6.66374	0.22802	-0.66208
N	-7.99056	-0.65740	-0.07293
C	-9.18846	-0.56259	-0.48974
H	-9.91312	-1.30393	-0.15345
C	-9.62523	0.51678	-1.40975
H	-10.40122	1.11119	-0.91877
H	-8.80364	1.16187	-1.71741
H	-10.07956	0.07093	-2.29875
S	-5.75655	1.97570	-1.70721
O	-4.42152	2.11622	-2.24363
O	-5.75907	0.61208	-0.89990
C	-6.00971	3.19869	-0.44310
H	-5.97595	4.17226	-0.93052
H	-6.98582	3.03341	0.00991
H	-5.20945	3.10648	0.28867
O	-6.89049	1.99993	-2.61432
H	-4.87422	-1.44347	2.30897

References

1. Kolibaba, T. J. *et al.* Results of an interlaboratory study on the working curve in vat photopolymerization. *Addit Manuf* **84**, 104082 (2024).

2. Jiang, H., Zhao, J. & Wang, A. An efficient and eco-friendly process for the conversion of carbon dioxide into oxazolones and oxazolidinones under supercritical conditions. *Synthesis (Stuttg)* **5**, 763–769 (2008).

3. Chao, A. & Zhang, D. Investigation of Secondary Amine-Derived Aminal Bond Exchange toward the Development of Covalent Adaptable Networks. *Macromolecules* **52**, 495–503 (2019).

4. Chai, J. Da & Head-Gordon, M. Long-range corrected hybrid density functionals with damped atom-atom dispersion corrections. *Physical Chemistry Chemical Physics* **10**, 6615–6620 (2008).

5. Krishnan, R., Binkley, J. S., Seeger, R. & Pople, J. A. Self-consistent molecular orbital methods. XX. A basis set for correlated wave functions. *J Chem Phys* **72**, 650–654 (1980).

6. Hehre, W. J., Ditchfield, K. & Pople, J. A. Self-consistent molecular orbital methods. XII. Further extensions of gaussian-type basis sets for use in molecular orbital studies of organic molecules. *J Chem Phys* **56**, 2257–2261 (1972).

7. Frisch, M. J. *et al.* Gaussian 16, revision A.03. Preprint at (2016).

8. Habets, T. *et al.* Covalent Adaptable Networks through Dynamic N,S-Acetal Chemistry: Toward Recyclable CO2-Based Thermosets. *J Am Chem Soc* **145**, 25450–25462 (2023).

9. Caliari, M. *et al.* Fully Recyclable Pluripotent Networks for 3D Printing Enabled by Dissociative Dynamic Bonds. *Advanced Materials* 2417355 (2025) doi:10.1002/ADMA.202417355.
